# Transcriptomic changes in the frontal cortex associated with paternal age

**DOI:** 10.1186/2040-2392-5-24

**Published:** 2014-03-23

**Authors:** Rebecca G Smith, Cathy Fernandes, Rachel Kember, Leonard C Schalkwyk, Joseph Buxbaum, Abraham Reichenberg, Jonathan Mill

**Affiliations:** 1 Institute of Psychiatry, King’s College London, De Crespigny Park, Denmark Hill, London SE5 8AF, UK; 2 Mount Sinai School of Medicine, Madison Avenue, New York, NY 10029, USA; 3 University of Exeter Medical School, St Luke’s Campus, Magdalen Rd, Exeter EX1 2LU, UK

**Keywords:** Autism, Advanced paternal age, Gene expression, Transcriptome, Inflammation, Immune response, Brain

## Abstract

**Background:**

Advanced paternal age is robustly associated with several human neuropsychiatric disorders, particularly autism. The precise mechanism(s) mediating the paternal age effect are not known, but they are thought to involve the accumulation of *de novo* (epi)genomic alterations. In this study we investigate differences in the frontal cortex transcriptome in a mouse model of advanced paternal age.

**Findings:**

Transcriptomic profiling was undertaken for medial prefrontal cortex tissue dissected from the male offspring of young fathers (2 month old, 4 sires, *n* = 16 offspring) and old fathers (10 month old, 6 sires, *n* = 16 offspring) in a mouse model of advancing paternal age. We found a number of differentially expressed genes in the offspring of older fathers, many previously implicated in the aetiology of autism. Pathway analysis highlighted significant enrichment for changes in functional networks involved in inflammation and inflammatory disease, which are also implicated in autism.

**Conclusions:**

We observed widespread alterations to the transcriptome associated with advanced paternal age with an enrichment of genes associated with inflammation, an interesting observation given previous evidence linking the immune system to several neuropsychiatric disorders including autism.

## Findings

### Background

Advanced paternal age is robustly associated with several neuropsychiatric disorders, most notably autism [1]. The mechanism(s) underlying this paternal age effect are not known, although the accumulation of *de novo* (epi)genetic changes over time in the male germ line are thought to be important. Evidence to support this hypothesis comes from preliminary data showing a higher rate of *de novo* mutations [2] and altered DNA methylation at birth [3] with increasing paternal age in humans, and increased *de novo* copy number variation (CNV) [4] and altered brain DNA methylation at imprinted loci in rodent models of advanced paternal age [5].

Gene expression changes associated with aging in the brain have been investigated largely in terms of neurodegeneration and dementia [6], although aging *per se* is associated with widespread transcriptional changes [7]. In a study of aging using mice, for example, brain samples from the cerebellum and neocortex had increased expression of genes related to inflammatory and stress responses and decreased expression of genes associated with growth and trophic factors, protein turnover, DNA synthesis and repair, and neurotransmission [8]. Age-associated changes have been observed in numerous tissue and cell types, including the germ line. Of relevance to paternal age, an expression study of rats of different ages identified over 2,800 loci that are differentially expressed in spermatocytes from older males (18 months) compared to spermatocytes from young males (4 months); of note, many genes associated with base excision repair, nucleotide excision repair, mismatch repair and double strand break repair were altered in spermatocytes from older males [9]. In an analysis of RNA from the spermatogonial stem cells of mice of four different ages (6 days, 21 days, 60 days and 8 months) using microarrays, 2,819 genes had differential expression between the age groups (*P* < 0.05, fold change >2) including genes previously identified in gene expression studies of aging in stem cells [10]. Pathway analysis of these genes highlighted an enrichment of genes involved in DNA repair and oxidative stress, which is interesting given the known increase in DNA damage in the spermatozoa of older males. To date, only one study has looked at gene expression changes associated with paternal age in humans [11]. In this study of paternal age and autism, a decrease in the overall variance of gene expression was observed in the offspring of older fathers, in addition to a downregulation of genes involved in gene transcription [11]. To date, no research has examined transcriptomic changes in the brain in the context of advanced paternal age.

### Methods

C57BL/6J mice were bred and maintained in the Biological Services Unit at the Institute of Psychiatry, Kings College London, using stocks purchased from Charles River Laboratories. All animal experiments received the approval of the local ethical review panel of King’s College London and were performed in compliance with the UK’s Animals (Scientific Procedures) Act 1986. The work was carried out under licence (PPL 70/7184) and all efforts were made to minimize animal suffering and to reduce the number of animals used. We used tissue collected from an experimentally controlled rodent model of advancing paternal age (described in detail previously [5]) to examine whether the offspring of older fathers have altered levels of gene expression. Male offspring of ‘young’ fathers (2 month old, *n* = 16, four sires with one litter each) and ‘old’ fathers (10 month old, *n* = 16, six sires with one litter each), with at least two individuals selected from each family, were used in this study (see Additional file 1 for an overview of the samples used). All dissections were performed by a single individual to ensure topographical similarity between samples. Briefly, brains were removed from the skull and placed dorsal side down on a wetted filter paper on a petri dish kept on ice. The cerebral halves were opened out from the midline, after cutting through the corpus callosum. Approximately 3 mm^3^ of tissue was cut from the anterior part of the frontal lobes (from bregma 2.46 mm to 1.34 mm [12]), mainly containing the medial prefrontal cortex including some prelimbic cortex, infralimbic cortex, cingulate cortex and motor cortex [13].

High quality RNA (average RNA integrity number (RIN) 8.4) was isolated using the Qiagen AllPrep DNA/RNA Micro Kit (Qiagen, Manchester, UK). Gene expression was quantified using the Illumina Mouse Ref8 V2 array (Illumina, San Diego, CA, USA), which targets approximately 25,600 transcripts and over 19,100 unique genes. After stringent quality control and preprocessing, raw expression data were batch-corrected using the ComBat package [14] before being normalized and analysed using *lumi*[15] within the R statistical environment. All analysis scripts are available on request from the authors.

### Results and discussion

**Figure 1:**
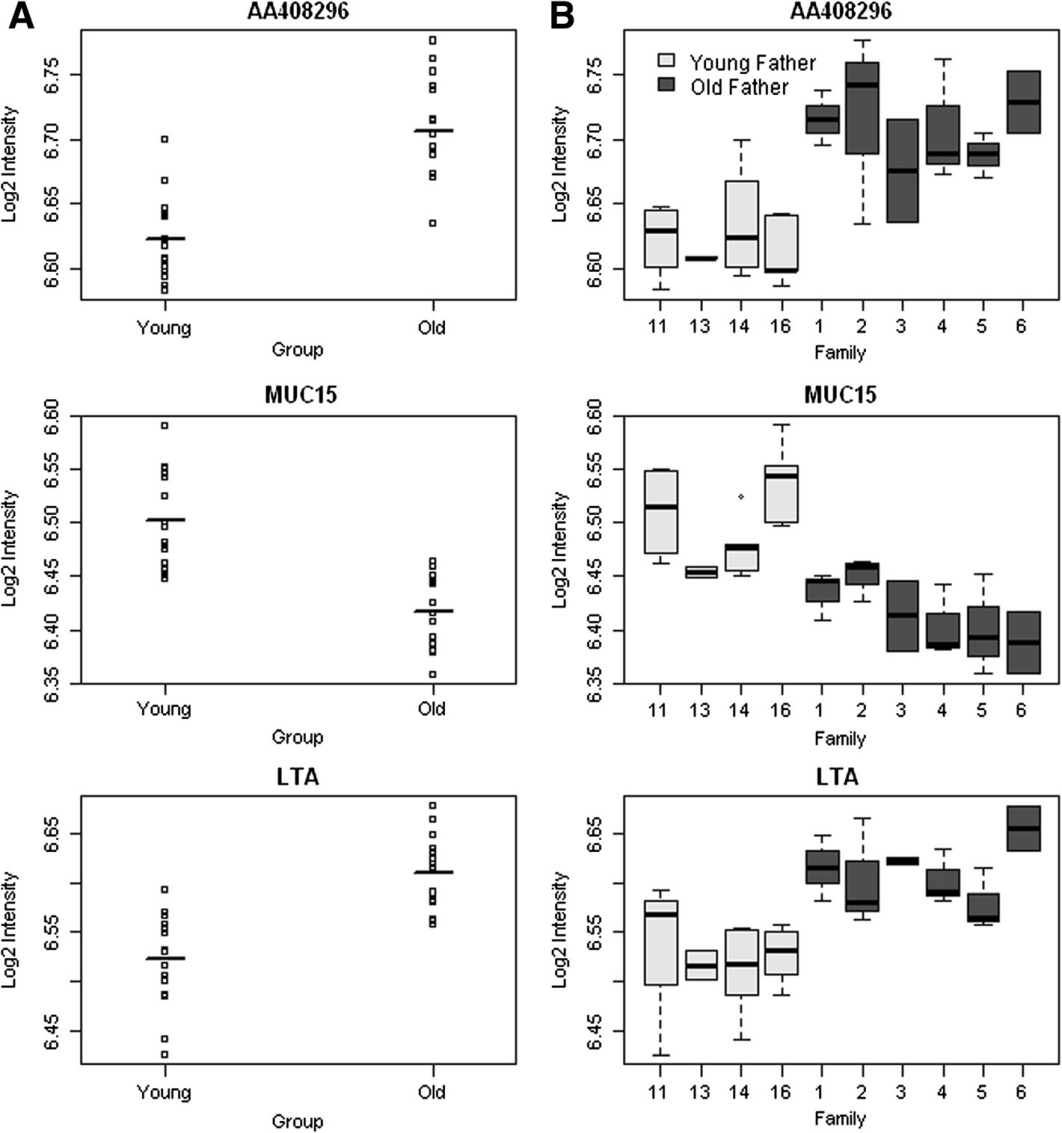
**Top-ranked differentially expressed transcripts between the offspring of young fathers and offspring of old fathers.** Shown for each probe (associated with the genes *AA408296*, *Muc15* and *Lta*) are **(A)** group-level differences and **(B)** data split by family. The *y*-axis shows the log_2_ transformed signal intensity.

We found numerous differences in gene expression associated with advanced paternal age. Most notably, probes associated with three transcripts were found to be differentially expressed in the offspring of older fathers compared to the offspring of younger fathers with a false discovery rate (FDR) < 0.1: *AA408296* (also known as *Diexf*) (*P* = 3.23 × 10^-6^, FDR = 0.06) and *Lta* (*P* = 3.23 × 10^-6^, FDR = 0.06) had elevated expression, whilst *Muc15* (*P* = 7.46 × 10^-6^, FDR = 0.06) had reduced expression (see Figure 1A). Although the absolute differences in gene expression are small and future work is needed to ascertain their functional significance, for each of these differentially expressed transcripts, consistent patterns of altered expression were observed across each of the old-father families (Figure 1B and Additional file 2). The top-ranked differentially expressed transcripts (*P* < 0.001) are listed in Table 1. Strikingly, almost a quarter of these differentially expressed loci have been previously implicated in the aetiology of autism, including *Gstm1* (deletion in *GSTM1* observed in autism case-parent trios) [16], *Ccdc90b* (missense mutation associated with autism in a study of cases and their parents) [17], *Cd44* (differential expression in autism discordant monozygotic twins) [18], *Accn1* (multiple SNPs associated with autism in a familial study) [19], *Shh* (increased serum *SHH* expression in autistic patients compared to non-autistic controls) [20], *Dus1l* (*de novo* missense mutation observed in autism families) [21] and *Ier5l* (increased expression in lymphoblast cells from autism patients in discordant sibling pairs) [22].

**Table 1:** **Probes showing gene expression differences associated with paternal age (*P*** **< 0.001)**

**Transcript**	**Probe ID**	**Expression difference**^**a**^	***P *** **value**	**Mean (SEM) young**	**Mean (SEM) old**
*AA408296*	4150356	**↑**	3.23 × 10^-6^	6.62 (0.01)	6.71 (0.01)
*Muc15*	2120176	**↓**	7.45 × 10^-6^	6.5 (0.01)	6.42 (0.01)
*Lta*	2900474	**↑**	7.46 × 10^-6^	6.52 (0.01)	6.61 (0.01)
*Tslp*	6960520	**↑**	3.02 × 10^-5^	6.41 (0.01)	6.48 (0.01)
*Zfp35*	580168	**↓**	1.30 × 10^-4^	6.99 (0.02)	6.89 (0.01)
*Gstm1*	4540458	**↑**	1.31 × 10^-4^	6.57 (0.02)	6.66 (0.01)
*Rdh20*	6840020	**↑**	1.38 × 10^-4^	6.48 (0.01)	6.56 (0.01)
*Ccdc90b*	6100215	**↑**	1.91 × 10^-4^	7.1 (0.02)	7.22 (0.02)
*Sdhc*	3290630	**↓**	2.10 × 10^-4^	6.48 (0.01)	6.4 (0.02)
*Atp5sl*	5270131	**↓**	2.97 × 10^-4^	7.76 (0.02)	7.61 (0.03)
*Olfr187*	380370	**↓**	3.07 × 10^-4^	6.59 (0.01)	6.51 (0.01)
*Cd44*	4880138	**↓**	3.29 × 10^-4^	6.64 (0.01)	6.57 (0.01)
*Fryl*	510059	**↑**	3.58 × 10^-4^	6.57 (0.01)	6.64 (0.01)
*Accn1*	7330561	**↑**	3.59 × 10^-4^	6.69 (0.01)	6.76 (0.01)
*Cd177*	670452	**↓**	3.71 × 10^-4^	6.51 (0.01)	6.43 (0.01)
*Tspan3*	3130497	**↑**	3.96 × 10^-4^	10.33 (0.04)	10.49 (0.03)
*Olfr92*	2710201	**↑**	4.29 × 10^-4^	6.47 (0.01)	6.53 (0.01)
*Shh*	2260450	**↑**	4.37 × 10^-4^	6.71 (0.02)	6.79 (0.01)
*E130303B06RIK*	6250669	**↓**	4.38 × 10^-4^	6.67 (0.01)	6.59 (0.01)
*Arhgap6*	7550333	**↓**	4.43 × 10^-4^	6.45 (0.01)	6.39 (0.01)
*Zc3h18*	7380382	**↑**	5.21 × 10^-4^	7 (0.01)	7.08 (0.02)
*Atg5*	3850450	**↓**	6.10 × 10^-4^	7.98 (0.02)	7.9 (0.01)
*Nsfl1c*	7610433	**↓**	7.45 × 10^-4^	7.69 (0.02)	7.55 (0.03)
*Ear12*	4540543	**↓**	7.47 × 10^-4^	6.52 (0.01)	6.46 (0.01)
*Olfr1366*	6860093	**↓**	7.69 × 10^-4^	6.49 (0.02)	6.41 (0.01)
*Tnfsf11*	2480255	**↑**	8.20 × 10^-4^	6.5 (0.01)	6.57 (0.01)
*Lcorl*	2470343	**↓**	8.27 × 10^-4^	6.56 (0.01)	6.49 (0.01)
*Dus1l*	2370138	**↑**	9.30 × 10^-4^	7.13 (0.01)	7.21 (0.02)
*Larp4*	6400025	**↓**	9.59 × 10^-4^	6.47 (0.01)	6.41 (0.01)
*Alg12*	3850500	**↑**	9.79 × 10^-4^	6.56 (0.01)	6.65 (0.02)
*Ier5l*	4900053	**↓**	9.82 × 10^-4^	7.6 (0.02)	7.47 (0.03)
*Ccl26*	4560164	**↓**	9.94 × 10^-4^	6.49 (0.01)	6.41 (0.02)

^a^Arrows denote direction of change in the offspring of older father compared to the offspring of younger fathers.

SEM, standard error of the mean.

**Figure 2:**
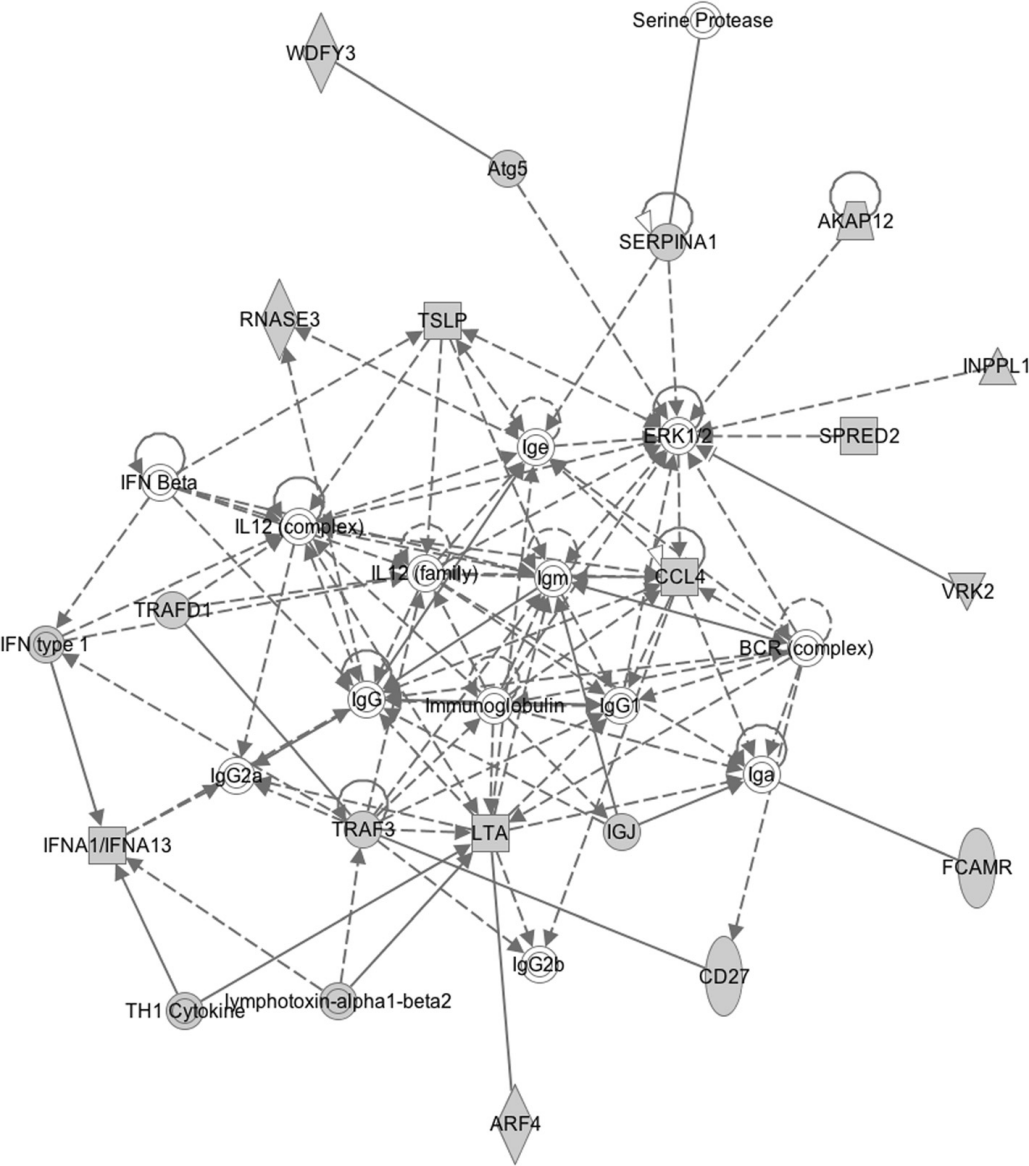
**Top-ranked gene network.** The network was derived from Ingenuity Pathway Analysis of differentially expressed genes in the offspring of older fathers in a mouse model of advanced paternal age. This network is involved in immune cell trafficking and cell-to-cell signalling. Different shapes relate to different molecule types. The key from Ingenuity can be found at [30].

Ingenuity pathway analysis (IPA) [23] and the Database for Annotation, Visualization and Integrated Discovery (DAVID) [24,25] were used to identify gene pathways and functions enriched amongst transcripts differentially expressed in the offspring of old fathers. Of note, IPA identified a significant enrichment for functional pathways involved in inflammation and inflammatory disease. Table 2 shows the top-ranked biological functions enriched in probes differentially expressed (*P* < 0.01) in the frontal cortex in the offspring of old fathers. The top-ranked biological network regulates immune cell trafficking and cell-to-cell signalling (Figure 2). Furthermore, the most significantly associated gene ontology term identified by DAVID was immune response (14 genes, *P* = 2.87 × 10^-4^) and the second most associated cluster (after cytokine activity) included defence response, response to wounding, inflammatory response and acute inflammatory response. This is interesting given mounting evidence linking the immune system to several neuropsychiatric disorders including autism [26-28]. A recent transcriptomic analysis of post-mortem brains from autistic patients, for example, showed changes in gene networks involved in immune and inflammatory responses in the frontal and temporal cortices [29].

**Table 2:** Top-ranked biological functions enriched in probes differentially expressed (P < 0.01) from IPA

**Biological function**	***P *** **value**
Maturation of dendritic cells	8.99 × 10^-5^
Influx of phagocytes	1.69 × 10^-4^
Binding of interferon-stimulated response element	6.43 × 10^-4^
Damage of oligodendrocytes	7.00 × 10^-4^
Lack of mesenteric lymph node	7.00 × 10^-4^
Lack of peripheral lymph node	7.00 × 10^-4^
Stimulation of hyaluronic acid	7.00 × 10^-4^
Morphology of tooth	7.56 × 10^-4^
Activation of myeloid cells	7.78 × 10^-4^
Clustering of lymph node cells	1.16 × 10^-3^
Osteoclastogenesis of bone cell lines	1.16 × 10^-3^
Transmigration of Th1 cells	1.16 × 10^-3^
Influx of neutrophils	1.21 × 10^-3^
Proliferation of dendritic cells	1.27 × 10^-3^
Destruction of joint	1.47 × 10^-3^
Arrest in cell cycle progression of endothelial cells	1.73 × 10^-3^
Lack of cervical lymph node	1.73 × 10^-3^
Organogenesis of lymphoid organ	1.73 × 10^-3^
Response of carcinoma cell lines	1.73 × 10^-3^
Extravasation of myeloid cells	1.92 × 10^-3^
Morphogenesis of neurites	1.97 × 10^-3^
Induction of neuroglia	2.40 × 10^-3^
Quantity of IL-5 In blood	2.40 × 10^-3^
Quantity of multinucleated cells	2.40 × 10^-3^
Response of lung cancer cell lines	2.40 × 10^-3^
Activation of monocytes	2.45 × 10^-3^
Acne	2.48 × 10^-3^
Formation of osteoclasts	2.60 × 10^-3^
Mobilization of blood cells	2.63 × 10^-3^
Activation of phagocytes	2.69 × 10^-3^
Binding of lymphoma cell lines	2.76 × 10^-3^
Cell viability of blood cells	2.82 × 10^-3^
Quantity of tooth	3.17 × 10^-3^
Activation of macrophages	3.23 × 10^-3^
Proliferation of B lymphocytes	3.35 × 10^-3^
Response of connective tissue cells	3.42 × 10^-3^
Cell movement of dendritic cells	3.88 × 10^-3^

In addition to identifying group-level variation between the offspring of old and young fathers, we also examined family-specific gene expression differences, as many of the genetic (or epigenetic) alterations believed to underlie the paternal age effect in autism are thought likely to be sporadic *de novo* events occurring in single litters. We compared the average transcript level for all offspring within each family to the average across all offspring of young fathers. We found a 1.8-fold enrichment in the number of family-specific significant gene expression differences in the offspring of older fathers (average 426 differentially expressed genes) compared to the offspring of young fathers (average 234 differentially expressed genes) (*P* < 0.001). Significant probes for each of the six old-father families are listed in Additional file 3. Of note, a number of probes were differentially expressed in the offspring of more than one advanced-age father including probes representing *Myst1*, *Tnfsf11* and *Fryl* (see Additional file 4).

### Conclusions

We present evidence for transcriptomic differences in the medial prefrontal cortex of offspring of old fathers compared to the offspring of young fathers, including for genes previously implicated in autism, a neuropsychiatric disease epidemiologically associated with advanced paternal age, and an enrichment of loci involved in the inflammatory response. Previous studies of gene expression changes associated with age in the mouse brain have shown enrichment for genes associated with the inflammatory response [8], although this is the first study to examine differences associated with advanced paternal age. Future work will examine whether these expression differences result from *de novo* genetic (or epigenetic) alterations occurring in the sperm of older fathers.

## Availability of supporting data

The data set supporting the results is available in the Gene Expression Omnibus repository (Currently awaiting upload) or downloaded from our webpage epigenomicslab.com/Paternal Age data.rar.

## Abbreviations

CNV: copy number variation; DAVID: Database for Annotation, Visualization and Integrated Discovery; FDR: false discovery rate; IL: interleukin; IPA: Ingenuity Pathway Analysis; RIN: RNA integrity number; SNP: single nucleotide polymorphism.

## Competing interests

The authors declare that they have no competing interests.

## Authors’ contributions

All authors contributed to the design of the study. RS undertook the lab work. RS and JM drafted the article. RS collected and analysed data, wrote the manuscript, conceived and designed the study, made a critical revision and gave final approval of the manuscript. CF collected data, conceived and designed the study, made a critical revision and gave final approval of the manuscript. RK collected data, made a critical revision and gave final approval of the manuscript. LS analysed the data, made a critical revision and gave final approval of the manuscript. JB and AR conceived and designed the study, provided financial support, made a critical revision and gave final approval of the manuscript. JM wrote the manuscript, conceived and designed the study, made a critical revision and gave final approval of the manuscript. All authors read and approved the final manuscript.

## Supplementary Material

Additional file 1 Overview of the samples used in this study. Click here for file

Additional file 2 **Significant gene expression differences for individual offspring split by sire for ‘young’ and ‘old’ fathers were seen for (A) *AA408296, *** **(B) *Muc15 *** **and (C) *Lta.***
Click here for file

Additional file 3 **Top-ranked differentially expressed transcripts (*P* < 0.001) within each of the six families with an old father.** Shown for each transcript is the corresponding rank in the overall old vs young father group comparison. Click here for file

Additional file 4 **Comparison of the top-ranked differentially expressed transcripts (*P* < 0.001) across each of the six families with an old father.** Shown for each transcript is the corresponding rank in the overall old vs young father group comparison. Empty cells indicate a non-significant difference. Click here for file
