# Supplementary material for: Transcriptomic changes in the frontal cortex associated with paternal age
Source: Mol Autism. 2014 Mar 23;5:24. doi: 10.1186/2040-2392-5-24 (PMC3998024; doi:10.1186/2040-2392-5-24)
Supplement: Additional file 1 — Overview of the samples used in this study. [file 2040-2392-5-24-S1.pdf]

Additional File 1 – Overview of the samples used in this study.

Sample	Sire / Family	Father Group	RIN
1	Y1	Young	8.4
2	O1	Old	8.4
3	Y1	Young	8.5
4	O2	Old	8.4
5	Y1	Young	8.5
6	O2	Old	8.6
7	Y2	Young	8.2
8	O3	Old	8.4
9	Y3	Young	8.2
10	O3	Old	8.5
11	Y3	Young	8.6
12	O4	Old	8.9
13	Y3	Young	8.2
14	Y3	Young	8.6
15	Y4	Young	8.5
16	O5	Old	8.5
17	Y4	Young	8.6
18	O5	Old	8.6
19	Y4	Young	8.6
20	O6	Old	6.1
21	Y4	Young	8.7
22	Y1	Young	8.3
23	O1	Old	8.5
24	Y2	Young	8.4
25	O1	Old	8.6
26	Y3	Young	8.9
27	O2	Old	N/A
28	Y4	Young	8.9
29	O4	Old	8.5
30	O4	Old	N/A
31	O5	Old	8
32	O6	Old	N/A
