# Supplementary figures and images for: Transcriptomic changes in the frontal cortex associated with paternal age
Source: Mol Autism. 2014 Mar 23;5:24. doi: 10.1186/2040-2392-5-24 (PMC3998024; doi:10.1186/2040-2392-5-24)

A

AA408296

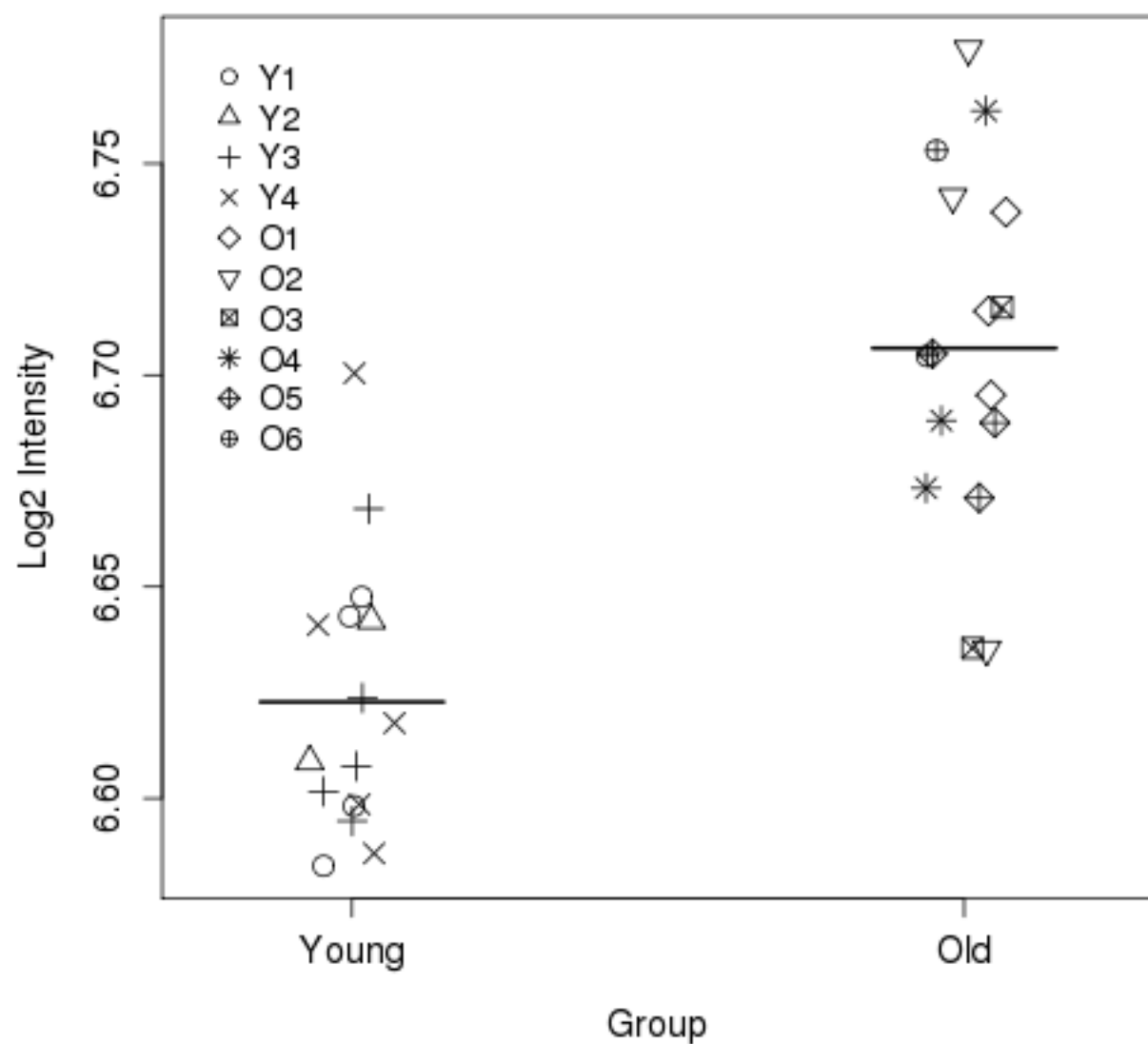

B

# MUC15

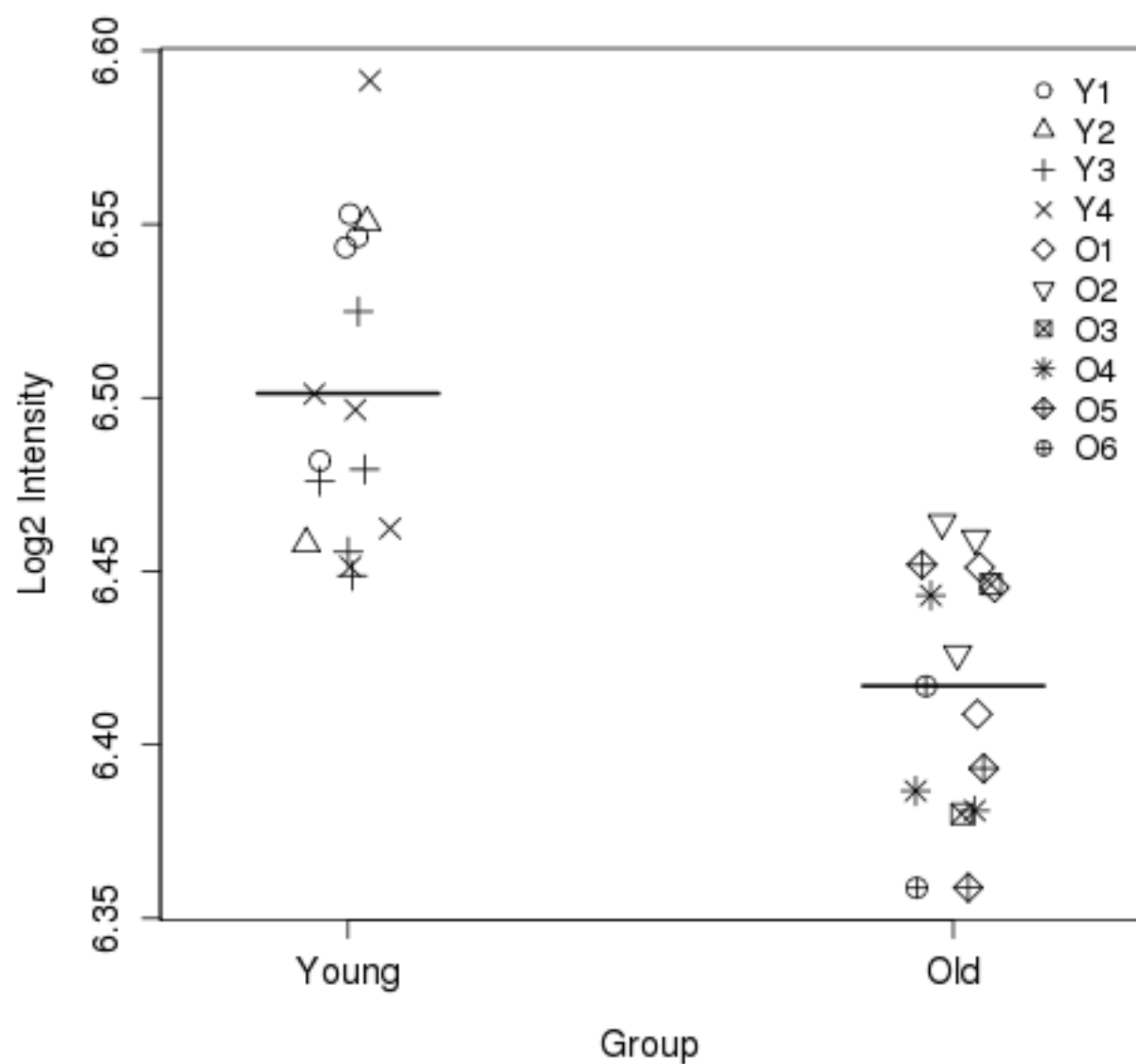

C

## LTA

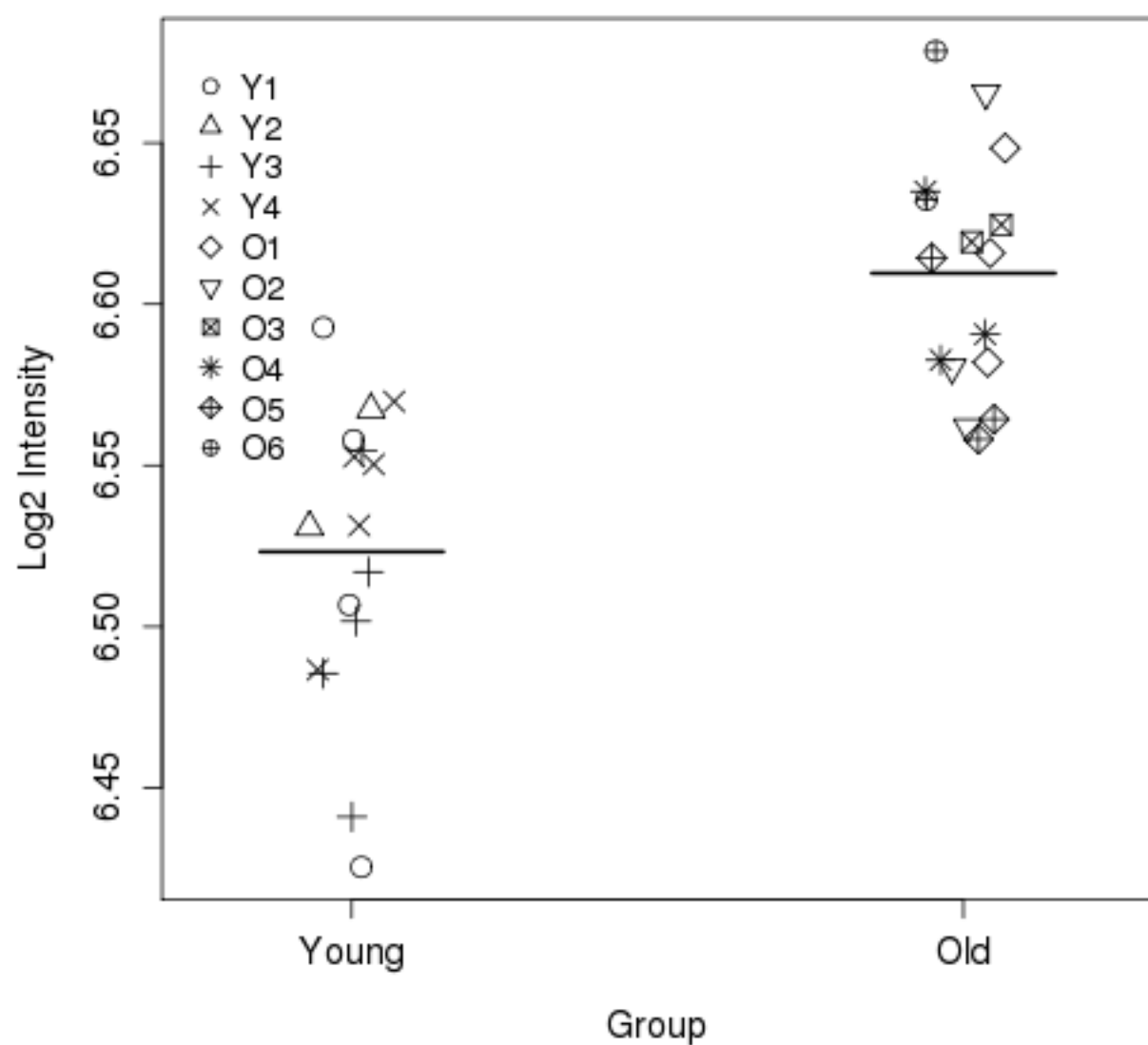

Supplement: Additional file 2 — Significant gene expression differences for individual offspring split by sire for ‘young’ and ‘old’ fathers were seen for (A) AA408296, (B) Muc15 and (C) Lta. [file 2040-2392-5-24-S2.pdf]
