# Supplementary material for: Transcriptomic changes in the frontal cortex associated with paternal age
Source: Mol Autism. 2014 Mar 23;5:24. doi: 10.1186/2040-2392-5-24 (PMC3998024; doi:10.1186/2040-2392-5-24)
Supplement: Additional file 3 — Top-ranked differentially expressed transcripts ( P < 0.001) within each of the six families with an old father. Shown for each transcript is the corresponding rank in the overall old vs young father group comparison. [file 2040-2392-5-24-S3.pdf]

Family 1

Group Rank	Gene	Probe	Fold Chang	P-Value
2891	OLFR1449	5690400	0.93	1.06E-08
17821	RARG	620240	1.09	1.21E-07
12638	IGFBPL1	580767	0.92	1.30E-07
18783	TRAPPC5	4760128	0.93	5.81E-07
323	EG331493	6480477	0.93	6.60E-07
2267	PARVG	3310612	1.10	7.67E-07
362	EYA1	3800678	1.07	2.60E-06
4128	SEMA7A	2570288	1.09	3.40E-06
9073	PRKCBP1	7400408	1.22	4.94E-06
2567	SULT1C2	1580377	0.95	6.30E-06
16087	PIP4K2B	2120086	1.25	6.30E-06
1272	UCP1	2630639	0.92	7.45E-06
8274	ZFP251	60059	1.15	7.71E-06
10841	RILPL1	450092	1.14	7.71E-06
23824	SIPA1	4760725	0.94	7.88E-06
10485	B230342M	7160370	0.90	7.97E-06
3567	DEFB4	7000368	0.92	8.33E-06
1836	SORBS1	520484	1.06	1.04E-05
4219	3110040N1	4250202	0.94	1.09E-05
691	1190002A1	4670386	0.94	1.28E-05
2955	MTF1	6510189	1.06	1.28E-05
3365	KLRA19	6580280	0.95	1.56E-05
3264	OLFR559	1050397	1.05	1.57E-05
10352	ECE1	5700722	1.13	1.62E-05
12390	OGDH	940168	1.21	1.64E-05
2088	OLFR1250	20372	0.95	1.90E-05
467	NSUN5	4920730	1.05	2.02E-05
17801	2310033P0	4900070	1.14	2.23E-05
2149	B3GALT4	3870202	0.92	2.25E-05
1025	BC057627	4220739	1.08	2.35E-05
1672	CD109	3460575	0.94	2.45E-05
10264	EBPL	110630	0.86	2.64E-05
11887	PSME3	2940097	1.18	3.64E-05
10657	5830457O1	1990008	1.08	3.70E-05
5853	NME7	4490678	0.92	3.92E-05
158	RAB17	2850349	0.95	5.11E-05
2015	CD200R3	2100292	0.95	5.16E-05
2096	MTRF1	4040286	0.84	5.26E-05
382	LAX1	2810722	0.95	5.79E-05
498	2310047O1	4830725	1.06	5.93E-05
19956	CTSA	610414	1.17	6.26E-05
7961	TK1	3450010	0.94	6.66E-05
73	A930025D0	2600253	0.95	6.88E-05
21426	MYBL2	3840121	1.08	7.60E-05
6349	DNAHC2	2900747	0.94	7.92E-05
3362	STARD8	4860445	1.25	8.14E-05
2800	TMTC1	6960440	1.06	8.71E-05
4779	GTSE1	1780719	1.07	8.84E-05
21561	OLFR1355	3180672	1.06	9.29E-05
20351	DNMT1	4480767	1.07	9.87E-05
11662	TMEM127	580392	1.14	1.01E-04
140	5730470L2	670243	1.05	1.01E-04
729	AMD2	4830768	1.10	1.02E-04
2553	TERF2	1190280	1.10	1.03E-04
7674	NSUN5	4180224	1.05	1.05E-04
2280	SRPX2	2190139	1.04	1.05E-04
10581	NDST3	3830450	0.95	1.10E-04
10655	TIRAP	4900102	0.96	1.10E-04
21370	1110012J1	2000739	1.14	1.11E-04
21374	CNOT6	2120037	1.05	1.12E-04
10222	ITIH5	2190762	0.96	1.16E-04
21728	CPA2	2970176	0.95	1.16E-04
5239	M6PR	2680577	0.93	1.22E-04
2470	ERC1	1090255	1.06	1.30E-04
12165	ABLIM1	2370592	0.95	1.34E-04
5342	TLN2	6420369	1.03	1.34E-04
12480	ANXA10	6650220	1.03	1.37E-04
8051	DNAJB8	4150053	0.95	1.38E-04
549	ZXDC	1050332	1.08	1.39E-04
15289	GALNT1	1980392	0.94	1.40E-04
8613	OLFR297	7150382	0.95	1.40E-04
19711	UCN3	2760112	0.96	1.42E-04
10751	KCNIP2	6620181	1.08	1.46E-04
10359	RGS4	4780128	1.11	1.55E-04
6242	ZFAND2B	1010195	0.86	1.56E-04
25428	V1RD7	6370088	1.05	1.57E-04
2205	PDK2	5890139	1.07	1.59E-04
25648	OLFR332	1010253	1.05	1.65E-04
23320	XRCC1	2070148	1.09	1.66E-04
1297	RGS9	670647	0.78	1.74E-04
23215	SIDT1	870156	1.18	1.78E-04
17944	ANKLE2	3360246	0.94	1.81E-04
7367	EXOSC9	5290195	0.94	1.81E-04
2046	MAS1	1570482	0.92	1.88E-04
3345	TMTC1	4220224	0.95	2.05E-04
6503	FBXO5	7400411	1.04	2.11E-04
859	CAR11	5670477	0.90	2.15E-04

Family 2

Group Rank	Gene	Probe	Fold Chang	P-Value
1164	NFE2L2	1110201	1.07	1.31E-08
11313	GTF2IRD1	4150341	1.09	3.51E-07
618	5033411D1	5310739	0.92	6.95E-07
2054	CLK4	7100519	1.09	7.62E-07
1283	NUP62	4070209	0.87	8.44E-07
17606	SLC37A1	6840301	0.95	1.13E-06
63	6330500D0	6620360	0.93	1.19E-06
1169	ZFP119	3120523	1.10	1.77E-06
911	TLX2	360324	0.94	1.80E-06
21726	GMIP	1940739	1.08	2.11E-06
1084	PHKA1	1980524	1.07	2.46E-06
21745	CTSA	4260564	0.95	2.64E-06
2488	FBXO32	580019	1.07	2.87E-06
18222	9430015G1	4880437	0.93	3.11E-06
1345	SORBS1	1690044	1.08	3.59E-06
16	TSPAN3	3130497	1.19	3.62E-06
21409	4930404N1	990441	0.94	4.37E-06
997	4921530G0	5900465	0.89	4.81E-06
5869	OLFR395	4570044	0.93	5.19E-06
1344	RAB34	3990131	1.13	5.33E-06
573	DCTN6	7610730	0.87	5.47E-06
404	HRK	2140278	1.11	7.15E-06
6826	A930008G1	2230341	1.08	8.33E-06
2975	TIMM23	4010022	0.95	8.52E-06
471	CDK9	6270139	0.93	9.57E-06
11817	SLC25A11	5960050	0.88	9.91E-06
2940	E130203B1	7510133	0.95	1.02E-05
4614	SLC22A14	3830538	1.07	1.10E-05
1561	CCL11	6060520	0.94	1.11E-05
22904	LOC100045	1230538	1.20	1.19E-05
1194	OLFR1500	4040114	0.95	1.36E-05
24779	SLC7A7	7320349	0.94	1.41E-05
10457	OLFR549	2230156	0.95	1.46E-05
150	OLFR893	3710196	0.95	1.48E-05
8494	PHLPP	110220	1.04	1.54E-05
983	OLFR869	4610220	0.94	1.68E-05
3712	CDGAP	4290369	0.94	1.72E-05
16512	PPEF2	1440762	1.07	1.84E-05
4919	4933405O2	7210746	1.04	1.88E-05
8028	HOXC11	6380093	1.07	2.03E-05
2542	SLC2A5	450020	0.93	2.10E-05
862	GABRA1	6590564	1.08	2.27E-05
1712	RPS19	3840465	1.07	2.40E-05
357	KEAP1	610397	0.88	2.48E-05
21627	D230010M	10754	0.95	2.48E-05
15684	MAB21L1	5260372	1.04	2.53E-05
4368	PCDHB5	6350634	0.93	2.60E-05
2469	LOC100044	3120180	0.92	2.70E-05
23860	MORC1	3170373	1.06	4.03E-05
3337	GPC3	3890500	0.94	4.11E-05
11383	HSD3B6	670372	0.94	4.20E-05
2719	SIDT2	4180086	0.93	4.20E-05
8370	TMC8	130681	1.06	4.46E-05
7831	TRAPPC2L	670014	1.13	4.46E-05
1243	CBLN2	2360181	0.93	4.53E-05
1654	EAR14	6900242	0.94	4.56E-05
139	FBXL20	3370450	0.96	4.61E-05
2355	LARP5	4860670	0.94	4.69E-05
12005	2900024C2	2360113	0.95	4.81E-05
14414	EGR4	4560681	0.86	4.91E-05
1271	PEX12	2060730	0.94	5.12E-05
15873	THOC1	2600161	1.11	5.50E-05
975	DNAJC15	1450066	1.22	5.62E-05
3897	KCNH4	5690678	0.95	5.69E-05
15415	RHOV	5090670	0.94	6.34E-05
2286	PLA2G2F	1980220	0.93	6.72E-05
1865	ADD1	3460592	0.86	6.81E-05
16381	4732456N1	6620427	1.06	7.00E-05
15910	GA_X5J8B7	4070221	1.05	7.36E-05
4563	ENPP7	2760228	0.93	7.67E-05
9796	HOXA3	5720347	0.94	7.90E-05
11799	SMO	2810440	1.07	7.99E-05
9236	MFN2	2450066	0.86	8.04E-05
295	RPS6KB1	2490397	0.92	8.65E-05
6	GSTM1	4540458	1.08	9.48E-05
12286	PMS2	4860768	0.94	9.69E-05
2827	SPNB2	1780309	1.08	1.00E-04
7210	KLC1	7330358	0.92	1.02E-04
19271	ZPBP2	3850292	0.93	1.04E-04
8089	APAF1	1990639	1.10	1.14E-04
361	ACP1	2750132	1.06	1.21E-04
23227	EGLN3	3290575	0.92	1.24E-04
1884	MKRN2	6900176	0.90	1.24E-04
11471	MAP3K13	3360021	1.05	1.26E-04
1919	POLM	2630438	0.96	1.28E-04
7991	ADAM26A	6370672	0.96	1.28E-04
2783	OLFR1209	5310767	1.05	1.28E-04

Family 3

Group Rank	Gene	Probe	Fold Chang	P-Value
763	ANGPT1	510735	0.89	1.11E-10
681	AQP7	6100703	0.92	1.24E-10
1088	F730047E0	5290608	0.90	9.18E-10
10816	EG435391	5890048	1.11	1.66E-09
339	TM4SF5	3710491	1.12	3.62E-09
13	FRYL	510059	1.11	3.78E-09
20504	BC026590	6900402	0.87	4.23E-09
9519	ADAT1	3130026	0.91	6.46E-09
2749	OLFR1231	4850438	0.94	8.50E-09
8458	GATA2	2940681	0.89	1.13E-08
23813	ZFP759	6550113	0.91	1.76E-08
2131	HAND2	7050468	0.92	1.95E-08
2328	5730446C1	6180431	1.12	2.15E-08
14902	AMBRA1	6040600	0.95	2.24E-08
8610	TRAF3IP1	6280703	0.92	2.36E-08
11143	PAPLN	2680672	1.10	2.70E-08
3827	ATP10D	4900743	0.90	3.38E-08
2560	GPR107	2510717	1.12	3.96E-08
422	STOML1	7210091	1.12	4.36E-08
24528	SEL1L2	6480279	0.92	4.70E-08
5863	NR5A1	5720288	0.91	5.06E-08
631	OLFR372	2940440	0.90	6.03E-08
2579	UIMC1	360400	0.92	6.21E-08
4539	MPDZ	7560703	0.90	6.93E-08
24630	BC002230	3780437	1.11	7.19E-08
1470	GIMAP7	670204	1.06	7.80E-08
205	OTOR	6840328	0.93	8.78E-08
10153	SUV39H2	7320438	0.93	8.87E-08
12	CD44	4880138	0.92	9.07E-08
1081	OXCT2B	5490639	0.91	1.00E-07
12281	OLFR572	2260538	0.95	1.07E-07
1562	MSI1	6590491	1.07	1.12E-07
7757	USP28	6110717	0.91	1.18E-07
5972	4933434I06	6110259	0.93	1.35E-07
192	LGALS12	4210176	1.07	1.35E-07
1435	MORC4	650431	0.94	1.38E-07
4163	BDNF	1570369	1.09	1.49E-07
7237	ZNHIT4	6560246	1.14	1.63E-07
69	CYP2D13	610333	0.92	1.69E-07
17160	LZIC	460148	1.11	2.24E-07
1128	ELMO1	5220554	1.11	2.49E-07
3560	LOC100039	6270408	1.08	2.57E-07
3662	IFNA1	4780072	0.92	2.68E-07
3417	GPR33	5050598	1.07	2.69E-07
4510	V1RC6	5340608	1.06	2.79E-07
1494	OLFR1318	4010367	0.92	2.87E-07
3882	MSC	1230746	1.10	2.88E-07
2525	EMP3	1070224	0.91	3.15E-07
799	RHBDL1	450056	0.89	3.32E-07
5932	OSBPL10	1690040	1.11	3.38E-07
1558	ATG3	20142	1.04	3.46E-07
751	NUDT3	150091	0.94	3.48E-07
3	LTA	2900474	1.07	3.83E-07
5725	9430034D1	50347	0.94	3.88E-07
2207	SMC1A	6330102	0.93	4.09E-07
23078	CRIPT	4860035	1.12	4.12E-07
7061	SFPI1	1740301	1.13	4.18E-07
9619	OLFR761	430189	0.92	4.21E-07
21210	1200016B1	7150719	0.94	4.45E-07
23926	OLFR994	6330754	0.94	4.68E-07
5215	NCF1	3520300	0.89	4.70E-07
10610	ASCC2	2570519	0.93	4.75E-07
11100	OLFR215	2970014	0.93	5.10E-07
10940	MTX2	6400204	1.08	5.40E-07
2305	TUBD1	270400	1.05	5.54E-07
161	SERPINA1A	360431	0.91	5.66E-07
1747	RNF151	3190192	0.92	5.68E-07
8646	BC043301	1070181	0.95	5.73E-07
7467	SNX3	1690528	1.10	5.91E-07
1771	GCSH	2120411	0.91	6.25E-07
14030	1700029F1	5820132	0.93	6.31E-07
22293	SFXN3	5290762	1.14	6.60E-07
1390	TAAR9	5700167	0.93	7.20E-07
5900	CLN8	2260446	1.10	7.20E-07
23200	KPNA2	7210291	0.94	7.28E-07
4689	KRT25	6590594	0.91	7.88E-07
1090	ZFP687	7040368	1.06	8.05E-07
12855	3830406C1	4230369	0.90	8.28E-07
998	SNAPC3	3370167	0.95	8.32E-07
11281	SS18	3460152	1.06	8.45E-07
12921	B230118H0	830026	0.94	8.69E-07
25000	OLFR438	5090091	0.90	8.73E-07
13983	A330021E2	4610047	1.06	9.99E-07
19798	GBP2	6860600	1.07	1.01E-06
1462	B4GALT1	6840468	0.94	1.08E-06
783	IL6ST	5900575	0.89	1.12E-06
5655	PSG27	4010400	1.07	1.15E-06

Family 4

Group Rank	Gene	Probe	Fold Chang	P-Value
1999	NUP133	1010224	1.09	2.11E-08
705	CYB561D2	5960017	0.91	2.56E-07
1961	OLFR954	2100519	1.08	2.61E-07
16095	SAMD12	1230402	1.09	4.21E-07
18386	GPR75	2750551	0.95	5.30E-07
21	ZC3H18	7380382	1.09	7.51E-07
3161	ARL3	2450463	1.15	8.83E-07
8353	DUSP3	1090575	0.93	9.87E-07
8137	9930039A1	5360100	1.06	2.05E-06
871	CIC	7560100	1.13	3.04E-06
4293	CAMK2A	3060288	1.23	3.28E-06
23676	KLK13	2450364	1.07	4.94E-06
3026	ZFP367	5900605	0.95	5.80E-06
4070	1300014I06	4880132	0.94	6.48E-06
23822	HK1	4150397	0.90	6.99E-06
785	P2RY2	7610364	1.08	7.21E-06
2703	MRPL30	580161	1.14	9.20E-06
18647	COL17A1	6770369	1.06	9.63E-06
218	SIGLEC1	4570458	1.06	1.07E-05
15465	USP15	5860377	0.95	1.23E-05
1599	GNGT2	7330379	1.08	1.48E-05
190	ABCG5	4290400	0.95	1.50E-05
1652	GDPD5	2260746	0.90	1.61E-05
4903	MON1A	5570209	1.10	2.08E-05
1332	4932411G1	580370	0.95	2.16E-05
11817	SLC25A11	5960050	0.89	2.25E-05
1817	POM121	7210280	1.07	2.29E-05
10538	AI837181	4010722	0.85	2.49E-05
29	LARP4	6400025	0.94	2.52E-05
1806	MFAP2	3780358	0.93	2.66E-05
1095	CHIC2	4540154	1.16	2.92E-05
7727	PABPC4	4480674	1.07	3.40E-05
148	OTX1	4730593	0.88	3.40E-05
233	LRRC14	650403	0.92	3.74E-05
6562	ERDR1	4880187	0.89	3.81E-05
570	ARID5A	3180736	0.95	4.60E-05
14483	B2M	5570253	1.12	4.68E-05
7539	IMPAD1	3370291	0.92	4.71E-05
437	GLI3	6040707	1.05	4.87E-05
289	PEX6	2340537	0.85	4.98E-05
10973	GMNN	2970747	1.06	5.31E-05
4646	USP53	510400	1.07	5.56E-05
1886	MFSD8	7150670	1.07	5.57E-05
3696	AZI2	1780681	1.07	5.69E-05
9687	DLEU7	130672	1.09	6.69E-05
200	AMELX	6840246	0.92	6.79E-05
708	RBPJ	4390056	1.05	6.85E-05
1412	AI597468	6760671	0.90	6.89E-05
4215	RP2H	6840324	0.93	7.14E-05
2067	DEFCR-RS1	6520059	1.07	7.21E-05
8172	BXDC5	4480220	0.95	7.23E-05
1350	C230078M	2260739	0.95	7.36E-05
5863	NR5A1	5720288	0.92	7.63E-05
374	OLFR402	3290717	0.92	7.66E-05
8263	OLFR1408	6420161	1.07	8.19E-05
17258	RHOD	1570154	1.04	8.60E-05
20938	MSH6	7610338	0.92	9.74E-05
2038	POLS	7510743	0.89	1.01E-04
1011	D0H4S114	7040243	0.80	1.02E-04
1110	EAR10	7560228	0.96	1.08E-04
11423	TRP53BP2	4830347	1.06	1.14E-04
8612	PHF17	4150292	0.91	1.16E-04
21519	HAP1	1580309	0.84	1.16E-04
18079	ST8SIA4	780500	1.05	1.18E-04
7282	2310009E0	6660133	0.95	1.19E-04
1385	APRT	2480551	0.90	1.20E-04
3709	MYL7	1010201	0.92	1.22E-04
20675	RHOT2	1710520	1.09	1.25E-04
2935	PIN1	5390100	0.94	1.28E-04
15319	RDH1	50367	0.96	1.30E-04
7631	1700052N1	4200494	1.07	1.30E-04
3188	V1RB4	940240	1.06	1.36E-04
15085	TPH1	130066	0.95	1.38E-04
19892	V1RC18	5360010	0.96	1.38E-04
3735	2510010F1	6110474	1.10	1.39E-04
6877	PPP3R1	2600754	1.11	1.40E-04
2047	AP3M1	4570386	1.06	1.40E-04
14157	PTPRO	1980100	0.94	1.61E-04
2282	BC006662	2070367	1.14	1.64E-04
17288	ERN1	3520128	1.05	1.70E-04
11883	ATG4D	2230020	1.05	1.83E-04
56	SBNO2	6220037	1.05	1.83E-04
774	IFNAB	240669	1.07	1.84E-04
72	FECH	3420291	1.06	1.86E-04
25481	LOC100041	460754	1.03	1.91E-04
19449	KCTD8	2490692	0.94	2.03E-04
3553	MEIS3	2650482	1.11	2.09E-04

Family 5

Group Rank	Gene	Probe	Fold Chang	P-Value
746	UGT1A2	1740148	0.93	1.30E-08
1020	VCAN	360747	1.07	2.00E-08
11782	PDRG1	7400519	0.85	4.77E-08
20662	UTP6	2120451	1.09	1.13E-07
5907	MGST2	7330292	1.06	1.53E-07
4053	PYGM	6370484	1.08	2.56E-07
4084	PSMC1	6420072	1.13	3.03E-07
4254	TTLL13	580278	0.93	5.42E-07
1210	2810408M0	4860176	0.93	6.65E-07
19830	A530088H0	1070672	1.06	1.02E-06
2901	LRRC48	4250427	0.92	1.13E-06
4718	OLFR8	2320722	1.07	1.21E-06
2323	DNAJB4	1010653	1.04	1.43E-06
3661	V1RH21	6520433	0.95	1.94E-06
18068	4930547N1	5490181	0.94	1.98E-06
741	CAV3	7650332	0.94	2.85E-06
18621	NCAM1	3390731	1.12	3.19E-06
1258	SLC25A46	6400121	0.91	3.34E-06
20406	ANP32A	1090142	1.15	3.43E-06
5615	ATG2A	2030152	0.91	3.63E-06
21046	CYP2C68	5700376	0.94	5.25E-06
835	PLOD2	3890465	0.93	6.05E-06
2577	OLFR1508	4050671	1.05	6.23E-06
2250	HIST1H2AF	4250711	0.82	6.32E-06
15973	GNPNAT1	6650474	1.05	6.36E-06
533	TGFBR1	2810167	0.95	7.17E-06
24151	A730018C1	5490750	1.09	7.19E-06
13	FRYL	510059	1.06	8.37E-06
436	HTR3B	5890762	0.94	9.43E-06
12563	MRPS33	990630	0.86	1.21E-05
170	IDUA	4490598	0.95	1.29E-05
735	MCFD2	1170397	0.95	1.33E-05
6010	NFIC	290041	0.88	1.33E-05
2158	MFAP1B	4390075	1.11	1.33E-05
3058	ITGA3	2360703	1.10	1.34E-05
7965	LOC100047	5220440	0.87	1.36E-05
8426	STK19	4810471	0.92	1.37E-05
7834	RAB6	1090279	0.93	1.46E-05
2014	CHI3L3	2570240	0.94	1.46E-05
282	CREB3L2	4010047	1.05	1.62E-05
11613	PSEN2	990682	1.07	1.66E-05
3268	TMOD2	6290767	0.95	1.70E-05
4080	APOC4	940451	0.92	1.76E-05
3389	LACTB2	520609	0.94	1.82E-05
1225	SMGC	6480451	0.94	1.90E-05
12087	CHI3L3	3870242	0.93	1.94E-05
5458	ADCY9	3310017	1.09	1.95E-05
18305	GRN	2000246	1.11	2.10E-05
14240	DERL2	3800097	1.07	2.13E-05
5597	PIGW	6020678	1.05	2.20E-05
22056	MTMR14	1770577	0.96	2.26E-05
298	OSMR	5910220	1.06	2.30E-05
1855	HOXD12	4280301	0.95	2.32E-05
1139	PLA2G7	4120255	1.07	2.55E-05
19627	TMEM57	4230019	1.15	2.85E-05
2655	TRPM7	3610523	0.96	2.91E-05
1699	TPP1	3710402	1.11	3.17E-05
6167	OLFR211	3440491	0.93	3.26E-05
17842	PRPF40B	830563	1.07	3.33E-05
720	SULT2B1	6110181	1.05	3.36E-05
22073	PSMC4	5050563	1.08	3.48E-05
13283	4921521F2	7160592	1.05	3.68E-05
2994	EG432743	1430465	0.96	3.92E-05
12937	9130401M0	1110114	0.95	4.05E-05
13328	MRPL40	2680164	0.89	4.12E-05
11384	EEF1A2	3180086	0.88	4.27E-05
2612	UBXD3	6330538	0.96	4.52E-05
17646	5830426I05	6550348	1.06	4.55E-05
13624	OLFR320	1660546	1.06	5.28E-05
11506	TNFAIP3	430523	0.94	5.37E-05
16043	PHOSPHO2	4150551	0.90	5.37E-05
761	TCTEX1D1	3800204	1.07	5.52E-05
11166	ARPP21	2630370	1.18	5.97E-05
3016	NUBPL	4480255	0.94	6.04E-05
579	PDGFRL	3890066	0.94	6.56E-05
2824	OTT	6040368	0.93	6.65E-05
10006	MDM2	3310243	0.90	6.77E-05
18320	RASGRF1	6270465	1.21	6.99E-05
1501	LOC100047	7510609	0.95	7.16E-05
380	PIP5KL1	2970201	0.93	7.42E-05
5150	HCFC1R1	1030768	0.96	7.49E-05
6999	OLFR776	3360678	0.95	7.67E-05
326	KCNK9	150092	1.06	7.70E-05
164	IGJ	4250162	0.93	7.83E-05
1165	APH1B	6220612	1.06	7.88E-05
20185	RTKN	3170543	0.94	7.89E-05
5012	TMEM70	3420347	0.88	8.26E-05

Family 6

Group Rank	Gene	Probe	Fold Chang	P-Value
630	SNX15	1410136	1.10	1.61E-09
1596	CLCNKA	4760767	0.93	1.95E-09
611	NGLY1	430279	1.07	1.28E-08
244	ANP32A	2600343	1.11	1.49E-08
376	FPR3	510048	0.91	1.93E-08
2391	H28	3840360	0.93	2.92E-08
11389	OLFR398	4730343	1.07	3.02E-08
7293	MAP2K6	1240300	0.95	4.30E-08
68	USP16	1780563	1.11	4.60E-08
1851	SHOX2	3360092	1.07	8.33E-08
2222	1110059P0	5910193	1.07	8.38E-08
7961	TK1	3450010	0.91	8.48E-08
2229	GPD1	3710019	1.12	1.56E-07
1738	GCNT2	620398	1.11	1.82E-07
12294	NHSL1	50341	0.92	1.93E-07
1785	POU2F1	650678	0.93	2.00E-07
5922	HNRPDL	3190092	1.10	2.25E-07
14674	IFNA12	3890278	0.93	2.29E-07
10188	LOC100048	6660152	1.10	2.43E-07
1550	EG208426	6760471	1.09	2.54E-07
428	LASS4	2600437	0.91	2.65E-07
21835	KCNG4	1030634	0.94	3.44E-07
2323	DNAJB4	1010653	1.07	4.19E-07
22821	BMX	6560239	0.91	4.47E-07
5182	SEMA3G	4560129	0.94	4.92E-07
3279	8030451F1	5670735	1.07	5.39E-07
922	IMPG1	4490524	0.93	5.56E-07
22	ATG5	3850450	0.90	5.64E-07
3017	NDUFB11	5670364	1.08	6.31E-07
13896	0610010D2	2850424	0.92	6.50E-07
2767	2300005B0	6270288	1.06	6.66E-07
17850	PHLDB1	7160021	1.11	6.82E-07
8452	BC022224	2710114	1.12	6.86E-07
436	HTR3B	5890762	0.91	7.40E-07
4445	OLFR473	3450554	1.07	8.50E-07
184	AW111922	4390630	1.07	9.28E-07
20196	EG214321	580324	1.06	9.73E-07
805	MRPL48	7210398	1.17	9.80E-07
20091	PPP1R16A	10259	0.91	1.01E-06
10330	KCTD10	2060612	0.94	1.11E-06
25269	ADO	1050521	1.07	1.13E-06
71	KIF4	6660050	0.91	1.21E-06
454	CRYZ	990594	1.12	1.24E-06
15553	4833442J1	50136	0.93	1.25E-06
8002	ASPH	580086	1.07	1.36E-06
19720	LPCAT2	6020451	1.06	1.39E-06
19735	MTA1	5310427	0.91	1.48E-06
4056	A430090L1	6860202	1.06	1.54E-06
4113	CML2	6580092	0.93	1.56E-06
14023	UNC84A	1940100	1.07	1.58E-06
806	OLFR1272	5390059	0.95	1.59E-06
13170	SEMA6C	2360358	0.90	1.68E-06
14607	ATG4C	6040050	1.12	1.69E-06
459	NARG2	7560097	1.08	1.80E-06
2109	JMY	5820707	0.94	1.82E-06
139	FBXL20	3370450	0.95	1.88E-06
23784	RAGE	3190372	1.09	1.97E-06
6836	ZDHHC6	5570088	0.89	2.06E-06
21389	PAFAH2	4670167	1.05	2.16E-06
17829	CD44	1660070	0.95	2.24E-06
16502	2410042D2	2760131	1.13	2.33E-06
1381	ZFP40	1230400	0.93	2.39E-06
841	ELMO1	2230097	0.92	2.42E-06
127	KCNK2	7650669	1.09	2.45E-06
5424	ACAD10	5570379	1.06	2.63E-06
2142	EPHX2	3520491	0.89	2.70E-06
2693	2410127L1	4180201	0.92	2.76E-06
8406	C730027P0	5270364	1.08	2.81E-06
1702	BC024814	150561	0.89	2.82E-06
15531	EG229862	3290152	1.07	3.12E-06
13943	IL7R	1660504	1.06	3.14E-06
6108	FCGR2B	5700220	1.08	3.27E-06
3177	EIF5A	4230563	1.10	3.29E-06
241	RASGRF2	6580754	1.09	3.41E-06
2154	SCGB3A1	4810017	0.94	3.44E-06
113	FERT2	4480482	0.93	3.45E-06
9318	EMR4	5310491	0.93	3.45E-06
1085	TMEM168	6860689	1.05	3.45E-06
1245	SOX21	3130593	1.07	3.47E-06
23745	UBL4B	6900279	0.95	3.50E-06
3629	WSCD2	360619	1.09	3.54E-06
5778	V1RI9	2260372	0.94	3.54E-06
2181	SMAD6	1450672	1.07	3.59E-06
10652	OLFR948	6900184	1.05	3.79E-06
9594	OLFR52	10484	1.07	3.80E-06
23003	CCDC60	5870072	0.94	3.86E-06
3611	GPR113	1690026	0.94	4.02E-06

19112	TRPV2	1690382	1.07	2.21E-04
8967	P2RX5	6330451	0.95	2.27E-04
13626	1700019G1	4290341	0.96	2.34E-04
6194	RIC3	270541	0.89	2.36E-04
19738	D4WSU53E	2030441	1.05	2.38E-04
6233	PCDHGB2	6840349	1.08	2.38E-04
3910	CCRN4L	4250689	1.14	2.40E-04
862	GABRA1	6590564	1.05	2.47E-04
19918	A030010K2	7320386	1.05	2.47E-04
15399	RAB4A	3440041	1.07	2.47E-04
22544	EG330602	6370142	1.04	2.49E-04
16733	HPD	2360528	1.05	2.56E-04
675	EBF1	3990170	1.07	2.65E-04
10921	ATP1A1	5290630	1.19	2.65E-04
20476	EXOSC5	5690408	0.92	2.69E-04
10645	HIST1H2BA	3940441	0.95	2.74E-04
23223	RAF1	770327	0.94	2.84E-04
420	CALR3	2000722	1.05	3.03E-04
18098	DGKQ	1710474	1.06	3.08E-04
1924	AEBP2	610020	1.05	3.17E-04
1376	LIP1	3840066	1.08	3.19E-04
25220	CIRBP	6980576	0.87	3.32E-04
9673	NRGN	4570189	1.25	3.34E-04
6866	VANGL2	5310195	0.96	3.44E-04
589	LOC100044	2650433	0.96	3.48E-04
12235	PCDHGB6	7320041	1.04	3.54E-04
15412	ACSS3	2370307	1.04	3.58E-04
21	ZC3H18	7380382	1.11	3.61E-04
8045	ANKLE2	1050709	0.93	3.76E-04
20023	COMMD5	3520176	0.93	3.78E-04
1576	A530032D1	1410600	0.96	3.82E-04
6657	DMKN	50521	0.90	3.84E-04
31	IER5L	4900053	0.84	3.95E-04
6823	BZW2	4480546	0.94	4.00E-04
9422	PCDH17	6270411	0.90	4.02E-04
6057	RRAGB	2760465	0.91	4.08E-04
51	2410018G2	6280408	0.94	4.18E-04
943	GSK3A	1710630	1.08	4.20E-04
10182	INSIG1	4920369	0.89	4.22E-04
22598	SUPT5H	7400546	1.17	4.28E-04
3172	PRSS29	2470762	0.95	4.29E-04
21299	SERPINB3B	1090286	0.95	4.30E-04
1006	FXYD6	6550309	0.84	4.36E-04
7216	MYOCD	4830711	1.04	4.38E-04
3121	TM2D2	1240279	0.89	4.44E-04
566	SUMF2	2470279	0.92	4.56E-04
5062	DEXI	2470044	0.91	4.58E-04
4069	ABCF2	1500110	1.10	4.73E-04
19258	SCARB2	3310564	1.10	4.75E-04
1764	TEK	2360519	1.10	4.78E-04
1200	OLFR313	3940376	0.95	4.85E-04
21245	LBXCOR1	4780041	0.94	4.86E-04
29	LARP4	6400025	0.95	4.88E-04
15265	LOC546723	1660014	0.96	4.90E-04
22770	1700016G0	5220307	0.95	4.98E-04
7817	COPE	6040433	0.91	4.99E-04
15887	TXK	7150561	1.04	0.001
18952	RAB8A	1240324	0.93	0.001
20105	MRPS22	7050128	0.88	0.001
16703	SFRS4	5910424	1.12	0.001
22972	OLFR335	5260224	1.05	0.001
9503	ZEB2	2850703	1.17	0.001
1804	GM1008	630397	1.05	0.001
15193	ZFP458	5670471	0.95	0.001
12303	LOC100044	5310408	1.04	0.001
24592	TTLL1	7100180	0.94	0.001
9499	GM1070	5310296	1.06	0.001
3795	LMBR1	2360484	1.07	0.001
23043	MGC10709	6960474	1.04	0.001
1899	ITGA2B	6520221	1.07	0.001
13742	CORO1B	3840092	0.89	0.001
10614	LOC100045	50576	0.97	0.001
5008	ANKZF1	2340750	1.05	0.001
4422	2010308M(	5090609	1.05	0.001
7277	XKR6	4880382	1.05	0.001
4151	4930504O1	1230008	0.95	0.001
21272	DDX47	6480682	0.92	0.001
23207	PNPLA6	6250414	1.07	0.001
16491	PDE1A	1820341	1.16	0.001
4934	TIMD4	3840367	0.95	0.001
219	IL7R	1780079	0.95	0.001
11976	NR0B2	5420398	0.95	0.001
12289	EGFLAM	1340670	1.04	0.001
6206	ISG20L1	4290554	0.95	0.001
203	INPPL1	1990440	1.08	0.001
400	APEX1	6250138	0.95	0.001
4406	TBC1D2	5550482	1.04	0.001
3876	BICD2	5810181	1.06	0.001
21966	ICAM4	6280092	0.94	0.001

9881	CNTN3	4010463	0.93	1.33E-04
558	C030046E1	10504	0.94	1.36E-04
6250	SON	3290132	1.08	1.36E-04
6512	2310016C1	7050328	1.07	1.39E-04
22276	RTN4	6520138	0.92	1.39E-04
5871	ASB3	2370577	0.94	1.40E-04
4049	KIF3C	4670487	0.88	1.44E-04
330	KCNT1	460121	1.07	1.46E-04
16301	CASP9	840411	0.95	1.47E-04
16252	AP3D1	6770403	0.87	1.52E-04
1282	CTSF	5910452	1.08	1.57E-04
1060	SLC24A5	6130242	0.91	1.61E-04
8112	ING3	2360138	0.92	1.63E-04
815	NPNT	1940543	0.95	1.65E-04
6285	H2-K1	6280026	0.96	1.66E-04
5090	RESP18	2490279	0.85	1.69E-04
6615	OLFR519	6960546	1.07	1.74E-04
7067	PCDH7	1450403	1.04	1.81E-04
5364	C230052I12	2140403	1.08	1.87E-04
1424	RALBP1	2650370	0.89	1.90E-04
25011	RGS5	3170053	1.09	1.90E-04
6728	HIST1H2BB	1410195	1.06	2.01E-04
432	ZFP280C	2120112	0.94	2.01E-04
426	DTX4	130102	0.92	2.06E-04
8049	KATNAL1	6350437	0.93	2.09E-04
5062	DEXI	2470044	0.94	2.10E-04
7374	AMPD3	540446	1.06	2.14E-04
9307	SPSB1	5700066	0.92	2.17E-04
5203	HLTF	4610326	0.94	2.17E-04
7917	MTMR11	2970609	1.08	2.32E-04
752	NFATC2	5290255	0.95	2.38E-04
2187	CEBPA	7380400	0.96	2.40E-04
6837	OLFR458	6020132	1.05	2.46E-04
3601	BC031901	1440397	0.95	2.51E-04
41	XKR8	770133	1.09	2.51E-04
6494	VSX2	1110064	0.93	2.53E-04
2825	DLST	2480482	0.88	2.53E-04
21523	ADCY1	7560367	0.85	2.57E-04
25495	GTF3C1	1070070	0.89	2.59E-04
2130	LOC100038	2570707	1.06	2.60E-04
4107	ADRBK2	4040474	1.06	2.71E-04
613	MASTL	2970044	1.04	2.74E-04
12182	TM4SF1	7610477	0.97	2.74E-04
16147	AKT3	50603	0.93	2.77E-04
23508	5730502D1	4210541	0.93	2.82E-04
10508	COX4NB	3440280	1.08	2.85E-04
16907	ETS1	5340630	1.06	2.85E-04
10518	ZFP622	6580437	0.96	2.86E-04
3098	MOSPD1	990349	1.07	2.91E-04
2815	ARHGAP1	160528	0.89	2.91E-04
8940	TMTC4	1660450	1.05	2.93E-04
16292	RNF125	4210463	0.95	3.05E-04
15000	CSF3	7200762	0.96	3.05E-04
18118	FRAS1	3870148	0.95	3.07E-04
5119	RECK	7320239	1.06	3.07E-04
91	ARF4	5260730	1.07	3.08E-04
16373	RAB40B	5270431	0.95	3.10E-04
20394	GSTO1	1010435	0.92	3.14E-04
2911	SPNS1	5220601	0.93	3.14E-04
12369	MBD1	5810474	1.06	3.27E-04
730	PVR	4850039	1.09	3.35E-04
18833	PROP1	3800047	1.04	3.37E-04
1845	TNFRSF13B	5420735	0.92	3.39E-04
1360	NUDT12	3420692	1.05	3.73E-04
1697	TNPO3	2470768	1.05	3.75E-04
10236	MTAP7D1	1110167	1.04	3.81E-04
2508	LOC100048	2340181	1.05	3.83E-04
10972	RNASEK	4760482	0.90	3.87E-04
6905	4833413D0	4890398	0.96	3.95E-04
3117	DHCR24	2100162	0.95	3.99E-04
10120	CHRM1	50470	1.04	4.01E-04
26	TNFSF11	2480255	1.05	4.03E-04
22415	GTF2IRD1	5550356	1.09	4.04E-04
10643	RPL39L	1070367	1.05	4.13E-04
6692	PRRT1	5310736	0.93	4.13E-04
6064	ADAMTS13	5860373	0.93	4.14E-04
6972	RHBDD2	5260142	0.95	4.14E-04
3959	GSC	4780019	1.07	4.25E-04
2330	USP4	4540010	0.93	4.34E-04
2720	ARHGEF10L	3840689	1.05	4.48E-04
1996	CEACAM19	6420278	1.04	4.48E-04
18526	ENTPD7	4210164	0.95	4.65E-04
2676	HSD17B3	4610592	0.94	4.66E-04
21927	ZFP329	990039	0.94	4.69E-04
4999	GEM	6840274	0.90	4.74E-04
10798	PCAF	1090326	1.04	4.88E-04
5098	APRIN	1090669	1.07	4.89E-04
4251	ANG	5360537	0.94	4.93E-04
16590	TRAM1	4480402	0.91	5.03E-04

9942	FLII	4220050	1.07	1.15E-06
16084	PSMD11	5870291	0.94	1.18E-06
695	UGT2B37	5860463	1.07	1.18E-06
22822	ADD3	3450040	0.87	1.19E-06
1822	VRK1	6420379	0.93	1.26E-06
7597	4921511H0	520717	1.06	1.30E-06
2739	SDCCAG8	5390593	1.11	1.34E-06
383	1700123K0	840634	0.92	1.43E-06
2242	OLFR362	6860670	0.92	1.53E-06
2186	RBM42	2190528	0.87	1.56E-06
261	ZIC4	5890528	0.91	1.58E-06
657	F8	6200551	1.08	1.67E-06
345	TRABD	2690240	1.11	1.71E-06
6017	HOMER1	1510672	1.06	1.71E-06
21592	INSIG2	7510356	1.12	1.71E-06
3800	SEC14L3	3990138	0.94	1.73E-06
179	EG232801	6590379	0.94	1.76E-06
18820	TMEM17	2970537	1.10	1.78E-06
22646	GRWD1	990129	0.91	1.93E-06
8682	CDC14B	10255	0.91	1.94E-06
18658	ZP1	1090543	1.09	1.94E-06
4955	SNX14	1740577	0.94	1.95E-06
13473	ANKRD24	4260452	1.11	2.02E-06
3304	YTHDF3	5700286	0.94	2.10E-06
360	SLC35C1	3390328	0.92	2.13E-06
351	HS6ST1	6350402	0.88	2.15E-06
210	MTIF2	6940017	0.93	2.25E-06
504	DCUN1D3	1070438	1.09	2.27E-06
494	SAMSN1	6020400	1.11	2.30E-06
5943	KLHL5	6380373	0.92	2.35E-06
1519	TALDO1	7150121	1.08	2.38E-06
124	PTP4A2	5820646	1.08	2.91E-06
16272	2810410C1	5490598	0.94	2.96E-06
15912	STAR	6110332	1.04	3.07E-06
13883	ASPH	1510564	1.07	3.07E-06
142	RBM47	6420372	0.95	3.16E-06
7893	PIGB	6760192	0.93	3.30E-06
25637	IRAK4	5220452	0.94	3.38E-06
21420	TIAM1	5270452	0.79	3.48E-06
23107	2310031L1	4120437	1.05	3.79E-06
3437	ACSL3	5560615	0.86	3.92E-06
5024	CALD1	4210431	1.07	4.04E-06
1100	CDH16	6220440	1.06	4.11E-06
2526	RAB10	3450450	1.07	4.18E-06
10575	HEATR1	1070368	1.08	4.40E-06
5598	ASB8	270309	0.84	4.44E-06
8627	ZCCHC9	3120762	1.12	4.50E-06
16905	TGFB3	2510500	0.93	4.60E-06
1525	LUC7L	3890192	1.05	4.80E-06
5608	PSENEN	7510332	0.83	4.98E-06
3711	HMGA2	4230719	1.08	5.00E-06
47	ZADH2	4230730	1.10	5.03E-06
2675	BC055111	990095	0.93	5.05E-06
5280	SERPINH1	4850221	0.91	5.56E-06
21109	INPP5B	5220373	1.05	5.62E-06
8044	UCP2	4920594	1.08	5.68E-06
17068	2610020H0	4760095	1.07	5.77E-06
1830	ZFP454	2710397	1.06	5.97E-06
3201	SLC15A2	4120750	1.07	5.99E-06
13391	SERPINB1C	7510296	0.93	6.07E-06
13405	CWC15	2070730	1.06	6.34E-06
3098	MOSPD1	990349	0.91	6.44E-06
651	MSTO1	4860402	1.10	6.78E-06
1006	FXYD6	6550309	0.82	6.79E-06
22018	C230095G0	6450474	1.05	6.82E-06
9724	NSUN2	2650504	0.93	6.90E-06
23758	KNTC1	1410070	1.07	6.94E-06
9910	A730008L0	150059	1.08	7.31E-06
22590	SSXB2	5700743	0.93	7.34E-06
16361	OLFR1013	4060358	1.05	7.58E-06
3658	S100A4	5720646	0.94	7.69E-06
18015	TREML1	6400307	1.06	7.78E-06
12691	ASL	1430139	0.93	7.79E-06
2958	XRN1	450097	0.94	7.86E-06
16810	0610007P2	1070170	1.15	7.94E-06
415	ITGB1BP2	6220551	1.07	7.98E-06
2093	PPP2CB	6380484	1.10	8.02E-06
11472	HOXD11	6200669	1.06	8.14E-06
9095	TMEM184A	3780113	1.06	8.18E-06
185	WDR31	2340338	1.07	8.18E-06
4604	CNBP2	2490554	0.90	8.31E-06
95	SERHL	5360041	0.94	8.61E-06
7006	GPR27	6200253	0.93	8.65E-06
3171	KRT32	1500762	1.08	8.78E-06
137	PKHD1	4860333	0.93	8.86E-06
7486	PSMA3	4850181	0.93	8.88E-06
24247	SPEER4F	7550598	0.94	9.27E-06
232	OLFR482	3800292	0.95	9.46E-06
5200	TMED8	3140241	0.93	9.78E-06

620	TSPYL1	3520291	0.94	2.23E-04
18670	FNTA	6620743	1.04	2.27E-04
1667	TXNDC15	3890176	0.87	2.27E-04
7743	OLFR733	4010139	0.94	2.49E-04
16383	ATP11C	5290379	1.04	2.51E-04
18760	MEI1	2680682	0.96	2.54E-04
16839	SEC22B	1240497	1.13	2.56E-04
20183	OLFR1389	4560576	1.06	2.70E-04
23747	DUSP19	6180372	1.10	2.75E-04
14769	COPS5	2970563	0.92	2.76E-04
16518	SEC11C	5130497	1.09	2.78E-04
2743	IL12B	4390600	0.95	2.83E-04
4957	OGN	450537	1.04	2.86E-04
16996	CHRNB4	1980291	0.96	2.94E-04
7840	SGIP1	3310474	0.92	2.98E-04
1791	MYH9	4150541	0.89	3.02E-04
13309	LIF	5050301	1.06	3.10E-04
82	MRPL13	3400424	0.95	3.11E-04
116	ATP5G3	1580471	1.10	3.21E-04
8639	ATP2B2	3170148	0.92	3.27E-04
459	NARG2	7560097	1.05	3.29E-04
14271	EBI3	2070537	1.05	3.30E-04
9212	PAM	6290088	0.96	3.33E-04
5895	RHOX9	650112	0.95	3.39E-04
4958	PRKCZ	5820681	1.12	3.48E-04
20006	KLHDC9	5050138	0.94	3.55E-04
3645	TPT1	3710605	1.05	3.62E-04
4426	2610101N1	3830707	0.92	3.66E-04
9608	TDG	4780671	1.07	3.68E-04
5788	CDYL	5860386	1.05	3.69E-04
2644	ACPP	7320220	1.07	3.74E-04
3397	MDM1	4280669	0.95	3.81E-04
20697	ASNSD1	3840431	0.96	3.82E-04
11578	1700065O1	650400	1.08	3.84E-04
2818	4732418C0	2680474	0.94	3.86E-04
7546	DNAJC19	6330242	1.07	3.92E-04
7750	SVIL	4210678	1.05	4.03E-04
2408	TOMM70A	1450112	1.08	4.13E-04
354	AOF1	5870192	0.94	4.13E-04
3914	CRIPT	1090450	0.95	4.20E-04
4888	BC026585	2900661	1.09	4.26E-04
6879	OLFR891	540671	1.06	4.27E-04
1339	CDV3	3360452	0.86	4.30E-04
375	SEMA6B	2600561	0.92	4.32E-04
118	LOC100048	990288	0.93	4.32E-04
3301	GHRL	2470619	1.05	4.37E-04
16769	CYHR1	5670373	0.95	4.50E-04
5722	GABRD	6510170	1.15	4.52E-04
1297	RGS9	670647	0.81	4.59E-04
17824	BC031781	2340438	0.92	4.63E-04
12819	V1RD9	4560367	1.05	4.64E-04
464	ZFP157	2340041	0.92	4.64E-04
165	CLEC16A	5090739	1.06	4.69E-04
25095	PIGK	4070564	0.96	4.69E-04
1488	OLFR600	610072	0.96	4.87E-04
15267	FJX1	5670170	0.90	4.92E-04
5505	S100A3	6270228	0.93	5.07E-04
11273	SYN1	7650196	0.87	5.26E-04
23793	NRL	130167	0.95	5.28E-04
5888	EBF2	940148	0.96	5.32E-04
11978	PTPDC1	3360040	1.05	5.32E-04
15914	NDUFS2	4180379	1.06	5.36E-04
25558	HDAC9	4540369	0.97	5.37E-04
19447	PSG22	2710280	0.95	5.41E-04
976	TMEM126E	4540619	1.15	5.41E-04
257	PECAM1	1030739	1.09	5.49E-04
9177	FDX1	6420538	0.94	5.61E-04
5392	CCKBR	7510521	0.85	5.61E-04
285	4930596D0	6550403	1.07	5.65E-04
4248	LOC100047	1090358	0.95	5.68E-04
12426	ABCB8	3360181	0.94	5.74E-04
2907	FKBPL	5720040	1.07	5.92E-04
6347	DOLPP1	5670167	1.07	5.96E-04
16405	2510006D1	4670092	1.11	6.03E-04
1171	TBCA	4560047	1.13	6.04E-04
2403	OGFOD2	6510050	1.07	6.07E-04
2666	REN1	1050204	0.95	6.19E-04
2736	P2RY6	3710403	1.07	6.29E-04
18621	NCAM1	3390731	0.93	6.61E-04
7856	LGALS4	6450278	0.95	6.66E-04
12966	TMEM60	6060576	1.12	6.69E-04
18750	WFDC10	6480184	0.95	6.81E-04
35	VTCN1	3440253	0.95	6.89E-04
4810	CEACAM1	990364	1.06	6.93E-04
23998	BC013712	770273	1.05	6.93E-04
10639	4933421E1	840484	1.11	7.01E-04
1047	FOXP4	10358	1.05	7.25E-04
1664	COMMD6	2710653	1.14	7.25E-04
4065	RNF219	1430576	1.06	7.29E-04

10256	TRAPPC2	4220242	0.95	8.41E-05
24754	AR	7000292	1.09	8.50E-05
4958	PRKCZ	5820681	0.92	8.79E-05
25439	ARL8A	1110382	1.19	8.88E-05
21437	0610012H0	4260014	0.96	8.92E-05
10545	ZFP263	1440270	1.09	9.29E-05
9658	2400003C1	50170	1.11	9.56E-05
654	SLC29A1	5870687	1.09	9.84E-05
23674	MPV17	2360333	0.88	9.86E-05
1183	SYPL	2060669	1.12	9.88E-05
186	MAP6D1	4060468	0.95	1.01E-04
15891	TBC1D1	3940142	0.95	1.02E-04
3157	NELF	620754	0.86	1.04E-04
4520	OLFR1167	510202	1.05	1.05E-04
4819	ADAM7	6220593	0.95	1.06E-04
2078	NOL11	4280564	1.11	1.10E-04
25021	GANAB	6590523	1.07	1.11E-04
5222	AP1G2	4260497	1.05	1.14E-04
24347	BC028528	7040014	1.04	1.15E-04
9415	IRAK1	6330546	1.06	1.18E-04
1841	FREQ	5550603	0.95	1.19E-04
8054	SLC14A2	4760612	1.05	1.26E-04
4668	TTC14	5570110	1.06	1.28E-04
2468	CACNA1F	4570593	1.07	1.29E-04
1247	SDCCAG1	4890349	1.07	1.35E-04
6359	SLCO6B1	3390255	0.93	1.37E-04
23531	1110031B1	1850059	1.05	1.37E-04
4359	BAMBI	4050382	0.96	1.42E-04
23486	CASP4	4290180	0.95	1.44E-04
12536	YIF1A	7040719	0.93	1.49E-04
24444	RPL13A	5340246	0.87	1.50E-04
5779	PHF14	5810347	1.06	1.56E-04
19680	AZI1	5490603	1.06	1.58E-04
1400	LOC100047	10411	1.06	1.66E-04
165	CLEC16A	5090739	1.07	1.67E-04
428	LASS4	2600437	0.93	1.67E-04
21241	PLEK2	3180035	0.95	1.67E-04
3525	TCSTV1	4120332	1.05	1.72E-04
7533	EG245297	2600128	1.07	1.75E-04
16427	TMEM132A	670072	1.15	1.78E-04
18738	CRMP1	5890739	1.13	1.79E-04
9974	GNAQ	1340452	1.05	1.80E-04
11140	OAT	4560309	1.14	1.86E-04
21416	PPM2C	2940487	0.90	1.87E-04
11284	2610039C1	2680202	0.91	1.87E-04
17032	ZFYVE26	5550050	1.06	1.90E-04
2964	POU4F3	3710176	0.95	1.91E-04
18290	KRTAP6-2	5220682	0.93	1.91E-04
1023	GTPBP6	3870438	0.87	1.93E-04
13371	PLEKHG1	6180039	0.96	1.95E-04
166	DPYSL2	3190474	1.09	1.95E-04
2617	SURF6	7160082	1.04	1.97E-04
21310	LCN8	2690731	0.96	2.03E-04
25	OLFR1366	6860093	0.94	2.05E-04
25242	MYL2	2120356	0.91	2.10E-04
23347	ZIK1	1770437	1.04	2.14E-04
5918	S100A8	1190546	0.62	2.17E-04
1575	BC031748	1030220	1.08	2.17E-04
13605	MAGEA3	70017	1.07	2.21E-04
5130	CPN2	6560482	0.95	2.26E-04
8078	TCF20	6400332	0.95	2.33E-04
20687	2810453I06	3370367	0.92	2.34E-04
24911	FGFBP1	4250437	0.94	2.36E-04
3790	SLC4A1	1230435	0.88	2.48E-04
67	YIPF6	5050706	1.06	2.49E-04
8236	SMOX	4050092	0.95	2.52E-04
6779	1700034H1	5870239	1.07	2.53E-04
10038	MBNL2	2710142	1.09	2.54E-04
22340	TAF1B	6400528	1.05	2.56E-04
222	EG317677	6760725	1.07	2.57E-04
225	GCC2	3170228	0.93	2.58E-04
20008	MARVELD2	3520528	0.92	2.59E-04
20481	CAMK2D	3190468	1.08	2.59E-04
10814	2410018C2	3780066	1.04	2.65E-04
22378	PSMB10	5870398	0.90	2.69E-04
11606	MORF4L2	2190239	0.91	2.69E-04
3608	ZFP202	7400634	1.06	2.75E-04
1671	CLEC2I	1090441	0.96	2.78E-04
13172	LAGE3	650059	0.91	2.88E-04
728	4833436C1	6040743	0.96	2.90E-04
14356	PIGZ	2120079	1.08	2.94E-04
46	C330005M1	7400678	0.96	2.94E-04
1216	TRUB2	7200181	0.95	3.01E-04
763	ANGPT1	510735	0.96	3.08E-04
14216	TRIM45	4640719	0.96	3.15E-04
5909	ACOX1	5090039	0.96	3.17E-04
5954	BNIP3	2190431	0.93	3.18E-04
1110	EAR10	7560228	0.96	3.23E-04
169	GART	4250681	1.06	3.25E-04

7140	LYPD4	3390564	1.09	4.06E-06
2366	SSSCA1	4560730	0.86	4.07E-06
18052	THYN1	2490008	0.89	4.15E-06
6921	PLAC8	5860243	0.94	4.17E-06
1761	TACSTD2	4590382	0.94	4.28E-06
19484	MRPL13	3710537	0.92	4.34E-06
125	RND1	990241	0.93	4.54E-06
9296	MFI2	6330112	0.92	4.66E-06
11301	ZMAT2	6980020	1.07	4.73E-06
12112	GM1604	7320750	1.07	4.79E-06
9126	4930503L1	2350022	1.06	4.87E-06
1483	RIPPLY1	3290692	1.04	4.94E-06
12029	TULP1	6020328	0.93	4.94E-06
10885	PALMD	3360408	0.95	5.11E-06
2565	NRCAM	4830040	1.11	5.14E-06
268	SLC12A9	4220112	0.93	5.31E-06
17207	PCYT2	6380678	1.11	5.38E-06
5077	ELMO1	130180	1.08	5.45E-06
12252	TRIM36	3130241	0.92	5.76E-06
7221	RAB43	4640193	0.93	6.05E-06
23455	QK	7400711	1.07	6.17E-06
24136	PI4K2B	5490411	1.06	6.57E-06
20315	PIF1	6940411	1.08	6.59E-06
1739	UNC13B	1110048	0.88	6.85E-06
333	LCE1H	70437	1.07	6.90E-06
4559	IL10RB	3800142	0.94	7.02E-06
323	EG331493	6480477	0.94	7.20E-06
10689	1700010D0	20368	1.08	7.41E-06
141	TRAFD1	3800068	0.91	7.47E-06
4595	DRD2	4850301	0.91	7.50E-06
23341	1700020O0	5910056	0.94	7.55E-06
138	CYP4F16	540075	0.90	7.83E-06
5421	4930529M0	4830167	1.09	7.86E-06
10248	ASB7	730630	1.05	8.03E-06
14325	MYH6	2230035	0.93	8.20E-06
19730	ZNRF2	4220674	1.07	8.21E-06
25455	SPHK1	4900767	0.95	8.32E-06
21209	CAST	3520164	1.07	8.40E-06
445	OLFR818	4920202	1.08	8.93E-06
18353	SPO11	6660292	1.11	9.02E-06
22003	FETUB	4540725	0.94	9.03E-06
19604	ZFP606	2680021	1.07	9.07E-06
161	SERPINA1A	360431	0.93	9.71E-06
2529	PHPT1	5360392	1.14	9.73E-06
5768	ASB18	3840707	1.06	9.88E-06
90	EG317677	2710465	0.94	1.01E-05
15999	3110040N1	620100	0.90	1.01E-05
9668	OLFR138	1110424	1.08	1.04E-05
14965	PRL8A6	2450164	0.93	1.04E-05
9678	6430537H0	6590341	0.95	1.11E-05
11238	NNT	2470646	0.95	1.14E-05
6072	PHKG2	2810228	0.91	1.17E-05
6487	C230096C1	5570187	0.94	1.21E-05
1425	PUS10	830538	1.04	1.21E-05
2883	UGT8A	2570228	1.19	1.23E-05
583	PAFAH1B1	4050221	1.07	1.23E-05
11742	OLFR820	1430162	0.92	1.27E-05
6689	OLFR371	4920131	1.06	1.27E-05
17389	DDX17	2760095	1.10	1.28E-05
360	SLC35C1	3390328	0.93	1.28E-05
10784	SH2D1B1	1780609	1.07	1.31E-05
9784	CCDC11	6280224	1.07	1.33E-05
5135	PCDHB15	5130605	0.96	1.35E-05
22098	CALR3	6840619	1.06	1.38E-05
6362	LOC100041	7550609	1.10	1.48E-05
4824	FKBP14	7560632	1.10	1.51E-05
16927	1810008A1	3870743	0.92	1.55E-05
10226	CASQ1	1050039	1.05	1.57E-05
5063	FIGLA	5860253	1.06	1.64E-05
9434	OLFR1378	1820564	1.07	1.66E-05
3912	CPA3	1410739	0.93	1.68E-05
235	1810026J2	6900632	0.90	1.73E-05
12367	CDC25C	3830328	0.93	1.75E-05
25314	LDLR	1070204	1.06	1.78E-05
12839	LOC100047	6060731	1.05	1.78E-05
7753	OLFR78	4760209	1.07	1.78E-05
16618	IGSF11	4640209	0.88	1.91E-05
2327	PCMTD1	2360196	1.07	1.93E-05
9239	TCF19	6330725	0.94	1.95E-05
4566	GLOXD1	6480609	1.06	1.96E-05
23234	PLD5	50719	1.05	1.99E-05
1310	GPM6B	6130168	1.04	2.01E-05
9878	FAHD1	4120692	0.89	2.02E-05
3456	LYZ1	2360719	0.94	2.13E-05
16802	ATRX	450400	0.94	2.15E-05
3785	TMUB1	6840609	0.92	2.26E-05
8813	FLOT2	2490739	0.92	2.36E-05
21321	ELOVL4	1740424	1.09	2.36E-05
979	CRKL	6840682	1.09	2.38E-05

606	MEX3C	1010446	1.04	0.001
8938	A	1780020	0.93	0.001
951	CLK2	2970646	1.06	0.001
11960	CSNK1G2	6480168	0.88	0.001
10133	UMODL1	6130154	0.94	0.001
3648	DUSP22	6060494	1.06	0.001
6551	ZFP709	5570754	1.06	0.001
9340	PABPC4L	5220703	1.04	0.001
2735	ZCCHC16	3120377	1.06	0.001
11553	F830116E1	6350215	1.06	0.001
9165	PBX1	1470315	0.95	0.001
4655	YWHAH	2190544	1.08	0.001
6479	AU040320	990202	1.06	0.001
1221	CREB3L4	6110133	0.95	0.001
5424	ACAD10	5570379	1.03	0.001
12529	BC024479	3850338	1.05	0.001
20880	HIST1H1B	1470050	0.95	0.001
9768	AKT3	7150521	0.94	0.001
7255	4931428L1	4390110	0.95	0.001
24826	NSL1	650048	1.04	0.001
1165	APH1B	6220612	1.06	0.001
2348	OTTMUSG0	780528	1.06	0.001
991	BC088983	3520138	1.05	0.001
342	EIF3G	4220433	0.84	0.001
45	CD207	5690056	1.07	0.001
14374	SFRS3	1340040	0.93	0.001
12921	B230118H0	830026	0.96	0.001
19004	HPGD	2450343	1.06	0.001
10500	LOC100046	7050446	0.90	0.001
114	COL25A1	1070196	0.92	0.001
9969	ARS2	1470356	1.13	0.001
15667	ROBO4	2630333	1.06	0.001
9647	CAMK2G	5260520	1.16	0.001
3555	GRAP	510162	1.06	0.001
22589	AA536749	4050370	1.05	0.001
11205	2310047M1	5690753	0.90	0.001
16054	DHRS7B	2100465	0.87	0.001
6273	OLFR1324	130639	0.96	0.001
17371	LYSMD4	2490427	0.96	0.001
6081	FBXO34	3120327	1.14	0.001
12131	MUC10	1030561	1.05	0.001
3602	SLC2A9	5220594	0.96	0.001
1812	CALU	1410091	1.04	0.001
4274	LRRC3B	2360563	1.07	0.001
2048	AFG3L1	7550400	0.95	0.001
12169	FTMT	3060730	1.03	0.001
20382	6720456B0	6620554	0.90	0.001
4691	KLHL32	6200079	1.08	0.001
24813	SLCO1B2	1410035	1.05	0.001
13574	KLHL26	4880133	1.05	0.001
1853	ARID3A	7400348	1.07	0.001
12942	OLFR121	6900475	0.93	0.001
24561	CORO2B	6480494	1.12	0.001
3509	POLM	3440301	1.05	0.001
22527	MRPL48	4220725	1.03	0.001
2890	GCM1	1450376	0.96	0.001
2189	IDS	1010368	1.09	0.001
6564	DDX56	160189	1.03	0.001
1778	PDLIM1	7560673	0.93	0.001
16333	SLCO1A5	20647	0.94	0.001
17196	ATXN2	4390593	1.11	0.001
192	LGALS12	4210176	1.06	0.001
15872	FANCD2	1940020	0.92	0.001
24048	SPG3A	60653	0.94	0.001
19205	MRPL49	3780435	0.93	0.001
1308	4932441K1	2760136	0.95	0.001
11599	AI316807	6100050	0.94	0.001
10900	ACIN1	4260209	1.05	0.001
14400	AGXT2	510554	1.04	0.001
709	PCDHB2	7550452	1.05	0.001
435	HTR4	1440672	1.07	0.001
6435	STX17	3130014	0.94	0.001
11929	AHCTF1	2690689	1.07	0.001
2420	NOTCH4	4250059	0.92	0.001
23694	TRIM2	3460064	1.17	0.001
5299	SLC36A1	2480400	1.04	0.001
994	RABL3	2970685	0.92	0.001
11774	EPB4.1L2	1770593	1.05	0.001
14406	UMPS	1410445	0.95	0.001
10414	OPLAH	730612	0.94	0.001
3496	EG245376	7570358	0.96	0.001
1900	OLFR707	5960685	0.96	0.001
6686	2310079F2	1190397	0.95	0.001
1057	SRXN1	610463	1.08	0.001
5020	ISCA1	5080156	1.14	0.001
22743	OLFR1037	3120114	0.95	0.001
5311	PNPO	2000593	0.89	0.001
10	ATP5SL	5270131	0.87	0.001
9014	KTN1	160014	0.95	0.001

6447	ELF3	4290075	1.05	5.20E-04
9606	RBPMS	2360717	1.05	5.25E-04
4402	SLC12A3	4260377	0.96	5.38E-04
8197	AGTR2	4760133	0.94	5.42E-04
3973	4921533L1	5390451	0.96	5.46E-04
5547	PDE1A	7210632	0.86	5.51E-04
21508	HDAC2	3780092	0.89	5.55E-04
7330	FAS	240301	1.04	5.70E-04
3040	DNAJA3	2000128	0.92	5.73E-04
1203	TNPO2	510129	1.08	5.79E-04
6147	P2RX6	110010	1.05	5.80E-04
4403	OLFR160	6480059	1.04	5.80E-04
16650	GM525	3130709	1.06	5.84E-04
13498	SOX6	4830239	1.04	5.88E-04
18534	ALG5	1400450	1.07	6.12E-04
23563	LGTN	3190427	1.06	6.15E-04
202	NLRP1C	4150066	1.06	6.34E-04
17917	OLFR1477	4220523	1.04	6.34E-04
1717	GNPTG	4540246	1.14	6.36E-04
5068	BANF2	70048	0.94	6.50E-04
14801	INPP4A	5360273	0.95	6.63E-04
3202	NIPSNAP3A	630162	0.95	6.66E-04
12039	P2RY10	6400070	0.96	6.80E-04
16192	CYP2A12	6960037	0.94	6.97E-04
5263	BC030476	1780037	1.16	6.98E-04
5950	OLFR1351	1070538	0.96	7.08E-04
839	TNRC6B	7160435	0.96	7.17E-04
11934	1110007L1	5270747	1.08	7.28E-04
8633	FCRLA	1260477	1.03	7.33E-04
18529	ZBTB24	3140608	1.09	7.38E-04
219	IL7R	1780079	0.96	7.39E-04
128	TRPC4	3060398	0.94	7.39E-04
24096	9530096D0	1050240	1.04	7.47E-04
3440	IGSF11	1660296	1.07	7.57E-04
6758	ITPK1	1030605	0.92	7.59E-04
7297	OS9	3520632	0.89	7.61E-04
22615	EDC4	5050241	1.07	7.68E-04
12905	OLFR1350	5570504	1.04	7.82E-04
20408	4932413O1	4200682	1.05	7.86E-04
1780	GTSF1L	2760470	0.96	7.91E-04
506	PCDHB14	50739	1.05	8.00E-04
2091	SMTNL2	6520056	0.95	8.03E-04
9754	MTERF	2470376	1.07	8.08E-04
7048	GYS3	5890372	1.06	8.12E-04
21516	MTSS1	6900133	0.95	8.25E-04
6014	OLFR1173	620220	1.06	8.29E-04
3891	SIT1	430746	0.96	8.35E-04
3161	ARL3	2450463	1.08	8.46E-04
446	NGP	730487	0.95	8.47E-04
6616	EIF2AK1	4220711	0.92	8.64E-04
664	SIPA1L3	4010341	0.96	8.67E-04
2947	WDR37	1170719	1.05	8.68E-04
23648	TNFSF8	1440152	0.95	8.77E-04
9912	ICOS	2450239	1.06	8.85E-04
992	GRIN2A	4230068	0.94	8.96E-04
13167	PRSS7	1940561	1.05	9.20E-04
4333	ABI3BP	4490730	1.12	9.23E-04
11611	TMCO6	5960332	1.05	9.24E-04
1937	OLFR45	3140743	0.96	9.27E-04
8121	HHEX	450736	0.95	9.46E-04
3724	PKHD1L1	630400	0.95	9.50E-04
24904	FLT1	3830114	0.89	9.57E-04
12534	SLC44A2	3840600	0.97	9.60E-04
1184	CYPT10	3360463	1.04	9.64E-04
8446	CECR5	1260494	1.07	9.92E-04
1279	DPPA5	7150711	0.95	0.001
5056	SH3D19	1780273	1.04	0.001
6361	PTCRA	5360672	0.94	0.001
12735	DDB2	4900474	1.08	0.001
5629	SSTR3	460333	0.95	0.001
16450	BC002059	1850048	1.04	0.001
4135	OLFR1045	6270681	1.06	0.001
21191	RGS3	6840192	1.04	0.001
16745	NR2E3	3450739	0.97	0.001
15513	JAM2	1470215	0.96	0.001
10902	NRIP2	3130451	1.07	0.001
3353	SAR1B	6960215	1.13	0.001
704	PTPRF	60400	0.95	0.001
5482	1110020C0	3130703	0.96	0.001
4437	BC049730	4850253	0.95	0.001
16968	OLFR120	4280435	1.04	0.001
21636	NDN	4490367	0.79	0.001
946	OLFR1344	770364	0.96	0.001
2440	LTBP1	6290463	0.94	0.001
582	EGFR	1770292	0.96	0.001
25269	ADO	1050521	0.97	0.001
8859	ERCC8	7650224	1.07	0.001
217	WIPI2	2190612	0.91	0.001
22740	NDOR1	430102	0.95	0.001

4375	MAPK15	510538	0.94	9.86E-06
6278	CD300A	2490097	1.05	1.00E-05
15396	INTS4	870653	0.93	1.02E-05
17023	APOA2	6350537	1.09	1.04E-05
10786	COPS7A	3890093	1.09	1.05E-05
18826	RGS18	460138	0.94	1.05E-05
19349	PPP5C	6620750	1.10	1.07E-05
4014	BC005764	7510553	1.09	1.08E-05
9923	GSDMA3	150170	1.07	1.10E-05
10460	ITIH3	1570301	0.91	1.11E-05
13850	IFNA14	3460546	1.04	1.12E-05
7804	CREB1	620707	0.91	1.13E-05
1145	OTTMUSG0	2350035	0.94	1.14E-05
1745	PER2	5860553	1.05	1.17E-05
13130	CEACAM16	450113	0.93	1.17E-05
3518	GLRA2	20487	0.84	1.20E-05
1313	SYNCRIP	6200181	1.08	1.20E-05
13050	1110057K0	290296	0.90	1.20E-05
13899	FOXK1	1510273	1.09	1.21E-05
3906	DOK1	2120427	1.05	1.22E-05
214	NDUFA5	6560152	1.15	1.25E-05
3955	TLL1	2680239	1.06	1.34E-05
12659	SLC26A9	630280	0.95	1.35E-05
3717	CXCL16	630274	0.95	1.36E-05
8748	CTRL	4490324	0.94	1.37E-05
17420	CDH4	160132	0.93	1.38E-05
4234	LOC100046	5670114	1.06	1.45E-05
23099	VEZT	3060438	0.90	1.46E-05
11389	OLFR398	4730343	0.96	1.46E-05
16076	PHF11	1340193	1.05	1.54E-05
22134	EYA2	5390095	1.08	1.56E-05
11555	EFHA1	2850338	1.13	1.57E-05
22259	NINJ1	4210553	1.09	1.60E-05
2729	CYP2C29	4730403	1.08	1.60E-05
1161	MYO1A	2360753	0.92	1.64E-05
514	RGS3	5340592	0.94	1.64E-05
23834	LOC237877	7150431	0.94	1.70E-05
21495	ITGB3	1740451	0.93	1.73E-05
3254	BTN2A2	2940327	1.06	1.73E-05
480	LHB	1240685	0.93	1.74E-05
2778	OTTMUSG0	2900639	1.07	1.79E-05
17666	VSIG1	2060491	0.93	1.80E-05
11420	6330439K1	240156	1.09	1.81E-05
22591	L3MBTL3	7330685	1.07	1.82E-05
20257	CAPN12	3780504	1.05	1.82E-05
14557	PPARGC1B	6860138	0.89	1.82E-05
23442	GCC1	1240563	0.94	1.88E-05
1065	GM784	4220167	1.09	1.89E-05
12480	ANXA10	6650220	1.03	1.90E-05
6878	CAV2	4480576	1.08	1.91E-05
13169	DEADC1	2810139	1.12	1.94E-05
2765	MED22	6510521	1.12	1.95E-05
12077	MRPL47	2230670	0.89	2.02E-05
9317	EPHA1	3440376	1.06	2.05E-05
1134	ATOH8	3440445	0.92	2.08E-05
22101	COPG	6560056	1.17	2.08E-05
6117	CYC1	5560451	1.09	2.09E-05
7007	ELP4	5050398	0.94	2.11E-05
18318	MON1A	1050598	1.09	2.23E-05
11928	2010305A1	430240	1.10	2.25E-05
11148	SBP	2140220	1.07	2.27E-05
6492	CPB1	5080605	1.04	2.27E-05
3038	CYBRD1	3890148	1.06	2.27E-05
7180	SOX5	1340128	1.07	2.30E-05
2716	VMN2R89	3310592	0.94	2.34E-05
15661	SFXN3	5820097	1.07	2.35E-05
11612	ZFML	240220	0.92	2.36E-05
7997	ADORA2B	7000289	1.08	2.42E-05
10992	PCSK1	6550243	0.95	2.44E-05
6314	KLHL1	6650594	1.07	2.51E-05
9419	VMN2R84	4040327	0.93	2.58E-05
13527	RNPEP	1740711	1.10	2.64E-05
1618	RBM41	6290333	1.07	2.69E-05
24280	TRPV1	1510170	0.95	2.73E-05
3249	SSR4	510349	1.17	2.74E-05
4507	AB112350	290703	1.07	2.74E-05
17179	FLT3L	5340053	1.09	2.74E-05
3693	GLS2	3610382	1.07	2.77E-05
21174	OTUD7B	6220152	0.93	2.78E-05
15602	MXRA8	1230672	0.92	2.80E-05
25665	ARHGAP17	6520162	1.06	2.85E-05
15465	USP15	5860377	0.94	2.91E-05
2690	NUP133	6370288	0.91	2.99E-05
2870	OTTMUSG0	4640639	0.94	3.03E-05
16135	MZF1	3120053	1.06	3.08E-05
4754	OLFR1261	6100066	1.05	3.09E-05
2264	BC094916	1690292	0.94	3.09E-05
3359	ZP2	1450129	1.07	3.12E-05
2383	CCDC28A	6860564	0.91	3.13E-05

8	CCDC90B	6100215	1.10	7.33E-04
605	ERCC6	4200259	0.94	7.35E-04
2238	ACOXL	6400333	0.96	7.45E-04
12298	SIRT5	5890619	0.92	7.46E-04
18550	RAET1A	4220181	1.05	7.62E-04
22251	HN1	1470528	1.06	7.63E-04
7392	ILF3	460301	0.90	7.68E-04
410	MPO	6280168	0.95	7.80E-04
15172	UBE2J2	2690338	0.96	7.91E-04
2517	ACPL2	650086	0.95	7.93E-04
11382	DSG1C	2600279	0.95	7.97E-04
17499	CTSS	4200646	0.94	8.05E-04
994	RABL3	2970685	0.94	8.07E-04
4897	BC017647	20349	0.89	8.08E-04
4196	USH1C	2600431	1.06	8.23E-04
8158	DAPP1	4250537	0.96	8.30E-04
9193	AB041803	3370189	1.05	8.43E-04
4360	PURG	1660086	0.95	8.45E-04
12243	MOV10	1090259	1.05	8.47E-04
8153	REG4	6770598	1.05	8.52E-04
2166	ST6GALNA	6560692	0.94	8.56E-04
3908	ZCCHC17	6370079	1.10	8.59E-04
1672	CD109	3460575	0.93	8.64E-04
14753	KLK8	6200446	1.07	8.64E-04
4708	MALL	5220041	1.05	8.66E-04
9247	CXXC4	6560398	0.92	8.68E-04
4123	POF1B	6620368	0.93	8.83E-04
3503	CCDC109B	6620609	1.04	9.15E-04
964	KLRB1C	6100184	1.06	9.24E-04
498	2310047O1	4830725	1.07	9.26E-04
9400	ATP5C1	1030338	1.17	9.28E-04
769	D530033C1	3840326	1.08	9.68E-04
17690	2610021K2	4150131	0.96	9.74E-04
453	ARMC6	1510681	0.89	9.83E-04
4356	TCEAL8	1070047	1.10	9.86E-04
22687	MTUS1	3060021	0.95	9.97E-04
15705	MDH1	240593	1.10	0.001
7572	HERC3	2480243	0.91	0.001
7495	V1RA4	6420270	1.04	0.001
16315	TAAR6	7320048	0.96	0.001
2853	MRPL22	1940358	1.12	0.001
1118	TPRA40	6130193	1.07	0.001
12099	CUX2	1050132	1.06	0.001
18517	GABRB2	4220327	0.96	0.001
17145	AYTL2	3930195	1.06	0.001
14252	TAAR9	5050441	1.04	0.001
6395	TNFRSF25	6370520	0.94	0.001
3071	IRX3	60703	0.96	0.001
7358	3110032G1	7100682	0.94	0.001
17	OLFR92	2710201	1.05	0.001
21063	ZDHHC22	6380494	0.94	0.001
18794	EG269902	1030360	1.07	0.001
8666	2610029G2	1580097	0.94	0.001
20222	RPS23	7380373	0.95	0.001
11172	ANKRD23	2850064	1.06	0.001
14477	DISC1	650154	0.96	0.001
20925	RAPGEF1	1470609	0.95	0.001
22341	TBPL1	460093	0.96	0.001
2207	SMC1A	6330102	0.97	0.001
11366	4933429E1	5700053	0.96	0.001
3607	HTR2C	5870280	0.96	0.001
17487	2210407C1	5340274	0.96	0.001
3600	FEZF2	770615	0.91	0.001
284	OLFR1497	4290626	1.06	0.001
8705	CRH	670019	1.04	0.001
615	SLC39A4	2470086	1.04	0.001
13147	ZER1	4050138	0.90	0.001
958	EG432723	6450121	0.96	0.001
1163	DYNC1LI1	6560441	0.92	0.001
9052	SDHB	3180075	1.12	0.001
7895	ATP5O	5820563	1.09	0.001
11859	SEPT5	1850041	1.12	0.001
1380	4930544G1	4760369	0.94	0.001
15990	E130012A1	540398	1.12	0.001
19286	GUCY2C	4070719	1.04	0.001
25205	LEMD2	2470528	0.94	0.001
1452	SLC1A6	6180332	0.95	0.001

22449	EPN2	2260280	1.10	3.26E-04
11769	SNURF	3130246	1.12	3.27E-04
2861	PPIE	3290278	0.93	3.32E-04
24105	DCTN3	540338	0.89	3.32E-04
11315	ETFDH	2570433	1.09	3.32E-04
25310	LRRN3	4590619	1.10	3.34E-04
7550	OLFR1024	4050735	1.06	3.42E-04
2231	AMMECR1L	6660608	1.10	3.42E-04
2098	GRHL1	150072	1.04	3.52E-04
269	BC043934	6590424	0.95	3.58E-04
4695	STK19	3400408	0.85	3.59E-04
12315	PRKX	7560022	0.96	3.60E-04
22016	5830472M0	4480747	1.08	3.60E-04
757	BC026439	3360008	1.05	3.63E-04
4518	SLAMF6	5720349	1.04	3.87E-04
14091	4931417G1	3450706	1.05	3.93E-04
665	OLFR1502	6940377	1.06	4.00E-04
207	ZFP817	2900504	1.05	4.11E-04
20254	GIPC1	5720632	0.92	4.12E-04
2803	ZFP523	6510427	0.91	4.17E-04
1668	9030625A0	1570703	1.04	4.27E-04
7585	SPAG4L	130646	1.05	4.34E-04
18183	RUVBL1	610609	0.90	4.36E-04
5420	CACYBP	150367	1.04	4.51E-04
2510	ORF63	1400747	1.07	4.53E-04
6279	CTSQ	270292	1.05	4.57E-04
6830	TNRC6A	540239	1.09	4.64E-04
16613	BOLA1	3360274	0.92	4.71E-04
6841	NECAB3	870521	1.10	4.72E-04
2259	ZFP786	2490411	0.96	4.75E-04
24608	OLFR77	6520364	0.95	4.77E-04
16471	AI427515	450452	1.17	4.80E-04
19191	AVPI1	3520093	0.92	4.80E-04
13553	RPGRIP1	2030019	0.95	4.83E-04
11104	5730449L1	6420672	0.89	4.90E-04
15099	FRY	1010240	1.05	5.02E-04
21125	RPS15	5570129	0.88	5.09E-04
12936	NDUFA8	4220519	0.93	5.19E-04
2897	OLFR1518	6510168	1.05	5.19E-04
3930	EG328264	2650075	1.04	5.22E-04
3309	SPNB2	650630	1.15	5.29E-04
11213	RPUSD1	1260546	0.94	5.32E-04
22858	PSMG3	3130326	0.91	5.41E-04
12661	OLFR1036	5910326	1.05	5.42E-04
20630	EIF2S3Y	6960730	1.08	5.42E-04
18665	GSTA3	5550075	1.05	5.42E-04
25200	ASNS	1260164	1.18	5.44E-04
6896	PLEC1	6760347	1.09	5.45E-04
3092	GATA3	5900070	0.96	5.48E-04
8411	LOC100044	4890524	1.10	5.49E-04
16131	PSME2	1240072	0.91	5.49E-04
1722	OLFR234	1850309	0.96	5.51E-04
18689	RAB36	5490307	1.07	5.53E-04
3698	SNRPA	2230647	1.09	5.54E-04
17991	RPS11	1410161	0.88	5.61E-04
16897	C8G	3780601	0.94	5.64E-04
13461	MPL	3460392	0.95	5.66E-04
272	TMEM138	3370162	0.96	5.73E-04
8856	CHRNA3	1240164	1.04	5.75E-04
19222	OLFR1157	7150717	1.04	5.84E-04
25240	TMPO	10181	1.06	5.92E-04
25623	PNKD	3460731	0.95	5.96E-04
11720	SEC22A	3390072	1.04	6.02E-04
2699	EPSTI1	2760274	0.95	6.04E-04
4889	1110018G0	3420075	1.07	6.09E-04
2658	COL12A1	3870360	1.09	6.25E-04
3631	MPP3	3890154	0.92	6.28E-04
34	D030070L0	1510433	1.05	6.45E-04
13319	VARS	1580044	0.94	6.60E-04
1237	V1RC19	730719	0.96	6.63E-04
20667	LOC100048	3310091	1.09	6.66E-04
18388	KCNMA1	4560554	1.11	6.73E-04
20394	GSTO1	1010435	1.07	6.74E-04
12262	ARRDC3	1980411	1.10	6.78E-04
8305	OLFR822	4390059	0.96	6.92E-04
529	POP1	940670	1.04	6.94E-04
14087	4831426I1	5560131	0.95	7.05E-04
11596	ZFAND2B	1770242	0.94	7.08E-04
456	CCT8	4250386	1.17	7.21E-04
5210	4930555I2	380441	0.94	7.27E-04
2154	SCGB3A1	4810017	0.96	7.29E-04
24659	SV2A	160484	1.24	7.36E-04
205	OTOR	6840328	0.96	7.37E-04
6926	4933404M0	780653	0.94	7.51E-04
201	HARBI1	5130095	0.93	7.57E-04
724	GLIS2	4810739	1.05	7.64E-04
4183	TTLL8	1240026	1.06	7.84E-04
11530	0710008K0	510445	0.90	7.97E-04
3594	MIA1	2480139	0.94	8.04E-04

17399	FUBP1	1980400	0.94	2.43E-05
12578	RAPGEFL1	1010524	0.84	2.44E-05
405	ECHDC1	5900747	1.10	2.48E-05
483	KDELC1	2360131	0.93	2.49E-05
3043	NET1	3840553	1.09	2.49E-05
23347	ZIK1	1770437	0.95	2.56E-05
17	OLFR92	2710201	1.06	2.60E-05
1714	1810044A2	5810288	1.06	2.64E-05
2205	PDK2	5890139	1.08	2.64E-05
92	HMGCLL1	620441	0.94	2.64E-05
22621	OLFR972	2230445	0.93	2.67E-05
10368	SPAG5	2190064	1.06	2.70E-05
15	CD177	670452	0.93	2.71E-05
19402	LSM2	6480520	0.92	2.75E-05
1293	A530099J1	6590592	1.08	2.75E-05
623	1700008I0	5490092	0.94	2.76E-05
52	LOC100046	1770379	0.92	2.77E-05
7417	NKX3-1	2810731	1.05	2.87E-05
1424	RALBP1	2650370	0.87	2.90E-05
12516	BANF1	2750092	0.90	2.98E-05
18021	OLFR1156	10487	1.06	3.01E-05
14138	RIC3	4050554	0.96	3.11E-05
16198	FBXO3	5670543	1.07	3.11E-05
22792	WARS	3450735	1.05	3.13E-05
1529	SLC6A5	780025	0.95	3.21E-05
21078	RRH	4560338	1.05	3.30E-05
84	MGC18837	5810541	0.91	3.32E-05
504	DCUN1D3	1070438	1.07	3.34E-05
3848	IQGAP2	6130014	1.08	3.38E-05
5858	EXTL3	610528	1.13	3.38E-05
21793	TBX22	7100064	1.06	3.39E-05
23011	TOP3A	290292	1.06	3.42E-05
588	EIF4G2	6520484	1.11	3.43E-05
351	HS6ST1	6350402	0.89	3.49E-05
14311	1700008F2	5870138	0.92	3.55E-05
3916	RBM12	4290019	0.93	3.56E-05
23705	XPO1	150424	1.06	3.58E-05
9887	IRF6	4390040	0.94	3.73E-05
18978	OLFR1026	940593	0.94	3.74E-05
16081	GPX3	7320053	0.94	3.76E-05
16886	1110002H1	4570707	0.95	3.83E-05
10549	OLFR1309	4260576	1.06	3.86E-05
12512	SRRM2	3420273	1.16	3.87E-05
20982	GPT1	520634	1.17	3.89E-05
6590	MOGAT2	4670017	1.05	3.96E-05
17598	SETD4	1010079	0.93	4.01E-05
7904	2410014A0	1690204	0.93	4.05E-05
298	OSMR	5910220	1.08	4.08E-05
2608	CDH26	5700753	1.05	4.08E-05
15176	NEIL2	2760524	0.93	4.13E-05
21413	2310001A2	1430170	0.88	4.21E-05
7371	BC011426	6940446	0.96	4.26E-05
947	ARHGAP24	6550470	1.07	4.26E-05
863	WDR75	4280142	1.08	4.27E-05
7483	SNTB1	4830577	0.97	4.36E-05
6768	CD320	5420008	0.91	4.41E-05
22563	LRIT1	3420070	0.96	4.42E-05
16749	4930403L0	380626	0.95	4.49E-05
9120	THA1	6400754	1.06	4.57E-05
7023	OLFR532	10189	0.95	4.60E-05
3840	9930013L2	60474	0.96	4.61E-05
8749	4933436I0	70433	0.94	4.62E-05
11937	D13WSU17	6560121	1.07	4.63E-05
12996	PLEC1	4280228	0.95	4.66E-05
13476	TTC23	1450164	0.95	4.70E-05
6027	CLN6	380220	0.94	4.72E-05
10463	GPR146	7650152	1.11	4.72E-05
1944	CDKL4	1110110	0.92	4.79E-05
24656	MEGF9	4250609	1.11	4.81E-05
8781	IL23R	130164	0.95	4.84E-05
346	BCL2L1	7510608	1.06	4.85E-05
14527	TNFRSF10B	2370017	0.95	4.89E-05
24969	TRPT1	5420524	1.07	4.93E-05
8054	SLC14A2	4760612	1.09	5.03E-05
11408	RS1	1450402	1.06	5.07E-05
21890	TMEM168	2650424	0.95	5.16E-05
1222	TRIM21	3840634	0.94	5.24E-05
21516	MTSS1	6900133	1.04	5.27E-05
2361	PPARG	2320402	0.95	5.27E-05
1252	IKBKG	2470670	1.06	5.32E-05
6789	GM249	5910470	1.05	5.35E-05
5271	SFRS10	5290019	0.95	5.41E-05
3257	UBAP1	110162	0.89	5.54E-05
14979	SPC25	2000270	0.94	5.69E-05
23796	2810048G1	4040133	0.96	5.76E-05
7660	CACNB1	4610546	1.07	5.82E-05
4382	LOC433722	2810497	1.04	5.86E-05
822	KCNJ3	1820544	1.08	5.86E-05
7235	2610208M1	4490091	0.95	5.88E-05

2253	HES6	7000634	0.90	0.001
3718	EXT2	5890435	0.93	0.001
2466	BC021785	2100296	0.95	0.001
13104	INPP4A	2690563	0.97	0.001
14276	ARL6IP4	20037	0.91	0.001
1495	ZBTB46	6960639	0.95	0.001
11609	CCDC142	290373	1.03	0.001
17091	TRIM3	1070551	1.08	0.001
10951	TAGAP	4920639	0.96	0.001
3911	SCNM1	160747	0.89	0.001
9191	STARD6	7320575	0.93	0.001
10697	SLC26A9	4560450	1.06	0.001
3510	CTSW	1570133	0.96	0.001
16781	POLE3	6650672	0.91	0.001
2471	H1FNT	1660646	0.95	0.001

25599	TBX20	2370358	0.94	0.001
447	OPN1SW	4890142	1.06	0.001
3779	OLFR164	290170	0.95	0.001
8150	PCGF6	20025	1.09	0.001
4796	ASB17	5340112	0.96	0.001
3839	CCDC102A	990372	1.06	0.001
21554	MRPS25	4490450	1.06	0.001
17708	STMN1	3120392	0.93	0.001
20080	CCBL1	2850746	1.10	0.001
1807	NTAN1	50400	1.10	0.001
11345	BC016201	7560551	0.96	0.001
19579	STXBP1	1300446	0.93	0.001
16029	LOC100045	5490279	0.94	0.001
1166	RBBP7	670711	0.89	0.001
2835	SLC24A4	5690546	0.96	0.001
221	EPHA5	2370692	1.05	0.001
7155	ARF3	5570280	0.91	0.001
9809	ATP6V0E2	1010703	0.85	0.001
4045	SFRS11	5560537	1.09	0.001
3852	V1RH20	3390139	1.05	0.001
9162	CTNNA1	70753	0.93	0.001
11349	ZFP78	7550092	0.94	0.001
5841	TBX6	1230458	1.07	0.001

10201	2310042D1	5130593	0.95	3.20E-05
46	C330005M1	7400678	0.95	3.20E-05
1439	KLHDC1	2490475	1.09	3.26E-05
17842	PRPF40B	830563	0.94	3.29E-05
24762	GDF9	6860349	1.05	3.31E-05
772	LOC100044	4880097	0.94	3.35E-05
3377	AURKA	380520	0.94	3.40E-05
13096	YLPM1	6250754	0.94	3.45E-05
15993	BCAP31	3610424	1.15	3.57E-05
14455	PLAUR	5720497	0.92	3.61E-05
20791	OLFR476	1940193	1.08	3.66E-05
19014	FOXI1	5550279	0.95	3.75E-05
19687	MFN1	6860523	0.95	3.76E-05
3538	IGHMBP2	3170180	0.95	3.77E-05
12599	BLM	5960189	0.93	3.77E-05
3630	HOXD4	4640484	0.94	3.81E-05
19573	LOC100047	2600324	1.08	3.83E-05
2003	DLGAP4	1580711	1.07	3.85E-05
8049	KATNAL1	6350437	0.93	3.91E-05
1709	LOC100048	10239	0.94	4.03E-05
8399	G6PDX	240653	1.07	4.08E-05
15065	CCL4	7150500	1.09	4.28E-05
8684	SLFN1	3520746	1.07	4.29E-05
9946	AKR1A4	940474	0.90	4.30E-05
6910	CUL4B	3310767	1.09	4.30E-05
17252	OLFR593	1820253	1.05	4.35E-05
10476	EG240327	3400451	1.04	4.38E-05
14348	CIDEB	10575	1.09	4.46E-05
7175	LCAT	4810072	1.11	4.52E-05
18420	PI4KB	1090674	0.93	4.54E-05
18247	HAP1	5570601	0.95	4.58E-05
15433	TFPT	5420259	1.14	4.63E-05
9112	PPFIBP1	160577	0.95	4.65E-05
17017	SIGLECH	70463	1.10	4.67E-05
18694	OAS1F	4230300	1.06	4.68E-05
9131	XPO4	6420541	0.94	4.74E-05
4060	SLC25A20	1090037	1.09	4.80E-05
11856	9130422G0	2450259	0.92	4.81E-05
38	LOC100046	3390047	0.95	4.82E-05
16604	HMX2	3400278	0.91	4.87E-05
5188	TMPRSS11A	5860427	0.94	4.89E-05
24106	BBS9	6270088	0.93	5.03E-05
417	LOC100048	2060156	1.06	5.23E-05
17000	ACPP	2570280	1.06	5.33E-05
5800	OLFR460	3400687	0.95	5.41E-05
4282	C3AR1	4670743	1.05	5.46E-05
563	CRY1	3290364	0.95	5.47E-05
4431	LOC100044	4890370	0.95	5.55E-05
4374	SLK	6560477	1.07	5.61E-05
3575	PML	4120142	1.07	5.68E-05
13843	PPP2R5C	6900400	0.95	5.81E-05
925	LSM14B	7610086	0.94	5.82E-05
15643	4933425M1	4230739	1.06	5.88E-05
437	GLI3	6040707	1.04	5.93E-05
21795	LOC100044	2570661	1.05	5.99E-05
19311	ERLIN2	5390520	0.94	6.07E-05
14837	4933439F1	2100431	1.06	6.14E-05
3352	KCTD9	4880019	1.08	6.19E-05
4485	ZBTB7C	2260398	1.06	6.21E-05
12995	ARHGAP19	1340047	0.96	6.26E-05
20414	2310005E1	2120243	1.10	6.41E-05
23914	ZGPAT	160500	1.05	6.41E-05
21456	ABCA3	4040497	0.94	6.42E-05
15245	AKR1B3	1340398	1.07	6.44E-05
11887	PSME3	2940097	0.87	6.56E-05
13799	PIGL	6250288	0.94	6.57E-05
19463	LOC329575	2760519	0.95	6.62E-05
3062	UBE2D3	1190504	1.08	6.72E-05
2862	BRSK1	1850433	1.06	6.73E-05
6944	COPA	5560433	0.85	6.75E-05
20858	SMOK3B	5910706	1.05	6.78E-05
19680	AZI1	5490603	0.94	7.06E-05
12400	EFCAB2	2370546	0.93	7.08E-05
1908	TCP11L1	3870646	0.94	7.11E-05
2490	DHX15	4670768	1.10	7.16E-05
1086	LOC669251	3140709	0.96	7.16E-05
2548	PTPN22	5900593	0.95	7.17E-05
8967	P2RX5	6330451	1.05	7.20E-05
20501	CEP78	730739	1.09	7.22E-05
15698	1700001O2	4610500	0.95	7.23E-05
19177	ORF9	1070192	0.94	7.28E-05
22191	SLC25A21	2470035	1.05	7.37E-05
15397	ATF2	3400360	0.95	7.39E-05
9556	A730017C2	830358	0.87	7.44E-05
18453	FZR1	160446	1.06	7.49E-05
16334	TMEM49	6480458	1.07	7.52E-05
16865	ITLNB	4120431	0.95	7.69E-05
66	CAPRIN1	1510201	1.08	7.70E-05
5310	RAD23A	1500129	0.89	7.74E-05

6618	MAMDC4	4610154	1.04	8.08E-04
521	OBFC2A	610273	1.06	8.09E-04
11626	MAGEB3	770561	0.93	8.17E-04
17549	GSS	5390102	1.05	8.23E-04
14526	PLA2G4A	1450653	0.96	8.23E-04
15127	MTAP1B	5810332	1.25	8.27E-04
73	A930025D0	2600253	0.96	8.29E-04
23030	RPL19	4900703	0.89	8.33E-04
734	SNAP91	5670093	1.10	8.57E-04
9846	S100A9	1980603	0.87	8.60E-04
1570	CAMP	450670	0.92	8.62E-04
4600	CD109	360445	0.96	8.71E-04
9785	4921505C1	2450717	1.11	8.72E-04
9939	TCERG1	1990575	1.04	8.90E-04
9357	DPY30	4230521	0.91	8.91E-04
10303	SLC6A17	6770255	1.12	8.96E-04
7394	FNDC7	6330152	1.06	9.05E-04
23254	SCAMP1	160240	1.13	9.07E-04
7684	GM815	4880521	0.96	9.12E-04
24719	LOC100044	1990482	0.91	9.14E-04
345	TRABD	2690240	1.07	9.20E-04
22842	OLFR918	4290184	0.94	9.21E-04
1341	NUDT13	4070026	1.07	9.22E-04
18742	1810009O1	4150639	0.95	9.26E-04
19517	PPAP2B	1660687	1.10	9.34E-04
15326	SPATA16	3370497	0.96	9.39E-04
19015	VAT1	2900402	1.07	9.53E-04
7029	C2CD3	2630669	0.96	9.58E-04
7024	POLE	4060441	0.97	9.64E-04
5926	HCST	1510497	0.95	9.71E-04
7981	CCDC109A	2350538	1.09	9.73E-04
7863	PVR	650458	1.05	9.81E-04
3935	MYH4	4220474	1.04	9.85E-04
689	IFT140	4180411	1.05	9.95E-04
24199	AMN	2510743	0.84	9.96E-04
16929	PDLIM7	6550609	1.08	0.001
72	FECH	3420291	1.05	0.001
4578	IL17D	5130682	0.94	0.001
1013	EFCAB4B	2070561	0.94	0.001
22745	HSD11B1	4050369	0.88	0.001
21435	MRM1	1300392	0.94	0.001
15425	4933421E1	5720767	0.89	0.001
15162	PSMB9	2450064	0.94	0.001
1760	CRLS1	6960332	0.90	0.001
14548	SPRR2A	60326	0.95	0.001
9294	LRSAM1	1190392	0.95	0.001
15442	CD300LF	3170487	1.05	0.001
1788	LEP	6280059	0.96	0.001
14667	GSTA4	360348	0.93	0.001
10188	LOC100048	6660152	1.05	0.001
11912	PI4KA	1260241	1.17	0.001
13919	DUSP2	4860379	1.05	0.001
23941	MRPS5	4180195	0.96	0.001
9370	TIMM9	610075	0.94	0.001
5034	PPL	5220397	1.05	0.001
9581	KRT15	4570278	1.09	0.001
17725	BAPX1	6480097	0.95	0.001
3812	MUP2	7050706	0.96	0.001
2207	SMC1A	6330102	1.03	0.001
7209	KSR2	1770307	1.04	0.001
10562	ZFAND2B	2070170	0.86	0.001
21157	PROSAPIP1	2030279	0.91	0.001
684	D6ERTD474	1240477	1.04	0.001
1711	TTL	6380601	0.92	0.001
1471	V1RH13	1070736	1.07	0.001
14333	EG654432	2760735	1.05	0.001
15687	CRHR1	6900114	0.94	0.001
19934	XKR7	20192	0.94	0.001
13389	ZFP512	3850039	1.13	0.001
13908	BC043301	3780270	1.05	0.001
17897	HERC4	4220338	0.93	0.001
1812	CALU	1410091	1.05	0.001
12644	D630044L2	1050386	1.05	0.001
7185	ZXDC	1940692	1.05	0.001
1158	D6WSU163	4010129	1.08	0.001
5838	MPV17	4260053	0.90	0.001
8119	A930021C2	1990168	1.05	0.001
11582	ADD1	3850386	1.07	0.001
6560	SFMBT1	1110170	1.05	0.001
3913	IGSF1	360209	0.95	0.001
7520	TIMM44	6220450	0.91	0.001
16588	PI4KB	2070408	0.92	0.001
2898	OLFR1437	6510709	1.05	0.001
10791	TRIM32	130433	1.22	0.001
3042	FRG1	6940348	0.96	0.001
21118	B930006L0	3460368	1.16	0.001
22489	RBM3	2490307	1.04	0.001
10195	G0S2	20327	0.93	0.001
14029	LCN4	5870014	0.97	0.001

8565	2610507B1	1190095	1.11	5.94E-05
3874	MESDC2	6040673	1.05	6.00E-05
11072	2010106G0	270450	0.93	6.03E-05
3742	HERPUD2	6620132	1.06	6.05E-05
788	BCL2L2	1980553	0.90	6.16E-05
104	OLFR1517	510100	0.94	6.18E-05
19378	SLC10A7	3450653	0.94	6.28E-05
21137	CDC37	2360112	0.93	6.38E-05
21362	INADL	4230673	0.93	6.56E-05
6882	TIMP1	4640215	0.89	6.74E-05
18541	TAF9B	3780040	0.94	6.90E-05
6536	BTG2	2060246	0.95	7.08E-05
19537	ZFP71-RS1	7150600	1.05	7.19E-05
3055	2010110P0	6770743	1.11	7.23E-05
24782	LOC545732	7650630	1.05	7.28E-05
7424	TBX6	6130121	1.06	7.32E-05
21949	TSNAXIP1	5820079	0.95	7.35E-05
2870	OTTMUSG0	4640639	0.94	7.47E-05
11767	GALNTL2	3840156	1.06	7.51E-05
382	LAX1	2810722	0.95	7.62E-05
24499	PWP2	2260435	0.95	7.68E-05
10159	BC049635	7510703	0.95	7.79E-05
5051	GM949	1500020	1.06	7.84E-05
25374	EFCAB4A	6020136	0.96	7.86E-05
20441	RBM24	3930164	0.93	8.01E-05
1910	TMEM16J	2600070	1.05	8.06E-05
18829	SYTL2	4590537	0.95	8.15E-05
14612	SAA4	7200392	0.95	8.18E-05
11748	ABCA13	5900068	0.95	8.19E-05
10762	POR	2070563	1.08	8.21E-05
3787	GLE1	1340221	1.07	8.22E-05
5952	TMEM77	4920494	1.05	8.25E-05
16242	ADD3	7650246	0.90	8.31E-05
3884	OLFR1155	5080592	1.06	8.53E-05
2054	CLK4	7100519	1.06	8.97E-05
20074	MYST1	6370674	0.93	9.01E-05
14709	1110019N1	780041	0.93	9.05E-05
2073	SPATA22	5490377	0.94	9.16E-05
9874	TNFRSF17	2940482	0.92	9.18E-05
5839	ANKMY2	6580372	1.05	9.33E-05
12611	EDA2R	4730431	0.95	9.73E-05
13835	SUSD3	1690255	1.07	9.84E-05
2137	SPNA1	2810544	0.93	9.97E-05
6786	LOC236749	5360563	0.94	9.97E-05
6272	HEXB	4670056	0.96	1.01E-04
4774	AP2B1	5550400	1.07	1.03E-04
3425	PRG3	5490717	0.96	1.06E-04
2106	APBB1IP	3460747	1.06	1.08E-04
4976	SCGB3A2	7100731	1.06	1.10E-04
22202	PADI4	160187	1.06	1.11E-04
1064	DEDD	1340360	0.95	1.11E-04
12848	ASPRV1	5260392	1.05	1.11E-04
24640	VPRBP	6760593	1.08	1.13E-04
1374	ITGAE	520196	1.06	1.13E-04
10571	OLFR141	2630474	0.95	1.13E-04
12843	KLK1B11	7550519	0.94	1.13E-04
19710	HOXA2	7550451	0.94	1.14E-04
15445	LASP1	2190538	1.18	1.14E-04
17866	RNF34	4150431	1.06	1.15E-04
50	IL1RAPL2	840402	0.94	1.16E-04
1963	HEY2	5080468	1.06	1.17E-04
7694	ARHGAP25	3060215	0.96	1.17E-04
11368	TCTEX1D4	990669	0.95	1.18E-04
3344	FERMT2	1780377	1.17	1.18E-04
18236	MMP3	6180544	0.93	1.18E-04
24258	CHRNB3	780014	0.95	1.18E-04
23064	ZUFSP	6860709	1.06	1.20E-04
2350	TMEM147	6940014	1.11	1.20E-04
24596	PLOD1	1740504	1.08	1.20E-04
1275	NEIL1	3170246	1.05	1.22E-04
20033	DPH5	2190707	0.97	1.22E-04
24586	MEMO1	6130390	0.93	1.23E-04
8611	PPAPDC1A	4210064	1.06	1.25E-04
24641	KRBA1	1820470	1.07	1.27E-04
7515	GZMD	3390280	1.05	1.29E-04
7324	PLD3	6110037	1.07	1.30E-04
11576	MBNL3	450653	0.96	1.30E-04
1377	OLFR957	7650286	0.92	1.30E-04
20901	RUVBL2	2470386	0.90	1.31E-04
21933	SCN1B	940154	0.95	1.31E-04
9858	MYH6	4860743	1.06	1.32E-04
9949	1810011O1	7100639	1.05	1.35E-04
7882	ZRANB3	3140731	0.94	1.36E-04
23174	BMPR1A	460047	0.94	1.36E-04
622	RBM11	2510441	1.08	1.39E-04
21172	TMOD2	2030025	0.95	1.39E-04
10007	APBB1IP	3400079	1.05	1.41E-04
10430	ABLIM1	2230014	1.06	1.42E-04
23049	SLC9A6	2120224	1.05	1.42E-04

9691	TBX20	830392	0.93	7.78E-05
9899	ACYP1	1820576	1.06	7.89E-05
23668	NBR1	6020114	0.94	7.92E-05
10907	PPFIA2	3520136	0.95	8.00E-05
22980	GPSM1	4010228	0.88	8.00E-05
12828	MDGA2	2810349	0.89	8.04E-05
13562	UFSP1	1660731	1.08	8.06E-05
15577	PDCD5	3940392	1.08	8.06E-05
1720	ZSWIM4	7510240	0.93	8.07E-05
1404	KCNJ15	6180594	1.08	8.08E-05
22880	BMX	3180014	1.06	8.09E-05
8753	HPCAL1	4890735	0.93	8.15E-05
14288	MANSC1	1260041	1.04	8.15E-05
4853	CYP4F39	5570292	1.04	8.21E-05
13322	1810055E1	6550431	0.91	8.24E-05
4958	PRKCZ	5820681	1.08	8.29E-05
1233	TRPV1	1240278	1.06	8.41E-05
5650	CTPS	7100685	1.08	8.42E-05
12824	RWDD2B	770372	1.08	8.50E-05
19978	RORB	10132	0.95	8.52E-05
9032	PLN	1500162	1.05	8.61E-05
8759	MED8	6770681	1.10	8.66E-05
10043	1700073E1	650102	1.05	8.84E-05
16670	ESPN	4570519	1.05	8.86E-05
12279	9330182L0	7210128	1.04	8.88E-05
6669	GM1082	3800403	1.04	8.91E-05
4573	RAB11FIP5	6200333	0.95	9.01E-05
21324	ALPK3	4260274	1.09	9.05E-05
7581	TEX2	2760601	1.05	9.11E-05
450	AGBL2	4480097	0.93	9.11E-05
19318	V1RC16	10685	0.96	9.44E-05
11829	TMED4	580215	0.92	9.52E-05
6700	OLFR1089	1740592	1.04	9.53E-05
2545	CTRC	2320333	0.95	9.55E-05
4992	SF3A2	2190750	1.08	9.66E-05
1593	ZFP106	2680687	0.93	9.73E-05
3781	DDT	1990731	0.95	9.81E-05
13474	POLR2G	7330594	1.10	9.86E-05
5495	ATP6V1B1	5890075	0.96	9.86E-05
15864	HAMP2	7330482	1.06	9.87E-05
14370	CLSPN	6980315	1.05	1.00E-04
21485	NQO1	780301	0.95	1.01E-04
11731	H2-T10	4900722	1.05	1.01E-04
9579	ZFP652	10593	1.05	1.01E-04
9154	RPS6KA1	5860356	1.12	1.02E-04
24274	SETD8	1690114	0.94	1.02E-04
21466	COX6A2	2470356	1.16	1.05E-04
21073	PHEX	2340411	0.95	1.05E-04
6304	MSRB2	5960341	0.90	1.05E-04
8716	LST1	5860154	1.08	1.06E-04
14035	FZD3	1410025	0.96	1.06E-04
20581	OLFR96	1400471	0.95	1.07E-04
10301	EHMT1	130521	0.94	1.07E-04
22834	MED24	610040	0.87	1.08E-04
2436	TPD52L2	1990017	1.10	1.09E-04
9588	SNIP1	1580537	1.09	1.11E-04
2389	1700010M	2630646	0.96	1.12E-04
49	WDFY3	6250050	0.91	1.13E-04
22817	ALDOC	5550470	0.89	1.14E-04
9048	PATL1	360215	0.87	1.16E-04
10095	GATAD1	3290458	1.09	1.18E-04
4532	KIF18A	3990296	0.95	1.19E-04
9169	CDSN	60324	0.94	1.20E-04
5063	FIGLA	5860253	1.05	1.20E-04
329	TCFAP2A	5890735	0.95	1.22E-04
655	BCLAF1	4070392	0.96	1.22E-04
10832	SLC30A10	6770202	0.94	1.22E-04
280	ZBTB8OS	4040253	1.06	1.25E-04
19194	TUBB3	3130458	0.94	1.27E-04
12780	RELL2	4180152	1.14	1.28E-04
23519	PIM2	6020246	1.06	1.28E-04
7303	LENEP	5270678	0.96	1.28E-04
16439	POLD4	5910619	1.10	1.29E-04
23096	ABLIM2	6520408	1.07	1.30E-04
5963	HIBADH	770593	1.12	1.30E-04
13877	TNPO1	3060543	0.92	1.32E-04
21128	IGBP1	2470064	1.11	1.32E-04
4127	VIP	2940538	0.95	1.34E-04
11980	PLEC1	360014	0.93	1.35E-04
5609	NBL1	3850255	0.88	1.36E-04
2916	TRIP12	6280520	0.92	1.37E-04
4416	LNPEP	3390601	0.95	1.37E-04
15863	IFI202B	650601	0.95	1.37E-04
16561	SFXN4	7650243	1.05	1.38E-04
821	BCHE	2760327	1.07	1.38E-04
2501	MEST	3190408	1.05	1.39E-04
18554	1110006G0	4640743	1.07	1.40E-04
18433	ADAM23	3940286	0.90	1.41E-04
440	2010005H1	520035	1.04	1.41E-04

2361	PPARG	2320402	0.93	0.001
8162	D9ERTD402	2340390	0.90	0.001
20529	MRPL54	1070358	0.89	0.001
23128	LOC100041	7400400	1.06	0.001
16466	OLFR1273	7160519	1.04	0.001
6662	RFXDC1	1450446	0.92	0.001
15143	MS4A6B	3610286	0.93	0.001
3509	POLM	3440301	1.05	0.001
516	HOOK1	2000328	0.95	0.001
1265	6430527G1	1070187	0.94	0.001
4411	RABGGTA	4900692	0.94	0.001
12870	SLC38A4	5340386	1.04	0.001
545	ASCC3L1	5810170	0.94	0.001
3944	NR2F2	5860709	1.08	0.001
6293	RANBP17	4150086	1.07	0.001
21340	CEP72	5570047	1.06	0.001
2951	LTF	6940037	0.89	0.001
8465	FIP1L1	6760647	1.07	0.001
133	CLOCK	4810445	1.06	0.001

21287	GRAMD2	70435	1.05	1.43E-04
12902	CLCA5	1740167	0.94	1.45E-04
8621	ANKRD49	7150553	0.93	1.46E-04
6086	A830018L1	4890259	1.06	1.47E-04
16965	ESPN	6620544	1.04	1.51E-04
20773	CHRM2	2370259	1.05	1.52E-04
12404	IVNS1ABP	650168	1.08	1.55E-04
607	OTTMUSG0	6480152	1.05	1.56E-04
6902	PSP	2650338	0.96	1.56E-04
16424	ACTN3	4860577	1.06	1.57E-04
16847	AU017455	6520626	0.95	1.58E-04
7878	KCNH6	6760504	1.06	1.58E-04
16666	ZFP354B	2750563	1.07	1.58E-04
15261	TBPL2	6650484	1.06	1.58E-04
1603	CHMP7	5890390	1.07	1.60E-04
12794	PDCL2	4050192	0.94	1.61E-04
23317	1700018C1	5490215	0.94	1.61E-04
21765	CEP164	1190300	0.94	1.61E-04
5177	ZFP787	2140768	0.93	1.61E-04
3781	DDT	1990731	0.95	1.62E-04
287	TPRKB	3390538	1.11	1.62E-04
20000	ABCG8	650438	0.95	1.62E-04
629	NLRP1A	5860519	0.95	1.62E-04
18699	ENTPD8	5420482	0.97	1.65E-04
21069	AFMID	6040278	0.96	1.65E-04
22862	CES1	6480397	0.96	1.66E-04
12736	MXRA8	3940435	0.95	1.67E-04
12628	SP9	5390048	0.94	1.69E-04
16632	CKS2	6220309	1.03	1.69E-04
2879	KRT17	1780341	0.93	1.70E-04
8899	RPS6KC1	2750195	1.05	1.71E-04
20360	RNF17	7610379	1.05	1.72E-04
10980	MRPS10	7560465	1.05	1.73E-04
8554	UPK3B	4040243	0.94	1.75E-04
15363	ATPBD3	7000403	1.08	1.76E-04
6311	MAML1	7650674	1.05	1.79E-04
18239	LOC100047	840253	0.96	1.82E-04
8676	P4HA2	5960754	0.94	1.82E-04
639	DLX1AS	2970240	1.07	1.82E-04
16560	2810416G2	6580768	0.95	1.83E-04
20356	SCMH1	3710070	1.06	1.84E-04
100	PDE3B	4890411	1.06	1.87E-04
18722	THAP11	6760653	0.90	1.89E-04
21232	FAIM2	6980414	1.07	1.89E-04
4369	FGFR1OP	6100273	0.96	1.90E-04
144	EYA3	5220181	1.06	1.90E-04
619	1190020J1	7000114	0.96	1.90E-04
1265	6430527G1	1070187	0.94	1.91E-04
11396	ASNS	580670	1.05	1.92E-04
13585	WDR45L	130402	0.93	1.93E-04
23155	UGT2B36	840431	0.96	1.95E-04
2013	SPRR2F	2760669	0.96	1.96E-04
6402	N6AMT1	7380717	1.13	1.96E-04
18415	PRDX6-RS1	1940220	1.06	1.97E-04
23773	UBE2T	3190403	0.93	1.98E-04
7709	EG654453	6180259	0.95	1.98E-04
7339	HN1L	50609	1.04	2.00E-04
2059	GSS	3850167	1.06	2.02E-04
807	V1RD4	7210397	1.05	2.04E-04
3972	PLA2G2A	5050524	0.96	2.05E-04
3934	GPR61	7000619	0.95	2.06E-04
337	F9	3120403	1.06	2.11E-04
1960	A430107D2	670050	1.06	2.13E-04
5512	CAD	2760608	0.95	2.13E-04
8100	FPR3	4280291	0.97	2.14E-04
5037	STX1B1	70253	0.96	2.14E-04
7153	NFKBIE	990048	0.95	2.14E-04
14585	PTPN2	2140673	1.04	2.16E-04
20045	WDR41	3940692	1.08	2.17E-04
10116	4933407N0	6580274	1.06	2.18E-04
13254	PILRA	3520039	1.04	2.19E-04
9630	CCRL2	5130156	0.95	2.28E-04
12842	FBXO8	1450433	1.04	2.33E-04
13118	PKNOX1	3890139	1.06	2.36E-04
3289	BXDC5	20431	1.06	2.37E-04
24789	BC006779	6420170	0.95	2.37E-04
20825	WDR93	2190709	1.07	2.39E-04
16911	SLFN9	3890075	1.06	2.41E-04
9584	TMEM70	1990239	1.05	2.41E-04
12169	FTMT	3060730	0.96	2.43E-04
9698	CLCN6	3140162	1.05	2.43E-04
22298	THBD	1230767	0.95	2.46E-04
2370	AIM2	150095	0.95	2.47E-04
21446	5730419I0	1740020	0.91	2.47E-04
23651	FGL2	2340242	1.04	2.48E-04
4019	NPCD	4280692	1.08	2.48E-04
8407	PPP4C	6270735	0.94	2.50E-04
22691	ACVRL1	5860341	0.95	2.50E-04
19165	SNAPC3	6860082	1.05	2.51E-04

16320	DNAJA2	1690709	1.06	1.44E-04
13160	BC003236	6100719	1.05	1.46E-04
16436	GNAT2	4070259	0.95	1.46E-04
17542	HNRPH2	1170735	0.94	1.49E-04
5450	GLT6D1	7160451	0.96	1.49E-04
17609	MS4A3	7200142	1.06	1.50E-04
4680	DSTN	430332	0.87	1.52E-04
5348	SH3MD4	5670468	0.95	1.53E-04
517	NIP7	430484	1.11	1.55E-04
7332	BFSP2	4860427	1.05	1.56E-04
4612	2810046L0	2070736	0.91	1.58E-04
14500	RRP1B	6650431	1.04	1.59E-04
4002	A030013N0	6770639	0.95	1.61E-04
5117	EDAR	7510088	0.95	1.61E-04
11297	HDGFL1	5890156	1.05	1.62E-04
18317	SRM	3310347	0.90	1.62E-04
23567	OLFR166	5720703	0.95	1.63E-04
10261	KARS	3310379	1.10	1.63E-04
24072	OLFR1126	4290053	1.05	1.64E-04
8902	OLFR1061	6180176	1.06	1.65E-04
15403	LOC100047	5550348	0.91	1.67E-04
13522	USP5	6330746	1.06	1.71E-04
19759	WBP11	5870059	0.88	1.72E-04
7851	EPB7.2	6400148	0.95	1.74E-04
7008	EIF5B	940044	1.05	1.74E-04
10004	GATM	1690561	1.05	1.76E-04
3692	COX7A1	3360270	1.13	1.76E-04
20096	SEC63	5720082	0.92	1.77E-04
2032	NR5A1	3830039	0.94	1.77E-04
8058	EXOSC1	1240189	0.90	1.80E-04
20429	EG328839	2630307	0.95	1.81E-04
21525	OLFR876	7050543	1.05	1.81E-04
15530	CHMP1B	2900594	0.92	1.83E-04
2902	ZCCHC8	3710273	0.95	1.84E-04
17968	TUBA3B	2970630	1.04	1.85E-04
8907	CLEC1B	2710008	1.05	1.85E-04
2771	NNAT	2360541	0.94	1.86E-04
10736	SCPEP1	1030102	1.07	1.86E-04
18760	MEI1	2680682	1.04	1.89E-04
23160	PLXND1	4050079	1.06	1.89E-04
23811	AV249152	670537	0.95	1.90E-04
6203	ERMAP	1340176	0.96	1.91E-04
19095	ZFP87	1820307	1.06	1.94E-04
4317	MPHOSPH1	1710593	1.05	1.94E-04
889	RXFP4	4780156	0.94	1.96E-04
19072	OLFR378	2710692	0.96	1.98E-04
13765	TRAPPC3	4670224	1.10	1.99E-04
2166	ST6GALNA	6560692	0.94	2.00E-04
7582	LRMP	5390064	1.05	2.01E-04
2961	UPB1	4040376	0.96	2.02E-04
16957	TSPAN3	6840411	0.97	2.04E-04
19154	MMP7	7650735	1.05	2.05E-04
14253	LRTM2	1300609	1.05	2.06E-04
1766	ARHGAP30	4250544	1.04	2.06E-04
1174	TMEM183A	2750673	0.94	2.07E-04
18596	BMP2K	5560435	1.04	2.07E-04
13134	MSR2	6130746	1.07	2.08E-04
25190	MS4A1	1780187	1.05	2.12E-04
4632	PACSIN1	2190241	0.92	2.12E-04
1405	CRBN	4860064	0.93	2.13E-04
2590	OLFR961	1850370	1.06	2.14E-04
2606	RBM4	6590204	1.08	2.18E-04
14391	GM776	2140446	0.93	2.22E-04
23856	KLKB1	6060326	0.95	2.23E-04
2140	V1RH5	6590386	0.96	2.32E-04
25315	FOXD2	5260291	0.95	2.33E-04
16573	RPTN	2230288	1.05	2.33E-04
18711	SLC12A4	5360072	1.05	2.34E-04
19281	FEM1B	5090017	0.89	2.35E-04
11605	EG434280	2190184	0.95	2.35E-04
14996	RIPK5	2030093	0.95	2.37E-04
17979	WIZ	6550356	0.94	2.38E-04
19228	DDX3Y	6510477	0.86	2.38E-04
7945	NUP153	7550377	0.95	2.38E-04
397	ASPH	7210110	1.05	2.39E-04
13288	TWIST2	6420739	1.06	2.40E-04
10093	KCNS1	3310646	1.09	2.40E-04
17888	C8A	4810402	0.94	2.42E-04
722	SAPS3	460113	0.88	2.44E-04
27	LCORL	2470343	0.96	2.45E-04
1874	SLC44A2	2190632	0.93	2.48E-04
22561	SPCS3	3780717	1.11	2.50E-04
15478	CD28	7320161	1.05	2.53E-04
24592	TTLL1	7100180	1.07	2.55E-04
17385	9130218O1	3520278	0.95	2.57E-04
6141	RPE	610377	0.91	2.58E-04
10375	MYLPF	130349	1.06	2.59E-04
25189	SIRT3	5550554	1.21	2.60E-04
64	BC057371	5090653	1.05	2.61E-04

22227	1810029B1	6760291	1.06	2.52E-04
1913	HEMGN	6450576	0.96	2.54E-04
8740	ADARB2	3710431	1.07	2.55E-04
3253	OLFR568	3450398	0.97	2.55E-04
16902	OLFR1515	1570484	0.96	2.55E-04
1897	SLC7A2	50722	0.94	2.57E-04
3186	KATNAL2	3060750	1.07	2.58E-04
5087	H2-EB1	520072	1.09	2.61E-04
9102	PADI1	1690201	0.97	2.62E-04
13324	LOC100047	1780053	0.89	2.64E-04
19924	OLFR49	3290286	0.93	2.64E-04
20422	CCL20	4290523	0.92	2.68E-04
8311	PDGFB	7320500	1.05	2.68E-04
3996	VIM	1690091	0.90	2.69E-04
803	PEX3	3870349	1.17	2.69E-04
1397	LOC433632	6350397	1.05	2.73E-04
5294	CCDC42	1740746	0.97	2.74E-04
14005	A530098C1	1190368	0.96	2.74E-04
2443	GSTP1	4290040	1.03	2.76E-04
1908	TCP11L1	3870646	0.95	2.81E-04
1826	STXBP3A	6280066	1.06	2.81E-04
21859	UTS2	7380010	1.04	2.82E-04
16769	CYHR1	5670373	1.06	2.85E-04
21107	ALDH9A1	5050603	1.04	2.85E-04
2664	MAP3K8	2970598	0.95	2.87E-04
16090	CRYAB	5310598	1.19	2.88E-04
23542	CCDC50	5360653	0.94	2.90E-04
13399	PLCD3	1240039	0.93	2.92E-04
9549	ASAH3L	1470386	1.14	2.93E-04
20268	PTCD3	990326	1.05	2.94E-04
20371	BSND	5220451	0.95	2.95E-04
8303	CDC25B	2470360	1.06	2.99E-04
4875	DKKL1	7200600	0.94	3.03E-04
20779	SOX6	1300717	1.04	3.04E-04
18868	ADAM8	7200037	1.05	3.08E-04
4894	ACPL2	6060630	0.94	3.09E-04
2542	SLC2A5	450020	0.94	3.10E-04
5150	HCFC1R1	1030768	0.96	3.12E-04
14120	GRIA1	4830670	0.87	3.13E-04
10200	AKR7A5	3180068	0.93	3.17E-04
8634	SLC35C2	10204	0.90	3.18E-04
17685	POLR3A	6130646	0.93	3.19E-04
17708	STMN1	3120392	0.93	3.19E-04
6843	E130319B1	6550070	1.05	3.20E-04
24601	KRT73	3190402	0.94	3.20E-04
545	ASCC3L1	5810170	0.93	3.23E-04
22574	CXCL12	1470148	1.04	3.26E-04
7295	ITPR2	5820291	0.95	3.28E-04
4122	IFT81	5080347	1.06	3.29E-04
15794	HTRA1	6220703	1.11	3.32E-04
20275	RAP2C	3460053	0.94	3.38E-04
2503	IGSF21	6860537	0.89	3.39E-04
11443	LOC677205	1170438	1.06	3.40E-04
19056	DTNA	7380255	1.06	3.43E-04
488	3110050N2	610129	1.09	3.44E-04
15848	2310014L1	3840475	0.96	3.45E-04
5716	SCARA5	160377	0.94	3.46E-04
17280	GM467	2350497	0.95	3.47E-04
19401	BLK	3140491	0.92	3.48E-04
876	APOC2	5390240	0.96	3.49E-04
24255	TM9SF3	2350678	0.93	3.53E-04
4381	ZBTB9	3130072	0.95	3.55E-04
13860	LOC100047	3460674	1.03	3.56E-04
24458	GNMT	1110288	0.93	3.61E-04
14007	NANOG	5090661	0.96	3.62E-04
18950	ALB	7400647	0.92	3.62E-04
25519	ADD2	1230253	0.96	3.67E-04
17622	1700025D0	4780719	0.96	3.69E-04
17475	C1QTNF3	5260672	0.95	3.72E-04
3865	USP9Y	4880397	1.05	3.73E-04
11270	9630028B1	4220458	1.07	3.75E-04
17050	CENPI	6110646	0.96	3.76E-04
2916	TRIP12	6280520	0.93	3.83E-04
5777	PKP3	5260731	0.95	3.84E-04
18173	MCF2	7570397	1.05	3.84E-04
1886	MFSD8	7150670	1.05	3.87E-04
5334	HMGB2	5290279	0.95	3.88E-04
10468	TNFSF12-T	6100220	0.93	3.91E-04
3932	PLEKHA7	5690368	0.94	3.92E-04
1254	CADPS2	1090220	1.08	3.93E-04
21056	BC061039	6280086	1.05	3.93E-04
10606	ZFP609	7320687	0.95	3.94E-04
18605	AY074887	2450020	1.04	3.94E-04
13833	IGHMBP2	3060739	1.04	3.95E-04
5538	TMPO	5390494	0.96	3.95E-04
11402	GZMM	50253	0.94	3.96E-04
21000	WRNIP1	4670619	1.06	3.97E-04
13026	GNAS	1090132	0.94	3.98E-04
23921	MAZ	5560372	1.08	3.98E-04

15914	NDUFS2	4180379	1.06	2.63E-04
5622	CCL25	1780307	1.07	2.65E-04
7657	CLTA	4060491	0.95	2.68E-04
1266	GPR45	7040114	0.95	2.69E-04
14053	TAS2R143	3370561	1.05	2.71E-04
1895	DUSP23	670681	1.08	2.71E-04
915	FNDC7	7150133	1.04	2.73E-04
10621	SNCAIP	840148	0.95	2.75E-04
3568	HSPA4	4050630	0.96	2.77E-04
15235	SRA1	2060072	1.09	2.81E-04
978	GABPB1	4070598	0.94	2.82E-04
21796	2310037I24	2940152	0.91	2.84E-04
6139	V1RD17	6270129	1.05	2.84E-04
21994	PCSK1N	3610039	1.16	2.86E-04
1158	D6WSU163	4010129	1.06	2.86E-04
10948	REXO1	3450228	1.04	2.87E-04
6641	STAB1	2490286	0.94	2.91E-04
17778	SLC26A9	3130228	1.05	2.91E-04
18225	REEP1	4760630	0.87	2.93E-04
9726	EFTUD1	2680332	0.94	2.94E-04
24216	LIPH	380332	1.04	2.95E-04
8824	HUS1	1570079	1.09	2.96E-04
296	RFFL	6860398	1.05	2.96E-04
10330	KCTD10	2060612	0.97	3.00E-04
4859	RAB37	2000056	0.93	3.00E-04
8835	WNT2	4830008	1.08	3.01E-04
9463	ZFP288	4670280	1.08	3.02E-04
477	ADCK2	2370102	1.04	3.02E-04
7015	GNPTG	4890343	1.07	3.02E-04
4540	CST10	2140349	1.07	3.03E-04
3738	BOLA3	1430176	1.13	3.03E-04
26	TNFSF11	2480255	1.07	3.04E-04
7318	NSMCE2	2680180	1.05	3.05E-04
178	CETN4	6980022	0.93	3.05E-04
3915	MTAP	4050328	1.05	3.08E-04
8831	SNUPN	6840523	1.08	3.08E-04
5605	DMN	1740364	0.90	3.10E-04
6633	PFDN1	1660356	0.96	3.10E-04
685	4732418C0	620730	1.07	3.13E-04
19859	PSG19	4290482	1.07	3.15E-04
4838	GLT8D1	2190687	0.92	3.15E-04
716	CMYA1	4900279	0.96	3.18E-04
10132	ADRB3	7560349	1.06	3.20E-04
25623	PNKD	3460731	1.05	3.21E-04
12720	PRR19	730440	1.04	3.21E-04
1911	EFNB3	1570433	1.07	3.21E-04
1815	CREB1	130707	0.96	3.25E-04
945	RAD18	4390632	0.95	3.26E-04
12519	URM1	2450678	1.11	3.28E-04
211	ARFGAP1	6100537	0.95	3.28E-04
6936	PCYOX1L	3870092	1.06	3.29E-04
23089	TMEM53	990239	0.94	3.32E-04
25391	LOC100046	2350286	0.93	3.33E-04
15283	FMOD	6110520	1.04	3.34E-04
10277	EPB4.1L1	7400491	1.13	3.35E-04
21015	MFGE8	2120286	1.13	3.36E-04
20749	ENDOGL1	3310368	1.08	3.38E-04
7609	BRUNOL4	3290670	0.92	3.41E-04
1775	LOC433721	2510541	1.06	3.42E-04
1443	CRLS1	3450154	1.06	3.43E-04
25602	TAF1C	60255	1.04	3.56E-04
25358	RBM10	1980554	1.05	3.57E-04
14491	ANXA13	3610521	1.05	3.57E-04
13225	SLC35F3	5130424	0.91	3.58E-04
3805	APEX2	4050475	0.96	3.59E-04
1516	LRRC39	2690524	1.05	3.60E-04
23450	SOHLH2	1240369	1.05	3.62E-04
34	D030070L0	1510433	1.10	3.62E-04
90	EG317677	2710465	0.94	3.63E-04
1399	D4ERTD22E	4920564	1.07	3.64E-04
2109	JMY	5820707	0.95	3.67E-04
8893	MAGEA4	7550131	0.95	3.67E-04
1629	SLMO2	6660497	0.92	3.69E-04
13534	LOC100041	5870630	0.96	3.74E-04
10002	CYCS	830056	1.06	3.78E-04
14242	DOK5	2370767	1.09	3.80E-04
15743	WDR4	3310242	1.05	3.80E-04
17832	DSG1B	6510068	1.05	3.83E-04
16297	GM93	4220707	1.05	3.84E-04
87	HBB-B1	670403	1.09	3.85E-04
6374	FADD	6450050	1.07	3.86E-04
10858	FBN1	110048	1.06	3.88E-04
18098	DGKQ	1710474	0.95	3.89E-04
21337	HTR4	4850538	0.93	3.95E-04
3916	RBM12	4290019	0.95	3.96E-04
15261	TBPL2	6650484	0.95	3.97E-04
641	RNF14	1430711	0.93	3.97E-04
5197	4933433P1	1300706	1.12	3.98E-04
19451	SARS2	2060047	1.07	3.99E-04

23709	MDGA2	290280	0.95	3.99E-04
22113	E130014J05	2450670	0.93	4.00E-04
17688	LTB	160408	0.94	4.03E-04
3242	PCTK2	1740634	0.97	4.04E-04
11139	RCOR1	110154	1.06	4.07E-04
16340	RFTN2	6590243	1.10	4.10E-04
9582	RBBP9	2230424	1.09	4.11E-04
1696	CAMK2D	1580397	0.96	4.12E-04
5053	ARRB1	1340239	1.04	4.19E-04
17940	PKD1L2	6400402	0.96	4.19E-04
687	SRC	3120709	0.94	4.19E-04
20894	OLFR1249	3060722	1.04	4.21E-04
13517	CCKAR	7210546	0.97	4.22E-04
5057	TREML4	4670162	1.05	4.23E-04
7163	BC050196	7320632	0.92	4.23E-04
4388	HIF3A	4290201	1.04	4.24E-04
24934	CCRL1	5890091	1.06	4.25E-04
22749	PYROXD1	4180524	0.94	4.25E-04
8218	UBE2M	2060450	1.08	4.26E-04
3665	PRH1	6480170	0.95	4.28E-04
22576	WDR67	6110324	0.95	4.28E-04
14661	TRPC5	6840653	0.94	4.29E-04
16580	LOC100047	4290653	1.14	4.40E-04
7121	PIGR	6510373	0.94	4.42E-04
3743	V1RD19	1690367	0.97	4.43E-04
3118	BARD1	2760609	0.93	4.43E-04
5027	SKIV2L	60707	1.06	4.44E-04
22418	2210412D0	50608	0.96	4.44E-04
18433	ADAM23	3940286	1.13	4.49E-04
21298	NPM1	6840433	0.90	4.53E-04
3756	GTPBP8	5870180	0.94	4.53E-04
9508	PRX	5050093	0.96	4.60E-04
10926	FBXW8	7200626	1.07	4.60E-04
3748	PPP1R3B	4150097	1.06	4.61E-04
27	LCORL	2470343	0.92	4.62E-04
25525	NCKIPSD	4670121	1.08	4.64E-04
37	WDR31	5130082	0.94	4.64E-04
8223	CCDC96	7040315	1.04	4.73E-04
15187	PTRF	2320014	0.96	4.73E-04
12078	DHRS7	3440639	1.07	4.79E-04
3809	HSD3B2	7550575	0.97	4.79E-04
22712	NPFFR2	4890537	0.94	4.80E-04
23027	CCDC97	6620528	1.06	4.82E-04
20468	3000004C0	6350367	0.94	4.83E-04
10933	AKAP6	6510259	1.06	4.85E-04
6762	RG9MTD2	1110072	1.06	4.89E-04
678	ZFP113	830348	0.91	4.95E-04
15837	SCRIB	840066	0.95	4.98E-04
22644	E530011F1	4900170	0.95	5.04E-04
11253	ARMC8	270524	1.04	5.13E-04
24639	DUSP9	4920612	0.95	5.13E-04
13726	ZFP105	670039	1.04	5.13E-04
10773	HEATR5A	6520040	0.96	5.15E-04
3635	LOC100045	130500	0.95	5.15E-04
14545	HIRA	5290431	1.10	5.17E-04
24086	PLEKHG4	520719	0.95	5.17E-04
3337	GPC3	3890500	0.95	5.19E-04
17893	PMM2	110474	0.96	5.21E-04
3291	NDRG3	4070377	0.88	5.25E-04
17664	TRAM2	4210193	0.97	5.28E-04
1719	ZFP273	7400600	0.95	5.29E-04
3381	PPAPDC3	5910446	1.09	5.35E-04
4116	TGFB1	5130139	0.95	5.36E-04
1844	COPZ2	4920056	1.06	5.45E-04
12526	KRAS	1820088	1.05	5.45E-04
18204	ZFP108	460543	1.05	5.48E-04
808	PHF6	6100646	1.07	5.54E-04
115	KLHL28	450594	0.96	5.58E-04
17705	EDF1	1850477	1.19	5.60E-04
17955	RPO1-4	6380370	1.06	5.60E-04
25141	TMEM49	2340113	0.96	5.66E-04
10338	NFASC	6060563	1.07	5.67E-04
24442	HIPK4	2350546	1.05	5.67E-04
20263	ZFP59	110072	0.95	5.71E-04
19626	EXOSC2	1580307	0.89	5.81E-04
18705	MEGF10	4480373	1.10	5.83E-04
425	GPR162	5260092	0.93	5.83E-04
1089	ZFP771	4210278	0.94	5.84E-04
25611	CHMP2A	540156	1.06	5.89E-04
8225	BC053749	3290379	1.07	5.92E-04
23289	TMEM115	5900379	0.91	5.97E-04
1934	KIF15	7650538	1.04	5.99E-04
6649	PPP1R2	2850192	0.92	6.08E-04
9045	HSBP1	7510288	0.89	6.12E-04
5708	BC087945	5720128	0.95	6.13E-04
24345	TOR1AIP2	4760255	0.97	6.14E-04
6223	RANBP1	1940348	0.91	6.16E-04
24056	KRT14	1470619	0.97	6.17E-04
5585	V1RC13	4570017	0.94	6.17E-04

14073	MUC1	1260047	0.94	3.99E-04
3087	ETFA	5960653	0.92	4.00E-04
1323	AAMP	4050470	0.86	4.00E-04
10936	4933407P1	3440722	0.96	4.00E-04
138	CYP4F16	540075	0.94	4.05E-04
25646	4930401F2	4900717	1.04	4.05E-04
5696	CAR5B	7200528	1.06	4.06E-04
6029	SLC7A13	7050474	0.95	4.07E-04
2631	TAF9	3850458	1.08	4.09E-04
15100	CHRNA4	2070482	0.94	4.11E-04
5965	PTPRK	1740561	0.95	4.12E-04
11006	SUCLA2	4480477	0.94	4.14E-04
158	RAB17	2850349	0.96	4.14E-04
1288	BTG3	7150433	1.05	4.17E-04
21540	OLFR736	6250156	0.95	4.19E-04
1592	HNRNPR	6650204	1.05	4.19E-04
18033	CLEC11A	2690411	1.08	4.20E-04
22358	SMOX	5050008	1.11	4.20E-04
1628	ACSM5	4850739	0.95	4.21E-04
9426	8030462N1	6220086	1.04	4.28E-04
753	ARHGAP27	5130438	1.07	4.31E-04
6124	FBXO34	6380148	1.12	4.33E-04
21075	GSDMA1	3800187	1.05	4.42E-04
16167	BC024561	6620176	1.05	4.42E-04
11276	POLR2A	450609	0.93	4.43E-04
13039	ERRFI1	4570368	0.91	4.44E-04
11594	TNFAIP1	3940520	0.89	4.46E-04
18502	5730410I1	10270	1.06	4.51E-04
19294	CPA6	5810026	1.04	4.51E-04
16802	ATRX	450400	1.04	4.54E-04
11518	RHOX5	4390148	0.96	4.54E-04
23076	3110004L2	1260468	1.05	4.56E-04
16521	STX6	3840202	0.91	4.57E-04
12515	V1RH12	4260482	0.94	4.64E-04
1532	2810003C1	3610239	1.10	4.64E-04
19769	1110039B1	3710286	0.94	4.67E-04
15503	OLFR366	1660019	1.04	4.69E-04
12934	BCAR1	1770189	1.06	4.69E-04
6623	4921528G0	7050390	0.95	4.70E-04
21131	ZFP423	2600402	1.07	4.70E-04
3633	SPRED3	7050020	0.95	4.77E-04
20190	D11BWG05	5700669	0.90	4.78E-04
10763	TYMS	510544	1.04	4.79E-04
18721	NKIRAS1	1070195	1.04	4.82E-04
9664	GPR56	60079	0.94	4.86E-04
18952	RAB8A	1240324	1.08	4.88E-04
16848	OGT	6960095	0.93	4.91E-04
1384	DMD	360367	0.92	4.94E-04
7974	MTNR1B	7320100	1.05	4.99E-04
14644	TMEM180	1690685	0.92	5.01E-04
24855	1600029D2	1300092	1.05	5.01E-04
20493	KLHL6	1780445	1.06	5.03E-04
441	NUP37	4290594	0.94	5.05E-04
10109	YIPF6	6290575	1.06	5.18E-04
7150	V1RC3	4810020	0.96	5.22E-04
19481	ATP8B3	130020	0.95	5.22E-04
10107	SOCS1	5670497	1.08	5.25E-04
5330	OLFR1380	940468	0.95	5.25E-04
2803	ZFP523	6510427	1.09	5.25E-04
10893	HTR5A	2060400	0.94	5.27E-04
15915	MXI1	1050553	0.92	5.27E-04
1483	RIPPLY1	3290692	1.04	5.28E-04
1533	PMP22	4730685	1.09	5.28E-04
23970	DDX19B	3610482	0.90	5.29E-04
3418	MECR	1990730	1.08	5.36E-04
15850	LOC268782	1570661	1.06	5.37E-04
23313	E330016A1	2350221	0.96	5.47E-04
10103	GIT2	3450372	1.07	5.49E-04
13223	ANP32B	6550707	0.96	5.51E-04
19747	NNT	3840102	1.07	5.52E-04
22269	V1RD3	2630050	0.95	5.58E-04
13018	BC004728	2690368	1.08	5.60E-04
5875	OLFR944	1990300	1.06	5.61E-04
11451	ORF9	4810259	1.06	5.62E-04
21010	MYCBPAP	4640292	1.03	5.63E-04
7908	RNF5	2450224	1.10	5.64E-04
24651	REEP5	730541	0.86	5.64E-04
19090	1110049F1	780332	0.93	5.70E-04
2034	GORASP2	5910601	0.94	5.71E-04
22009	FKBP2	1050082	1.10	5.73E-04
17511	ELMO2	5050465	1.08	5.73E-04
11811	MEN1	4120593	0.91	5.74E-04
22983	6430510M	2600068	0.87	5.74E-04
19491	CENPQ	6480292	1.04	5.75E-04
6815	ZC3H12B	2710368	1.06	5.78E-04
7841	MEX3A	6290367	1.04	5.80E-04
284	OLFR1497	4290626	1.05	5.82E-04
1787	MUG4	5870010	0.97	5.82E-04
909	OLFR144	5080440	0.97	5.83E-04

1706	2610507B1	4200674	1.07	6.18E-04
10842	TAZ	5130154	0.94	6.20E-04
128	TRPC4	3060398	0.96	6.21E-04
1206	BC017612	7380048	0.96	6.23E-04
22163	EIF5	5490114	0.91	6.24E-04
10356	4930404H2	2900162	1.05	6.25E-04
15568	1110034A2	10707	1.05	6.28E-04
784	ATIC	4830484	1.05	6.28E-04
16361	OLFR1013	4060358	0.96	6.33E-04
997	4921530G0	5900465	0.93	6.36E-04
25166	RPS4Y2	6590008	0.92	6.40E-04
21247	ACAD9	3170301	1.11	6.41E-04
21211	COL4A3BP	1260440	0.96	6.47E-04
70	FGFR3	7200035	1.12	6.49E-04
10970	TUBA3A	4180743	0.96	6.51E-04
19021	F730047E0	4670601	1.05	6.53E-04
5622	CCL25	1780307	1.07	6.57E-04
636	KBTBD8	650672	1.06	6.75E-04
10774	MAFB	6060403	1.05	6.78E-04
1759	STX3	5820601	1.05	6.84E-04
14268	WNK4	1850255	0.94	6.86E-04
8141	PPM1D	2320537	1.09	6.88E-04
9933	NUT	3180091	1.05	6.89E-04
11855	RBMS2	1500647	1.05	6.89E-04
13198	KCNQ3	4570546	1.04	6.89E-04
12162	SALL1	430050	0.95	6.91E-04
17874	ATP5J	4860673	0.96	6.95E-04
536	PDE6D	1010730	0.96	6.96E-04
15492	SPEN	3460605	0.94	6.97E-04
667	LOC637082	60626	1.04	6.98E-04
18398	MBD1	1110338	0.92	6.99E-04
17098	SMARCA2	1580327	1.09	6.99E-04
5306	OLFR608	6940204	1.07	7.00E-04
12824	RWDD2B	770372	0.94	7.00E-04
10858	FBN1	110048	1.06	7.04E-04
10995	CCDC130	160148	0.94	7.04E-04
232	OLFR482	3800292	0.94	7.07E-04
15070	B4GALT3	360102	0.93	7.11E-04
2280	SRPX2	2190139	0.97	7.12E-04
18109	AI662250	1070156	0.94	7.21E-04
18136	MTDH	2850022	0.95	7.21E-04
5157	TIGD3	2060228	1.05	7.26E-04
1511	PDCL	20722	1.09	7.30E-04
4406	TBC1D2	5550482	1.05	7.33E-04
13552	TMEM140	2690379	0.96	7.34E-04
2315	CAPN2	4390020	1.07	7.35E-04
22609	TDPOZ3	5490544	0.95	7.36E-04
20007	FBXO8	5890730	0.95	7.36E-04
8769	FOXP2	2940070	0.95	7.46E-04
4943	EG630579	4640204	0.96	7.48E-04
25427	1700007G1	3310274	1.05	7.55E-04
12070	MAN1C1	5700747	1.05	7.55E-04
300	SART3	3800079	0.96	7.56E-04
7970	D17H6S53E	3520255	1.05	7.56E-04
16855	TLR11	2490072	0.95	7.57E-04
16395	JUN	7560291	1.11	7.57E-04
8309	LMCD1	2710347	1.10	7.59E-04
13716	DHRS4	2510689	0.94	7.61E-04
21442	OLFR1431	5050048	0.96	7.62E-04
17368	SERINC3	1770201	0.89	7.65E-04
15348	CRAT	5890102	0.95	7.67E-04
13086	AA408296	4920746	1.07	7.75E-04
3202	NIPSNAP3A	630162	0.96	7.75E-04
6610	C1QDC2	870291	0.94	7.80E-04
15949	TRIP4	3850603	1.06	7.83E-04
3264	OLFR559	1050397	1.03	7.84E-04
10629	CSPG4	5690553	1.06	7.85E-04
4777	CTNNAL1	2320168	1.07	7.88E-04
19971	RSF1	6220332	0.95	7.91E-04
4851	DDX3Y	6520685	0.96	7.91E-04
20869	DST	5360021	0.95	7.95E-04
3359	ZP2	1450129	1.05	7.95E-04
14293	BNIP1	50681	1.07	8.00E-04
5522	DMRT2	6770161	0.94	8.03E-04
21156	RAN	1050097	1.09	8.05E-04
25445	OLFR50	5220433	1.05	8.06E-04
12664	LCTL	4290358	0.97	8.07E-04
10530	RPS27A	5550431	1.05	8.10E-04
214	NDUFA5	6560152	1.07	8.11E-04
6500	RSRC1	1010487	0.93	8.12E-04
1566	AU018829	4890059	1.06	8.20E-04
6726	USPL1	3520274	0.94	8.29E-04
16610	PPP2R5C	2760707	0.93	8.29E-04
17003	ACTG2	1980209	0.96	8.30E-04
7848	OLFR1008	7200647	0.95	8.32E-04
9193	AB041803	3370189	1.04	8.37E-04
18647	COL17A1	6770369	0.95	8.40E-04
7726	TIAL1	6650148	1.03	8.42E-04
24028	WDR34	2690224	0.93	8.45E-04

23696	PECAM1	5670020	1.07	5.85E-04
2919	MR1	4260292	1.07	5.86E-04
33	OPN5	3180209	0.92	5.86E-04
21172	TMOD2	2030025	0.96	5.89E-04
12817	1700109H0	2760239	1.05	5.90E-04
3679	BC021381	1240575	1.05	5.94E-04
19802	MYOC	7400463	0.89	6.01E-04
12812	APCS	2320170	1.06	6.06E-04
7673	OAS1A	3290767	1.05	6.09E-04
6945	LOC216443	5420367	1.07	6.12E-04
10574	MS4A6D	3180025	1.06	6.15E-04
19041	KCNMB4	4180491	1.11	6.17E-04
4171	CSN2	450376	0.95	6.17E-04
23186	GOSR1	3850138	1.06	6.17E-04
14930	GNA15	3310577	0.95	6.18E-04
21748	OLFR681	6220278	1.03	6.36E-04
6042	B3GALT2	3780246	1.05	6.37E-04
20412	BGN	2100402	0.88	6.38E-04
10737	40972	3420026	0.93	6.44E-04
429	MICAL1	1850326	0.90	6.44E-04
24167	HCRTR2	3520743	1.06	6.48E-04
1536	CANT1	5900594	0.94	6.52E-04
372	CYP11B2	4760246	1.06	6.61E-04
14342	ZFP52	4900575	0.96	6.62E-04
10041	RLBP1L1	1070450	1.05	6.62E-04
22716	KCNK6	6510026	1.04	6.69E-04
24627	CRAT	160121	1.10	6.73E-04
588	EIF4G2	6520484	1.15	6.75E-04
8461	1100001E0	6200685	1.04	6.75E-04
24186	CORO7	6840092	1.04	6.81E-04
5692	LANCL1	1580762	1.12	6.82E-04
23343	BC003993	4290537	0.96	6.83E-04
8393	DEFB19	2030088	1.05	6.83E-04
3402	GJC2	610112	1.07	6.85E-04
24457	CCND2	1580088	0.86	6.88E-04
25162	ORM1	60196	1.05	6.92E-04
598	MYLC2B	6100021	0.92	6.93E-04
70	FGFR3	7200035	1.11	6.96E-04
15381	LRRC1	5810647	0.96	6.98E-04
11434	CDC26	5560687	1.05	7.04E-04
24803	SP2	5220368	0.95	7.06E-04
2351	PGK2	4920021	1.06	7.11E-04
10106	MAN1B1	6100110	0.94	7.23E-04
22696	TEX9	730546	0.94	7.28E-04
22484	MUC13	4880386	1.07	7.30E-04
10660	MAPK12	2690392	0.96	7.31E-04
217	WIPI2	2190612	0.91	7.41E-04
16377	AKAP1	5290670	0.96	7.44E-04
16804	CTDSP2	6520468	0.97	7.46E-04
9994	PTP4A2	6180309	1.09	7.56E-04
24894	RASGEF1B	3370575	0.96	7.57E-04
21256	HIRA	7610661	0.94	7.62E-04
4473	HSPB1	5670722	0.95	7.64E-04
20162	9130005N1	450397	1.05	7.73E-04
11	OLFR187	380370	0.92	7.79E-04
6325	EN2	6940544	0.93	7.80E-04
2114	INOC1	4880121	0.97	7.83E-04
4542	2410004A2	3870717	1.05	7.83E-04
1748	NAT2	1710451	1.06	7.83E-04
12474	PTK9	3990682	0.91	7.86E-04
25214	DEPDC7	1660358	1.06	7.95E-04
12547	2310067B1	5820142	0.93	8.00E-04
8235	SHCBP1	3830397	1.04	8.00E-04
224	4933434I2(	6100497	1.07	8.02E-04
15708	SCYL1BP1	4010564	1.03	8.02E-04
12861	LRRC23	670386	0.94	8.11E-04
4116	TGFB1	5130139	0.93	8.17E-04
13251	MBP	1450646	1.14	8.18E-04
15574	HMX1	4220300	1.06	8.19E-04
3868	NOXO1	2370672	1.07	8.21E-04
5472	D0HXS9928	5090537	1.05	8.21E-04
17883	4931432M	270152	0.97	8.23E-04
15011	AFF4	7100603	0.94	8.29E-04
5136	DNAHC5	4860270	0.96	8.33E-04
24851	MARK4	4480445	1.05	8.34E-04
11593	SIAH1B	3190376	0.93	8.35E-04
4888	BC026585	2900661	1.09	8.36E-04
8321	BC004044	3290253	0.90	8.37E-04
19539	DDX3Y	4120196	0.95	8.38E-04
1276	NFATC2	2000725	1.05	8.41E-04
14141	CSNK1G1	2710437	1.05	8.42E-04
25479	NR2F6	50431	1.05	8.43E-04
10750	EG624219	3520095	0.93	8.48E-04
22587	PIK3IP1	940180	0.94	8.55E-04
22728	RGS3	1230292	0.95	8.56E-04
1578	LOC100044	5050402	1.05	8.56E-04
2378	ANKLE2	5360598	1.05	8.64E-04
606	MEX3C	1010446	1.04	8.68E-04
1372	4831426I19	1430132	1.04	8.68E-04

10576	CCDC51	5360255	1.07	8.46E-04
2359	PROM1	2760167	1.07	8.50E-04
1441	1200016D2	20719	1.06	8.59E-04
4443	IFIT1	1780142	1.05	8.63E-04
8659	BUD13	5080041	1.06	8.63E-04
2627	RBM34	4250470	0.95	8.66E-04
17491	EFNA1	2490053	1.04	8.68E-04
6298	RPRML	2680523	0.93	8.74E-04
18412	ADPGK	5900601	0.92	8.75E-04
215	1810015A1	510338	0.92	8.75E-04
9971	H2-DMA	870154	1.07	8.76E-04
6581	PARK7	4730739	1.08	8.77E-04
2707	ACOX2	3290411	0.94	8.78E-04
1034	OLFR1094	5570270	0.94	8.81E-04
10661	HFE	2360672	1.07	8.89E-04
5003	GLT8D2	4860446	1.07	8.99E-04
42	RBM47	6270243	1.06	9.00E-04
4485	ZBTB7C	2260398	1.06	9.04E-04
14314	GM561	3440504	1.17	9.13E-04
4670	RRM1	6400348	1.06	9.19E-04
22198	MICAL3	4200592	1.06	9.19E-04
6967	NAB1	730736	0.94	9.24E-04
8114	SMAD3	2450053	1.08	9.24E-04
16905	TGFB3	2510500	0.96	9.25E-04
3080	CYP2C40	3850273	1.03	9.32E-04
10263	4930519G0	4900091	0.96	9.38E-04
11286	TCAM1	4640072	0.96	9.41E-04
22336	4833426J09	4040400	1.04	9.41E-04
16075	ROR2	6130743	0.96	9.43E-04
19213	LCE3F	1660754	0.97	9.43E-04
21221	SLC25A27	6420017	0.94	9.44E-04
624	OLFR1218	3440189	0.94	9.54E-04
24291	ABRA	7550066	0.96	9.55E-04
16271	6330503K2	3420521	1.08	9.83E-04
17432	ITGB5	1770152	1.10	9.87E-04
18516	WBSCR27	3930026	0.95	9.90E-04
17232	FADS6	7150475	0.95	9.90E-04
21867	SNAPC5	3290368	1.10	9.95E-04
1501	LOC100047	7510609	0.95	9.96E-04
15929	HDLBP	6180187	0.93	0.001
16950	SCNM1	2140048	1.04	0.001
21977	KIRREL2	1300022	0.92	0.001
5687	TRIB3	2370110	0.90	0.001
7885	V1RE12	4540687	0.94	0.001
19907	EIF1AY	2450402	0.96	0.001
19901	DPP3	4050372	1.12	0.001
24844	TRIM21	5670398	0.95	0.001
3854	ABPE	270520	1.04	0.001
5517	SS18	6100010	1.05	0.001
13091	MBNL2	3450719	1.12	0.001
19286	GUCY2C	4070719	0.95	0.001
25240	TMPO	10181	0.96	0.001
21787	PGPEP1	2490521	0.96	0.001
14903	ZBTB40	1980598	1.05	0.001
1781	NDUFB6	6480747	1.09	0.001
24017	HTRA3	780546	0.94	0.001
16352	WDR8	6290253	0.94	0.001
24820	ACTL6B	940386	0.92	0.001
503	INPP5E	3800753	1.05	0.001
3632	NOTCH1	6450022	0.97	0.001
4804	TNKS1BP1	6420114	1.06	0.001
9240	DCBLD1	1500768	0.94	0.001
14349	SERPINB7	580475	0.95	0.001
6600	DCTN1	2900154	1.04	0.001
11696	PAQR3	4210040	0.96	0.001
136	KRT222	150064	1.12	0.001
10638	NEUROG2	6200647	0.94	0.001
25245	SLC12A9	6480575	1.07	0.001
6209	ORMDL1	6400202	0.96	0.001
2099	KIRREL3	6280474	1.07	0.001
18174	5830405N2	2900307	0.95	0.001
20177	TCL1	2680438	1.04	0.001
4140	GPC4	5270491	1.07	0.001
22587	PIK3IP1	940180	1.07	0.001
5537	CYP4A12A	6620707	1.04	0.001
3219	FGD6	940487	0.93	0.001
6571	ATG2B	2070138	0.97	0.001
3822	TCFAP2C	3780711	0.95	0.001
13171	KRT27	6200671	1.04	0.001
3744	ZFP385A	6960523	1.07	0.001
17619	CDH23	5960731	0.95	0.001
2946	CASP3	2690307	1.04	0.001
17712	OLFR1101	620458	0.95	0.001
18761	5031408O0	6370504	1.07	0.001
15442	CD300LF	3170487	0.95	0.001
8393	DEFB19	2030088	1.05	0.001
10480	2310008H0	1770397	0.96	0.001
8044	UCP2	4920594	1.05	0.001
1842	RUNDC1	6250681	1.07	0.001

17676	1110008J0	2600605	1.06	8.68E-04
10908	TMEM47	6400634	0.90	8.70E-04
8997	KCNE3	4860184	1.03	8.76E-04
19725	ZFP449	6020300	0.95	8.83E-04
9547	CADM3	580673	0.89	8.84E-04
18470	ABCF1	2340246	1.05	8.89E-04
144	EYA3	5220181	1.05	8.92E-04
1103	RGS1	4830072	0.93	8.95E-04
16395	JUN	7560291	1.09	8.96E-04
9916	H2-D4	5050451	1.05	8.96E-04
24174	DUS3L	6370543	1.07	8.98E-04
1463	ABI3BP	2810576	1.06	8.99E-04
13599	APOC1	1710066	0.95	8.99E-04
16651	HIST1H4H	7400377	1.10	9.00E-04
5153	ZDHHC13	5560079	1.06	9.01E-04
4207	OLFR186	2120528	0.92	9.05E-04
12936	NDUFA8	4220519	1.10	9.16E-04
9264	LOC100044	6580711	1.10	9.19E-04
502	STK10	7610468	1.05	9.29E-04
23391	NPR2	1580129	1.06	9.30E-04
1614	ST6GALNAC	5670592	0.95	9.39E-04
5651	NLN	5670112	0.92	9.46E-04
13685	NUAK1	6860309	0.85	9.47E-04
7769	OLFR1356	5960307	1.04	9.49E-04
22645	RNF185	6900543	0.95	9.56E-04
13413	2610024G1	10019	1.10	9.59E-04
1573	OLFR23	2810201	1.08	9.63E-04
9370	TIMM9	610075	1.07	9.63E-04
13798	AIRE	6660367	0.96	9.63E-04
1606	HMGN1	1260274	0.88	9.63E-04
9817	4932417H0	5890327	0.93	9.66E-04
15870	GSS	1340020	1.06	9.74E-04
4569	PLEKHG5	6020327	0.92	9.74E-04
19630	CNNM1	5050246	1.05	9.78E-04
14684	BC066107	2810390	1.05	9.85E-04
10573	1300001I01	1050114	0.93	9.85E-04
20463	EVI1	4730427	0.95	9.87E-04
5944	V1RE2	2470519	0.94	9.93E-04
7800	ADAM17	5900653	0.94	9.96E-04
4158	BC013491	1940246	0.96	0.001
11440	PNRC2	2850441	0.92	0.001
1162	ATP6V1H	5220139	1.09	0.001
11721	PRR14	5810280	0.93	0.001
4240	RAET1A	5870543	0.96	0.001
25116	FBXL12	4150440	1.10	0.001
5480	AKP2	7200717	1.06	0.001
12381	JMJD3	4920424	1.06	0.001
12231	OLFR117	6270523	0.95	0.001
20179	CDC20	4570088	1.05	0.001
1522	GRSF1	4890598	0.88	0.001
24869	TRPM6	5700364	0.95	0.001
15714	RALGPS1	3170482	0.96	0.001
7787	RCSD1	6400255	1.05	0.001
2565	NRCAM	4830040	1.06	0.001
14892	0910001L0	6550632	1.10	0.001
20074	MYST1	6370674	1.07	0.001
5547	PDE1A	7210632	0.90	0.001
6661	GRIK1	2260189	0.97	0.001
10919	SDCBP	1770328	1.06	0.001
1907	9430028L0	6560010	0.92	0.001
3512	DSG2	1740082	1.05	0.001
9249	BGLAP-RS1	3450521	1.04	0.001
4602	XLR4C	1850382	0.96	0.001
11090	TPCN1	2850154	1.04	0.001
22280	ZHX3	2100253	0.96	0.001
18733	CDK8	4890228	0.97	0.001
1960	A430107D2	670050	1.09	0.001
1751	CACNA1C	6960630	0.96	0.001
9035	TLR13	7320020	0.91	0.001
4276	DHX30	1260692	0.91	0.001
21631	SERPINB9D	7610184	1.05	0.001
11214	PODN	3290301	0.95	0.001
22350	NMNAT2	6760091	0.95	0.001
21737	GALNT10	6400463	1.05	0.001
13532	RNASE9	2940682	0.97	0.001
737	SERPINB12	7650035	1.11	0.001
4300	PRNPIP1	6650215	0.90	0.001
860	ELMO1	1570239	1.05	0.001
7572	HERC3	2480243	1.05	0.001
6049	PRNPIP1	6860390	1.11	0.001
21842	PRPF38B	5090747	1.06	0.001
8639	ATP2B2	3170148	0.93	0.001
3395	AF366264	6480040	1.04	0.001
12915	FLCN	520039	0.96	0.001
15205	LTK	5340259	0.96	0.001
12080	NOL1	3830400	0.94	0.001
9859	BC046331	6270619	1.07	0.001
25357	NUBP2	540400	1.06	0.001
18471	AU040829	840360	1.07	0.001

14446	PPP2R5C	1240397	1.08	0.001
3133	DSCR1	3440615	0.94	0.001
10732	FBXW13	6130372	0.96	0.001
15666	CHRAC1	1500431	1.07	0.001
7704	MAFA	6400026	0.96	0.001
15544	SESN2	290669	1.05	0.001
23913	TRIM68	6650692	1.04	0.001
3309	SPNB2	650630	1.07	0.001
17459	9930022N0	5050086	1.05	0.001
15373	ATP2A3	6370594	0.95	0.001
727	D10BWG13	1510482	0.91	0.001
13112	SHPRH	3840678	1.04	0.001
14953	PPP1R16B	840397	1.05	0.001
4592	EZH2	580458	0.95	0.001
2160	SLC5A8	4540438	0.97	0.001
13559	LOC100039	6400050	0.96	0.001
9961	RRS1	6290189	0.96	0.001
7800	ADAM17	5900653	1.04	0.001
14526	PLA2G4A	1450653	1.04	0.001
21800	RCC2	3420605	1.05	0.001
5454	PIP4K2A	3140678	1.10	0.001
11405	VASH2	6980475	1.08	0.001
1568	UGT2A3	5720273	0.96	0.001
2541	INSC	3520397	1.06	0.001
2602	SNRPE	1690609	1.04	0.001
17318	DIS3L	2350170	0.96	0.001
78	CUTA	6980370	0.86	0.001
6847	BC030440	2470333	0.96	0.001
17442	1700018B2	1940010	0.95	0.001
7074	THAP7	270376	0.91	0.001
18731	HTR3A	4070608	1.06	0.001
13015	CHIA	5310152	0.97	0.001
8402	EHMT2	3610092	1.05	0.001
8665	9230110C1	2940438	0.96	0.001
22067	TBC1D2	5690343	1.06	0.001
17005	WDR31	580403	0.96	0.001
24610	AGGF1	3800546	1.04	0.001
12123	SYBL1	2450524	0.97	0.001
4568	BAT4	1660767	0.95	0.001
8597	CDC7	7380088	0.94	0.001
13441	OLFR1098	4260520	1.04	0.001
679	CLEC1A	7550022	0.96	0.001
11691	CD151	1400630	1.16	0.001
2841	EBI2	2600377	1.08	0.001
5980	H2-M10.1	20707	1.04	0.001
23098	AQP3	3360161	1.04	0.001
16483	FBLN2	6040521	1.05	0.001
24751	ALDH18A1	4150168	1.09	0.001
12308	COG8	6510296	1.07	0.001
19007	TXNDC5	1990564	0.93	0.001
8972	OLFR965	160674	1.04	0.001
11008	TRPM3	5910689	1.03	0.001
5521	TTC8	3710088	0.94	0.001
19497	MKLN1	6840537	0.94	0.001
15028	ZDHHC24	6660487	1.05	0.001
16689	LRCH4	6020091	0.96	0.001
5794	BC048403	3170722	1.06	0.001
6530	CCDC122	6200184	0.95	0.001
8574	ARF6	7200358	1.07	0.001
18613	EIF4EBP2	4390047	0.93	0.001
4982	CTPS	5050577	0.95	0.001
16317	PGA5	7160301	1.04	0.001
4919	4933405O2	7210746	1.05	0.001
21048	CALCOCO1	1440113	0.95	0.001
4027	GFOD1	7050138	1.11	0.001
6462	1110012D0	6180736	1.06	0.001

10883	GSN	4260709	1.05	0.001
21238	ZFX	4200270	0.96	0.001
16273	LOC100047	2370132	0.95	0.001
11069	PET112L	1580333	1.08	0.001
21067	ARTN	240608	1.05	0.001
22968	OLFR872	990736	1.04	0.001
4700	RNPS1	2750356	0.91	0.001
8801	ZKSCAN1	3450524	1.05	0.001
3558	PTGER4	6200670	0.95	0.001
17997	OLFR1	5960424	1.03	0.001
10352	ECE1	5700722	0.93	0.001
13840	RFNG	150725	0.95	0.001
12491	PLEKHF1	1660685	1.05	0.001
13704	CACNG2	3520411	1.08	0.001
7308	LOC225922	4570358	1.05	0.001
18640	DAPK1	4220731	0.94	0.001
18476	HIST1H1E	3190364	1.03	0.001
10203	MCTS2	1010379	1.04	0.001
23228	PRKACA	5690427	0.86	0.001
20173	PTPRM	2600367	1.03	0.001
17489	SC5D	4150747	0.96	0.001
4453	AKAP1	650735	0.96	0.001
25242	MYL2	2120356	1.08	0.001
410	MPO	6280168	0.94	0.001
1411	5730410I19	3850632	1.04	0.001
977	HIST1H2BC	270639	0.97	0.001
10462	TRAM1	5910431	0.94	0.001
1968	1700023B0	2680458	0.94	0.001
18000	HCCS	2070091	1.07	0.001
18853	RUNX3	1170241	0.96	0.001
25171	KIF5A	5490131	1.19	0.001
4495	D930036B0	4230132	1.07	0.001
10590	GPX1	1980301	1.13	0.001
12445	DIAP3	4830386	1.05	0.001
4016	OXSR1	630037	1.06	0.001
13456	CENPK	4860600	1.07	0.001
1442	BC051142	6420593	0.94	0.001
8895	DNAJC14	2370594	0.95	0.001
10448	DOT1L	2710504	0.94	0.001
24883	AGTRL1	7510653	0.95	0.001
13889	4921511K0	3180465	0.95	0.001
5219	TRUB1	1660541	1.08	0.001
23898	ANKRD50	6100274	0.96	0.001
16837	MRPL3	160370	1.05	0.001
3240	GNB2	1170390	1.06	0.001
1776	ARFGAP3	1090427	1.09	0.001
2374	SDC1	6590541	0.96	0.001
3870	4930555G0	6100561	1.03	0.001
12494	BCKDHB	3520148	0.91	0.001
17412	5730410E1	4480221	0.88	0.001
2352	RGS3	1400707	1.03	0.001
10842	TAZ	5130154	1.06	0.001
20687	2810453I06	3370367	1.07	0.001
7095	MAGEB18	2140121	0.95	0.001
1220	ZRSR1	6900195	1.05	0.001
1022	5830415L2(	6550554	1.08	0.001
22110	TRP53I11	4480288	1.08	0.001
19898	CXXC1	70577	0.90	0.001
1789	OSR2	4900168	0.94	0.001
11753	V1RJ3	7570433	1.04	0.001
25398	ERG	3830180	1.04	0.001
20798	OLFR671	4570296	0.93	0.001
16519	4933421I07	4040546	0.95	0.001
4088	RFC4	3940458	0.95	0.001
2369	AI846148	6650632	0.94	0.001
22598	SUPT5H	7400546	0.89	0.001
3818	9130008F2	4200402	1.03	0.001
7143	ZDHHC9	3450427	1.04	0.001
1670	LOC100048	3870608	0.94	0.001
5513	SLC30A9	4280524	1.06	0.001
2933	PEAR1	6130044	0.96	0.001
3477	NFATC1	450491	0.95	0.001
8552	4930573I19	2340735	0.90	0.001
13710	DTX1	6860022	0.93	0.001
19359	ZMYND19	6650743	0.95	0.001
12294	NHSL1	50341	1.06	0.001
7310	IRAK1BP1	6220369	1.04	0.001
2892	MMP1A	6380427	1.12	0.001
17680	CDKL2	3440088	0.91	0.001
