# Supplementary material for: Transcriptomic changes in the frontal cortex associated with paternal age
Source: Mol Autism. 2014 Mar 23;5:24. doi: 10.1186/2040-2392-5-24 (PMC3998024; doi:10.1186/2040-2392-5-24)
Supplement: Additional file 4 — Comparison of the top-ranked differentially expressed transcripts ( P < 0.001) across each of the six families with an old father. Shown for each transcript is the corresponding rank in the overall old vs young father group comparison. Empty cells indicate a non-significant difference. [file 2040-2392-5-24-S4.pdf]

Additional File 3 – Comparison of the top-ranked differentially-expressed transcripts (p<0.001) across each of the six families with an old father. Shown for each transcript is the corresponding rank in the overall old vs. young father group comparison. No data indicates a non-significant difference.

		Family 1		Family 2		Family 3		Family 4		Family 5		Family 6	
Probe	Gene	Fold Change	P Value	Fold Change	P Value	Fold Change	P Value	Fold Change	P Value	Fold Change	P Value	Fold Change	P Value
10181	TMPO									1.06	0.000592	0.96	0.001
50341	NHSL1					1.06	0.001					0.92	1.93E-07
110048	FBN1					1.06	0.000388					1.06	0.000704
360431	SERPINA1A					0.91	5.66E-07					0.93	9.71E-06
450020	SLC2A5			0.93	2.10E-05							0.94	0.00031
450400	ATRX					1.04	0.000454					0.94	2.15E-05
510059	FRYL					1.11	3.78E-09			1.06	8.37E-06		
510735	ANGPT1					0.89	1.11E-10			0.96	0.000308		
540075	CYP4F16					0.94	0.000405					0.9	7.83E-06
610075	TIMM9					1.07	0.000963			0.94	0.001		
630162	NIPSNAP3A			0.95	0.000666							0.96	0.000775
650630	SPNB2									1.15	0.000529	1.07	0.001
670050	A430107D22RIK					1.09	0.001					1.06	0.000213
670647	RGS9	0.78	0.000174					0.81	0.000459				
770372	RWDD2B					1.08	8.50E-05					0.94	7.00E-04
830026	B230118H07RIK	0.96	0.001			0.94	8.69E-07						
830563	PRPF40B					0.94	3.29E-05			1.07	3.33E-05		
940180	PIK3IP1					0.94	0.000855					1.07	0.001
990349	MOSPD1			1.07	0.000291	0.91	6.44E-06						
1010435	GSTO1			0.92	0.000314					1.07	0.000674		
1010446	MEX3C	1.04	0.001			1.04	0.000868						

1010653	DNAJB4									1.04	1.43E-06	1.07	4.19E-07
1030768	HCFC1R1									0.96	7.49E-05	0.96	0.000312
1050397	OLFR559	1.05	1.57E-05									1.03	0.000784
1050521	ADO			0.97	0.001							1.07	1.13E-06
1070187	6430527G18RIK									0.94	0.001	0.94	0.000191
1070438	DCUN1D3					1.09	2.27E-06					1.07	3.34E-05
1240324	RAB8A	0.93	0.001			1.08	0.000488						
1410091	CALU	1.04	0.001							1.05	0.001		
1450129	ZP2					1.07	3.12E-05					1.05	0.000795
1450653	PLA2G4A									0.96	0.000823	1.04	0.001
1510433	D030070L09RIK					1.1	0.000362			1.05	0.000645		
1710474	DGKQ	1.06	0.000308			0.95	0.000389						
1770437	ZIK1									1.04	0.000214	0.95	2.56E-05
1780079	IL7R	0.95	0.001	0.96	0.000739								
1780307	CCL25					1.07	0.000265					1.07	0.000657
1990731	DDT					0.95	9.81E-05					0.95	0.000162
2030025	TMOD2					0.96	0.000589					0.95	0.000139
2030088	DEFB19					1.05	0.000683					1.05	0.001
2060612	KCTD10					0.97	3.00E-04					0.94	1.11E-06
2120356	MYL2					1.08	0.001			0.91	0.00021		
2190139	SRPX2	1.04	0.000105									0.97	0.000712
2190612	WIPI2			0.91	0.001	0.91	0.000741						
2260398	ZBTB7C					1.06	6.21E-05					1.06	0.000904
2320402	PPARG									0.93	0.001	0.95	5.27E-05
2450463	ARL3			1.08	0.000846			1.15	8.83E-07				
2470044	DEXI	0.91	0.000458	0.94	0.00021								
2470343	LCORL					0.96	0.000245					0.92	0.000462
2480243	HERC3					1.05	0.001	0.91	0.001				

2480255	TNFSF11			1.05	0.000403	1.07	0.000304						
2510500	TGFB3					0.93	4.60E-06					0.96	0.000925
2600253	A930025D01RIK	0.95	6.88E-05							0.96	0.000829		
2600437	LASS4									0.93	0.000167	0.91	2.65E-07
2650370	RALBP1			0.89	0.00019							0.87	2.90E-05
2680682	MEI1					1.04	0.000189	0.96	0.000254				
2690240	TRABD					1.11	1.71E-06			1.07	0.00092		
2710201	OLFR92							1.05	0.001			1.06	2.60E-05
2710465	EG317677					0.94	0.000363					0.94	1.01E-05
2810722	LAX1	0.95	5.79E-05									0.95	7.62E-05
2850349	RAB17	0.95	5.11E-05			0.96	0.000414						
2900661	BC026585					1.09	0.000836	1.09	0.000426				
2940097	PSME3	1.18	3.64E-05			0.87	6.56E-05						
2970685	RABL3	0.92	0.001					0.94	0.000807				
3060398	TRPC4			0.94	0.000739							0.96	0.000621
3060730	FTMT	1.03	0.001									0.96	0.000243
3120392	STMN1			0.93	0.001							0.93	0.000319
3170148	ATP2B2					0.93	0.001	0.92	0.000327				
3170487	CD300LF									1.05	0.001	0.95	0.001
3290692	RIPPLY1					1.04	0.000528					1.04	4.94E-06
3370189	AB041803							1.05	0.000843			1.04	0.000837
3370367	2810453I06RIK					1.07	0.001			0.92	0.000234		
3370450	FBXL20			0.96	4.61E-05							0.95	1.88E-06
3390328	SLC35C1					0.92	2.13E-06					0.93	1.28E-05
3390731	NCAM1							0.93	0.000661	1.12	3.19E-06		
3420291	FECH							1.06	0.000186	1.05	0.001		
3440301	POLM	1.05	0.001							1.05	0.001		
3450010	TK1	0.94	6.66E-05									0.91	8.48E-08

3460575	CD109	0.94	2.45E-05					0.93	0.000864				
3460731	PNKD					1.05	0.000321			0.95	0.000596		
3800292	OLFR482					0.95	9.46E-06					0.94	0.000707
3870646	TCP11L1					0.94	7.11E-05					0.95	0.000281
3890500	GPC3			0.94	4.11E-05							0.95	0.000519
3940286	ADAM23					0.9	0.000141					1.13	0.000449
4010129	D6WSU163E					1.06	0.000286			1.08	0.001		
4060358	OLFR1013					1.05	7.58E-06					0.96	0.000633
4070719	GUCY2C							1.04	0.001			0.95	0.001
4180379	NDUFS2					1.06	0.000263	1.06	0.000536				
4210176	LGALS12	1.06	0.001			1.07	1.35E-07						
4220519	NDUFA8					1.1	0.000916			0.93	0.000519		
4290019	RBM12					0.95	0.000396					0.93	3.56E-05
4290626	OLFR1497					1.05	0.000582	1.06	0.001				
4640639	OTTMUSG00000010673					0.94	3.03E-05					0.94	7.47E-05
4730343	OLFR398					0.96	1.46E-05					1.07	3.02E-08
4760612	SLC14A2									1.05	0.000126	1.09	5.03E-05
4810017	SCGB3A1									0.96	0.000729	0.94	3.44E-06
4830040	NRCAM					1.06	0.001					1.11	5.14E-06
4830725	2310047O13RIK	1.06	5.93E-05					1.07	0.000926				
4920594	UCP2					1.08	5.68E-06					1.05	0.001
5090739	CLEC16A							1.06	0.000469	1.07	0.000167		
5130139	TGFB1					0.93	0.000817					0.95	0.000536
5130154	TAZ					1.06	0.001					0.94	0.00062
5220181	EYA3					1.05	0.000892					1.06	0.00019
5490603	AZI1					0.94	7.06E-05			1.06	0.000158		
5550482	TBC1D2	1.04	0.001									1.05	0.000733
5570379	ACAD10	1.03	0.001									1.06	2.63E-06

5670373	CYHR1							0.95	0.00045			1.06	0.000285
5700722	ECE1	1.13	1.62E-05			0.93	0.001						
5720288	NR5A1					0.91	5.06E-08	0.92	7.63E-05				
5810170	ASCC3L1									0.94	0.001	0.93	0.000323
5820681	PRKCZ					1.08	8.29E-05	1.12	0.000348	0.92	8.79E-05		
5820707	JMY					0.95	0.000367					0.94	1.82E-06
5860253	FIGLA					1.05	0.00012					1.06	1.64E-05
5860377	USP15					0.94	2.91E-05	0.95	1.23E-05				
5890139	PDK2	1.07	0.000159									1.08	2.64E-05
5890762	HTR3B									0.94	9.43E-06	0.91	7.40E-07
5900465	4921530G04RIK			0.89	4.81E-06							0.93	0.000636
5900653	ADAM17					0.94	0.000996					1.04	0.001
5910220	OSMR									1.06	2.30E-05	1.08	4.08E-05
5960050	SLC25A11			0.88	9.91E-06			0.89	2.25E-05				
6040707	GLI3					1.04	5.93E-05	1.05	4.87E-05				
6220612	APH1B	1.06	0.001							1.06	7.88E-05		
6280168	MPO					0.94	0.001	0.95	0.00078				
6280520	TRIP12					0.92	0.000137					0.93	0.000383
6330102	SMC1A					0.93	4.09E-07	0.97	0.001	1.03	0.001		
6330451	P2RX5	0.95	0.000227			1.05	7.20E-05						
6350402	HS6ST1					0.88	2.15E-06					0.89	3.49E-05
6350437	KATNAL1			0.93	0.000209	0.93	3.91E-05						
6370674	MYST1					1.07	0.001					0.93	9.01E-05
6400025	LARP4	0.95	0.000488					0.94	2.52E-05				
6480477	EG331493	0.93	6.60E-07									0.94	7.20E-06
6510427	ZFP523					1.09	0.000525			0.91	0.000417		
6520484	EIF4G2					1.15	0.000675					1.11	3.43E-05
6550309	FXYD6	0.84	0.000436			0.82	6.79E-06						

6560152	NDUFA5					1.15	1.25E-05					1.07	0.000811
6560692	ST6GALNAC4					0.94	2.00E-04	0.94	0.000856				
6590564	GABRA1	1.05	0.000247	1.08	2.27E-05								
6650220	ANXA10	1.03	0.000137			1.03	1.90E-05						
6650484	TBPL2					0.95	0.000397					1.06	0.000158
6660152	LOC100048726									1.05	0.001	1.1	2.43E-07
6770369	COL17A1							1.06	9.63E-06			0.95	0.00084
6840328	OTOR					0.93	8.78E-08			0.96	0.000737		
6900133	MTSS1			0.95	0.000825							1.04	5.27E-05
7100180	TTLL1	0.94	0.001			1.07	0.000255						
7100519	CLK4			1.09	7.62E-07							1.06	8.97E-05
7150670	MFSD8							1.07	5.57E-05			1.05	0.000387
7200035	FGFR3					1.11	0.000696					1.12	0.000649
7210632	PDE1A			0.86	0.000551	0.9	0.001						
7210746	4933405O20RIK			1.04	1.88E-05							1.05	0.001
7380382	ZC3H18	1.11	0.000361					1.09	7.51E-07				
7400546	SUPT5H	1.17	0.000428			0.89	0.001						
7400678	C330005M16RIK					0.95	3.20E-05			0.96	0.000294		
7510609	LOC100047391									0.95	7.16E-05	0.95	0.000996
7560097	NARG2							1.05	0.000329			1.08	1.80E-06
7560228	EAR10							0.96	0.000108	0.96	0.000323		
7560291	JUN					1.09	0.000896					1.11	0.000757
